# Characterization of carbon fluxes, stock and nutrients in the sacred forest groves and invasive vegetation stands within the human dominated landscapes of a tropical semi-arid region

**DOI:** 10.1038/s41598-024-55294-0

**Published:** 2024-02-24

**Authors:** R. V. Akil Prasath, R. Mohanraj, K. R. Balaramdas, A. Jhony Kumar Tagore, P. Raja, A. Rajasekaran

**Affiliations:** 1 Department of Environmental Science and Management, Bharathidasan University, Tiruchirappalli, 620024 India; 2 St. Joseph’s College, Tiruchirappalli, India; 3 Institute of Forest Genetics and Tree Breeding, Coimbatore, 641002 India

**Keywords:** Biogeochemistry, Climate-change ecology, Ecophysiology, Ecosystem services, Invasive species, Ecology, Plant sciences, Climate sciences, Environmental sciences

## Abstract

In the semi-arid plains of Southern India, outside the protected area network, sacred groves forests and the barren lands invaded by *Prosopis juliflora* are reckoned to be the major greenery, but have homogenous and heterogeneous vegetation respectively. This study attempted to compare 50 Sacred Groves Stands (SGS) and 50 monodominant *Prosopis juliflora* Stands (PJS) for the functional diversity, evenness, floral diversity, carbon stock and dynamics, carbon-fixing traits, dendrochronology of trees, soil nutrient profiles, and soil erosion. Quadrat sample survey was adopted to record stand density, species richness, abundance, basal area and leaf area index; composite soil samples were collected at depths 0–30 cm for nutrient profiling (N, P, K, and OC). Photosynthesis rate (µmole co_2_ m^2^/sec), air temperature (°c), leaf intracellular co_2_ concentration (ppm), ambient photosynthetic active radiation (µmole m^2^/sec), transpiration rate (m. mole H_2_O m^2^/sec) were determined for the 51 tree species existed in SGS and PJS using Plant Photosynthesis system. Structural Equation Model (SEM) was applied to derive the carbon sequestering potential and photosynthetic efficiency of eight dominant tree species using vital input parameters, including eco-physiological, morphological, and biochemical characterization. The Revised Universal Soil Loss Equation (RUSLE) model, in conjunction with ArcGIS Pro and ArcGIS 10.3, was adopted to map soil loss. Carbon source/sink determinations inferred through Net Ecosystem Productivity (NEP) assessments showed that mature SGS potentially acted as a carbon sink (0.06 ± 0.01 g C/m^2^/day), while matured PJS acted as a carbon source (−0.34 ± 0.12 g C/m^2^/day). Soil erosion rates were significantly greater (29.5 ± 13.4 ton/ha/year) in SGS compared to PJS (7.52 ± 2.55 ton/ha/year). Of the eight selected tree species, SEM revealed that trees belonging to the family Fabaceae [*Wrightia tinctoria* (estimated coefficient: 1.28, *p* = 0.02) > *Prosopis juliflora* (1.22, *p* = 0.01) > *Acacia nilotica* (1.21, *p* = 0.03) > *Albizia lebbeck* (0.97, *p* = 0.01)] showed comparatively high carbon sequestering ability.

## Introduction

Beyond the authorized protected area network, the biodiversity-rich terrestrial ecosystems entailing minimal land area have been subject to indiscriminate anthropogenic exploitation worldwide since the late Holocene. The extent of degradation hinges on the proximity of such ecosystems to human habitations, urban areas and croplands^1–3^ and the activities. In the current context of global change and its tangible effects, as long as the biodiversity, the vegetation structure and the ecological setup in such high-value ecosystems remain intact, the buffering potential for the adjoining area to weather extremities is likely to be higher, and the loss is minimal. However, human settlement and land use practices, which invariably naturalize several non-native plants and animals, gradually lead to the loss of endemic biota and the transformation of such native ecosystems^4,5^.

Across the world, relics of few native forests distributed along the urban peripheries, are safeguarded by the local community to a great extent over centuries despite anthropogenic disturbances^6–12^. In Africa, South Asia and Southeast Asia, the motive for protecting these vegetation patches, revered as sacred forest grooves, has deep socio-cultural and spiritual linkages. However, whether such sacred forest grooves are fragmented remnants of earlier forest stretches or regenerated forests remain unclear, although a study from the Western Ghats of India ascertains the phenomenon of regeneration driven by social, ecological and economic necessities, dating back to 400 Years Before Present (YBP)^13^. Certain plants and animal species are also protected due to religious and cultural taboos^14^. Such an attitude towards protecting certain natural species or habitats had perhaps been in practice among many indigenous communities for millennia, possibly due to their intimate experiential familiarity with various species and the tangible and intangible ecosystem services they maybe derive from their proximate ecological systems.

Ecosystem services (ESS) are the indispensable benefits derived from natural ecosystems that serve as humans' basic life support systems directly or indirectly^15–18^. A wide spectrum of services, supporting, provisioning, regulating, and cultural services, directly influence the life quality^19–21^. Notwithstanding the size, even small systems like sacred groves deliver multiple ecosystem services, including carbon sequestration, protection against flash floods, minimizing the urban heat island effect, attenuating noise pollution, and improving the overall environmental quality^22–28^. Apparently, certain regulating services like local climate regulation and soil erosion are significant^29,30^. The role of sacred groves in replenishing the ground water is also reported^31^. Sacred groves exhibiting a complex and intricate web of species interactions also help effectively control the expansion of predatory, parasitic pests^32,33^.

India has a heritage of more than 100,000 sacred groove forests, overarching cultural taboos and religious beliefs of the local community^34^. The tropical forests of Southern India are renowned for their rich and unique endemic flora, which, in many cases, can trace its origins back to the end of the Cenozoic era approximately 1.6 million years ago^8^. Numerous scientific investigations have examined the regulating ecosystem services, including carbon sequestration efficacy^30,35–40^, primary productivity and floral and faunal species richness^10,14,41–43^. However, most of these studies had a superficial approach to analyzing the values of ecosystem services and lacked a comprehensive assessment of the functional traits of the vegetation. In this context, we attempted to understand the holistic functions of the sacred groves in a semi-arid region of Southern India. Buoyed by the rapid economic growth in recent decades, the urban regions in India have witnessed massive transformation and expansion, which has direct implications for the natural ecosystems within its ambit and peripheries. Such expansion drives had also imposed a considerable dent in the sacred grooves of the adjoining regions. In the fluvial plains of arid and semi-arid regions of India, besides the croplands and protected forests network, sacred forest groves and the landscapes invaded by the exotic species (*Prosopis juliflora, Acacia auriculiformis, Lantana camara, Eichorania crassipes*) are the available green patches. Among the invasive vegetation, *Prosopis juliflora* stands are predominant and persistent throughout the year despite human interventions (firewood and fodder); and many *Prosopis juliflora stands* are remaining intact for over four decades. Although *Prosopis juliflora* infestation is widely criticized for many ill effects, it is also credited for a few ecosystem services like carbon sequestration and soil erosion control^44–46^. However, regulatory ecosystem services in such *Prosopis juliflora* Stands (PJS), like groundwater recharge, floral and faunal richness, etc., remain low^47–50^. In the policy maker perspective, presently *Prosopis juliflora* invasion is considered as menace and is poised for removal in many locations across India. At this juncture the present study attempts to compare carbon fluxes, carbon stock and nutrients in these two types of vegetative stands (Sacred Groves Stands and *Prosopis juliflora* Stands) which has minimal human intervention but lying outside the protected area. And, how does the monodominant PJS fare in terms of carbon sequestration, soil nutrient enrichment and soil loss, over the heterogeneous SGS, is another important rationale for comparing these two vegetation types. Accordingly, present investigations mainly focused on species richness, soil nutrient enrichment, carbon stock (above and below ground), comparative photosynthetic efficiency of tree species, morphological (Stomatal density) and physiological carbon sequestering functional traits (photosynthesis rate, air temperature (°c), leaf intracellular co_2_ concentration, ambient photosynthetic active radiation, and transpiration rate), carbon flux dynamics in Sacred Grove Forests & *Prosopis juliflora* stands. In addition, to differentiate the study sites based on the anthropogenic influence, ambient CO_2_ levels across the study sites has been considered as an indirect indicator reflect the contamination from fossil fuel based emissions (from transportation and industries).

## Results

### Ecological diversity and soil physicochemical profile of Sacred Groves Stands (SGS) and Prosopis juliflora Stands (PJS)

**Figure 1:**
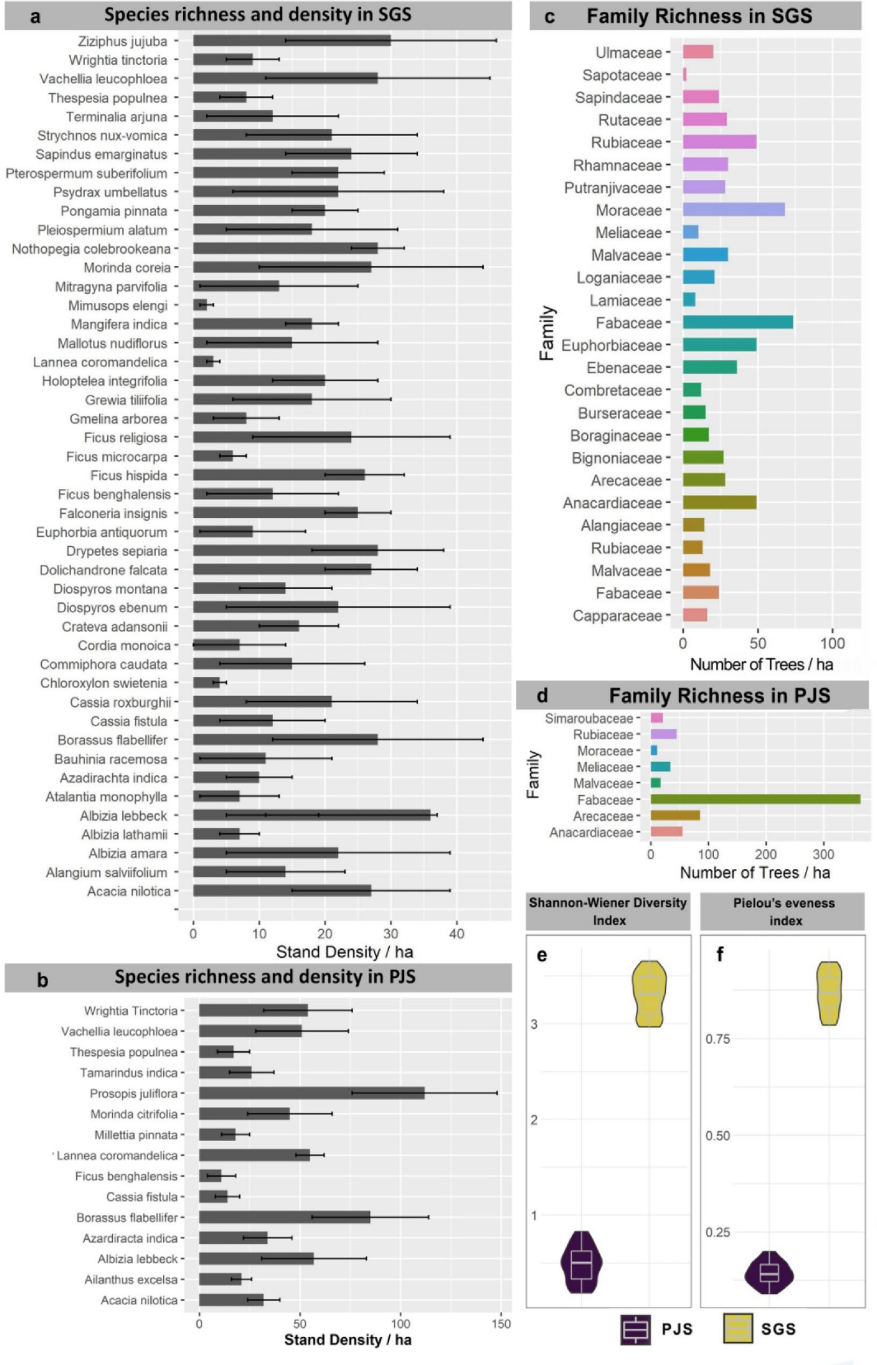
Ecological Profile of Sacred Groves Stands (SGS) and *Prosopis juliflora* Stands (PJS) located across the central region of Tamil Nadu. (**a**) Tree species richness and corresponding stand density/ha in SGS. (**b**) Tree species richness and density/ha in PJS. (**c**) Family richness in SGS. (**d**) Family richness in PJS. (**e**) Shannon wiener diversity index (H-index). (**f**) Pielou’s evenness index (E-index).

Our study comparing ecological indices between PJS and SGS revealed highly significant differences in both diversity and evenness; Shannon–Wiener diversity index values indicated high diversity (3.65 ± 0.52) in SGS when compared to PJS (0.46 ± 0.33) (*p* < 0.01***—Man-Whitney U Test) (Fig. 1e). Pielou’s evenness index also demonstrated a significant contrast (*p* < 0.01***—Man-Whitney U Test), with SGS displaying higher evenness (0.81 ± 0.13). PJS showing lower evenness (0.15 ± 0.08) is indicative of the single species (*P. juliflora*) dominance (Fig. 1f) and its implications on the overall floral diversity.

Tree species richness of SGS was considerably higher (34 ± 12) than PJS (9 ± 6). In SGS *Ziziphus jujuba* (32 ± 16 /ha) was found to be most abundant, followed by *Vachellia leucophloea* (25 ± 15 /ha), *Borassus flabellifer* (27 ± 16 /ha), *Morinda coreia* (26 ± 16 /ha), *Acacia nilotica* (27 ± 11 /ha), *Ficus religiosa* (24 ± 14 /ha), *Diospyros ebenum* (21 ± 19 /ha), *Albizia amara* (21 ± 17 /ha), etc. (Fig. 1a). In PJS, *Prosopis juliflora* (104 ± 36 /ha) was the most dominant tree, followed by *Borassus flabellifer* (63 ± 38 /ha) and *Albizia lebbeck* (62 ± 21 /ha) (Fig. 1b). Moreover, the family richness of the SGS was also recorded as high (range: 9–26), in contrast to PJS (range: 5–8). However, in both vegetation types, species belonging to the Fabaceae family (legumes/peas) were the most abundant (Fig. 1c,d).

### Soil nutrient profile of sacred groves stands (SGS) and prosopis juliflora stands (PJS)

**Figure 2:**
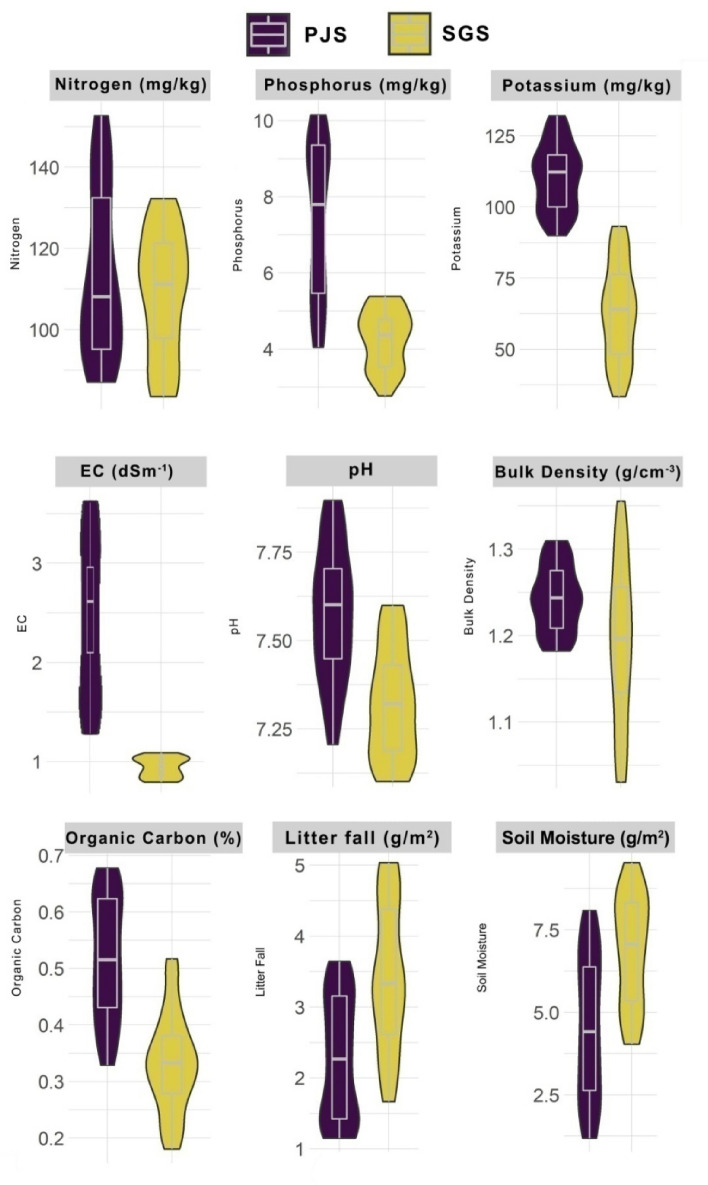
Soil characteristics and nutrient profile in the Sacred Groves Stands (SGS) and *Prosopis juliflora* Stands (PJS) across the central region of Tamil Nadu.

Soil nutrient profile viz. Available Nitrogen, Available Phosphorus, Available Potassium, Electrical Conductivity, pH, Bulk Density, Soil Organic Carbon, and Soil Moisture varied significantly (Kruskal- Wallis rank sum test—*p* < 0.01***) between SGS and PJS. In PJS, comparatively high quantities of Available Nitrogen, Phosphorus, Potassium, Electrical Conductivity, pH and Organic carbon were recorded. Conversely, Soil Bulk Density and soil Moisture were observed high in SGS than in PJS (Fig. 2). Litter fall was also found to be higher in SGS (1.6–5.0 g/m^2^) than in PJS (1.1–3.6 g/m^2^) (Fig. 2).

### Carbon stock and dynamics in SGS and PJS

**Figure 3:**
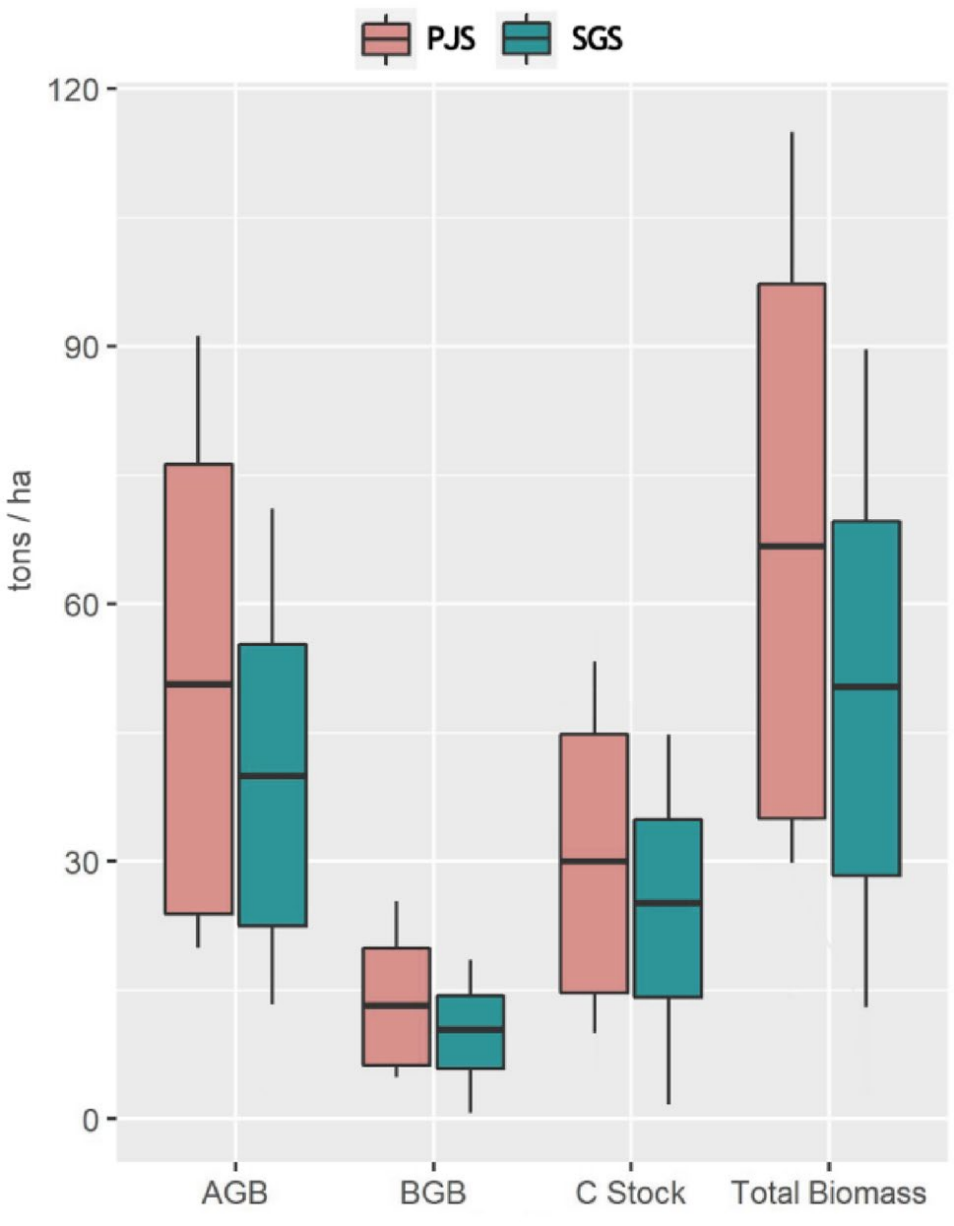
AGB, BGB, Total Biomass, and Carbon Stock of Sacred Groves Stands (SGS) and *Prosopis juliflora* Stands (PJS) across the central region of Tamil Nadu.

Tree stand density in PJS stood at 1056 ± 474/ha, while in SGS, it is comparatively low (584 ± 331 /ha). Similarly, in SGS, the basal area (25 ± 11 m^2^/ha) is also lower than PJS (38 ± 18 m^2^/ha). The leaf area index of PJS was higher (2.69 ± 0.54 m^2^/m^2^) than SGS's (1.34 ± 0.66 m^2^/m^2^). Concomitantly, Above Ground Biomass (AGB), Below Ground Biomass (BGB) and Carbon Stock (CS) were observed to be high in PJS (AGB = 56 ± 35 tons/ha; BGB = 16.2 ± 10.3 tons/ha; CS = 32 ± 20.6 tons/ha) than SGS (AGB = 45 ± 31 tons/ha; BGB = 9.6 ± 8.8 tons/ha; CS = 27.3 ± 22.4 tons/ha); Kruskal Wallis test confirmed the significance with *p* < 0.05*for AGB; *p* < 0.05* for BGB; and *p* < 0.05* for Carbon Stock between PJS and SGS (Fig. 3).

**Figure 4:**
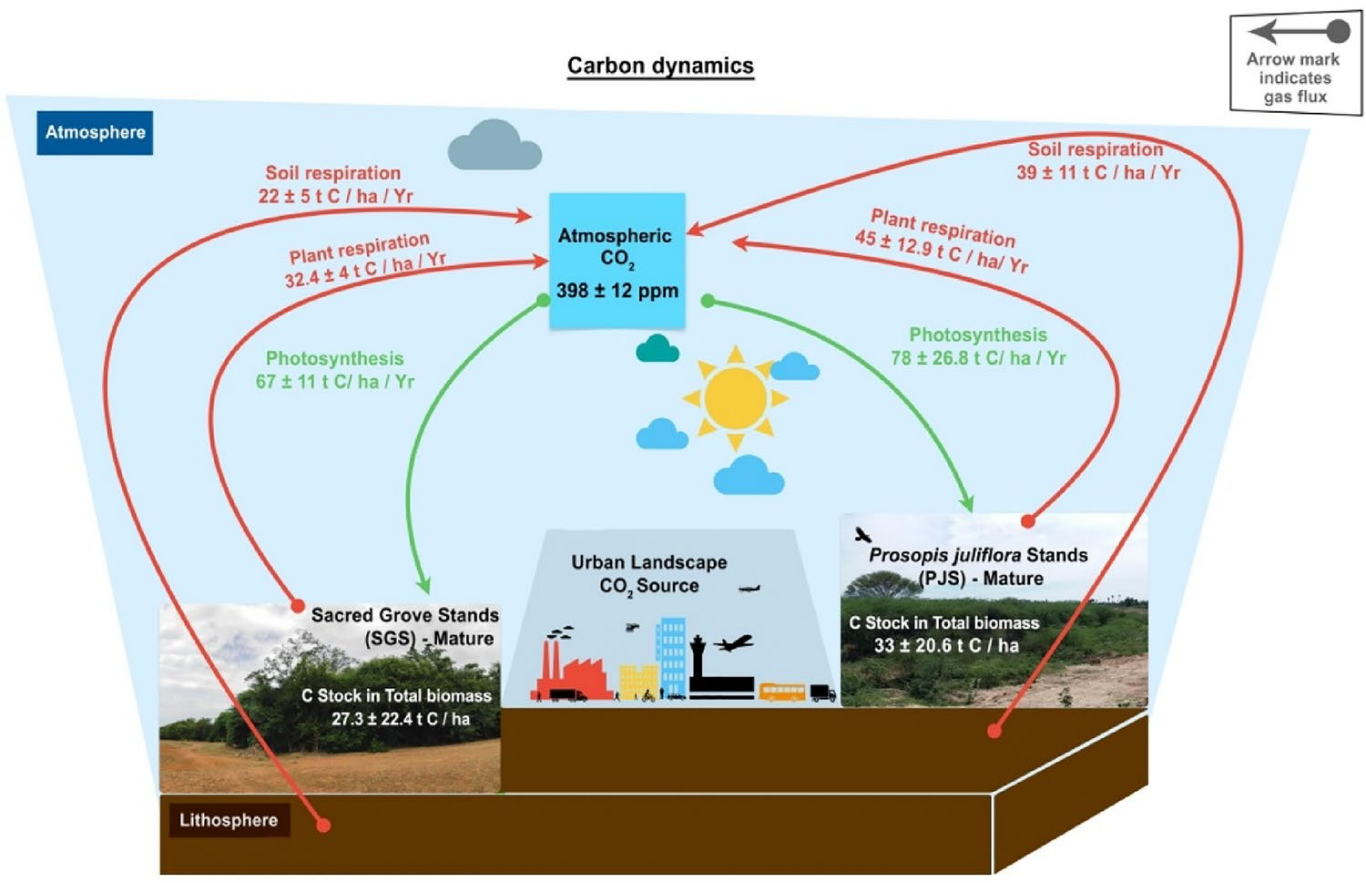
Carbon dynamics in Sacred Groves Stands (SGS) and *Prosopis juliflora* Stands (PJS) across the central region of Tamil Nadu.

In the PJS, the presence of younger plants in young stands showed comparatively higher values of Net Ecosystem Productivity (NEP) and could act as a carbon sink (0.0012 ± 0.0004 g C/m^2^/day). However, the matured stands within PJS showed a shift towards carbon source, as evident in the NEP value of −0.0006 ± 0.0003 g C/m^2^/day; the old growth PJS, recording an NEP value of −0.0034 ± 0.0012 g C/m^2^/day, clearly indicates the transition towards carbon source. In SGS, among all the age groups, Net Ecosystem Productivity values (Young: 0.07 ± 0.02 g C/m^2^/day, Mature: 0.13 ± 0.02 g C/m^2^/day, Old: 0.06 ± 0.01 g C/m^2^/day) identified the stands to be carbon sinks for a very prolonged period compared to PJS (Fig. 4 and Table 1).

**Table 1:** Ecosystem productivity, carbon sink efficiency and carbon dynamics for Young, Mature, Old Sacred Groves Stands (SGS) and *Prosopis juliflora* Stands (PJS) across the central region of Tamil Nadu.

	Young (15–25 years)		Mature (25–45 years)		Old (45–75 years)	
Productivity	PJS	SGS	PJS	SGS	PJS	SGS
GPP [Gross Primary Productivity] (g C / m^2^ / Day)	0.0084 ± 0.0032	0.0038 ± 0.0014	0.0078 ± 0.0026	0.0067 ± 0.0011	0.0048 ± 0.0018	0.0055 ± 0.0015
Plant respiration—flux (g C / m^2^ / Day)	0.0053 ± 0.0024	0.0022 ± 0.0011	0.0045 ± 0.0012	0.0032 ± 0.0004	0.0035 ± 0.0012	0.0037 ± 0.0007
NPP[Net Primary Productivity] (g C / m^2^ / Day) *[NPP* = *GPP—PR]*	0.0031 ± 0.0014	0.0016 ± 0.0003	0.0033 ± 0.0014	0.0035 ± 0.0007	0.0013 ± 0.0004	0.0035 ± 0.0008
Soil respiration—flux (g C / m^2^ / Day)	0.0019 ± 0.0007	0.0009 ± 0.0001	0.0039 ± 0.0011	0.0022 ± 0.0005	0.0047 ± 0.0016	0.0029 ± 0.0009
NEP [Net Ecosystem Productivity] *[NEP* = *NPP—SR]*	0.0012 ± 0.0004	0.0007 ± 0.0002	−0.0006 ± 0.0003	0.0013 ± 0.0002	−0.0034 ± 0.0012	0.0006 ± 0.0001
Mann Whitney U Test (*P* < 0.05)	*P* < 0.05**		*P* < 0.01***		*P* < 0.01***	

Significance Codes: 0 ‘***’ 0.001 ‘**’ 0.01 ‘*’ 0.05 ‘.’ 0.1 ‘ ’ 1.

*If NEP is positive, then the ecosystem is a sink. If NEP is negative, the ecosystem is a source.

### Soil erosion in sacred groves stands (SGS) and prosopis juliflora stands (PJS)

**Figure 5:**
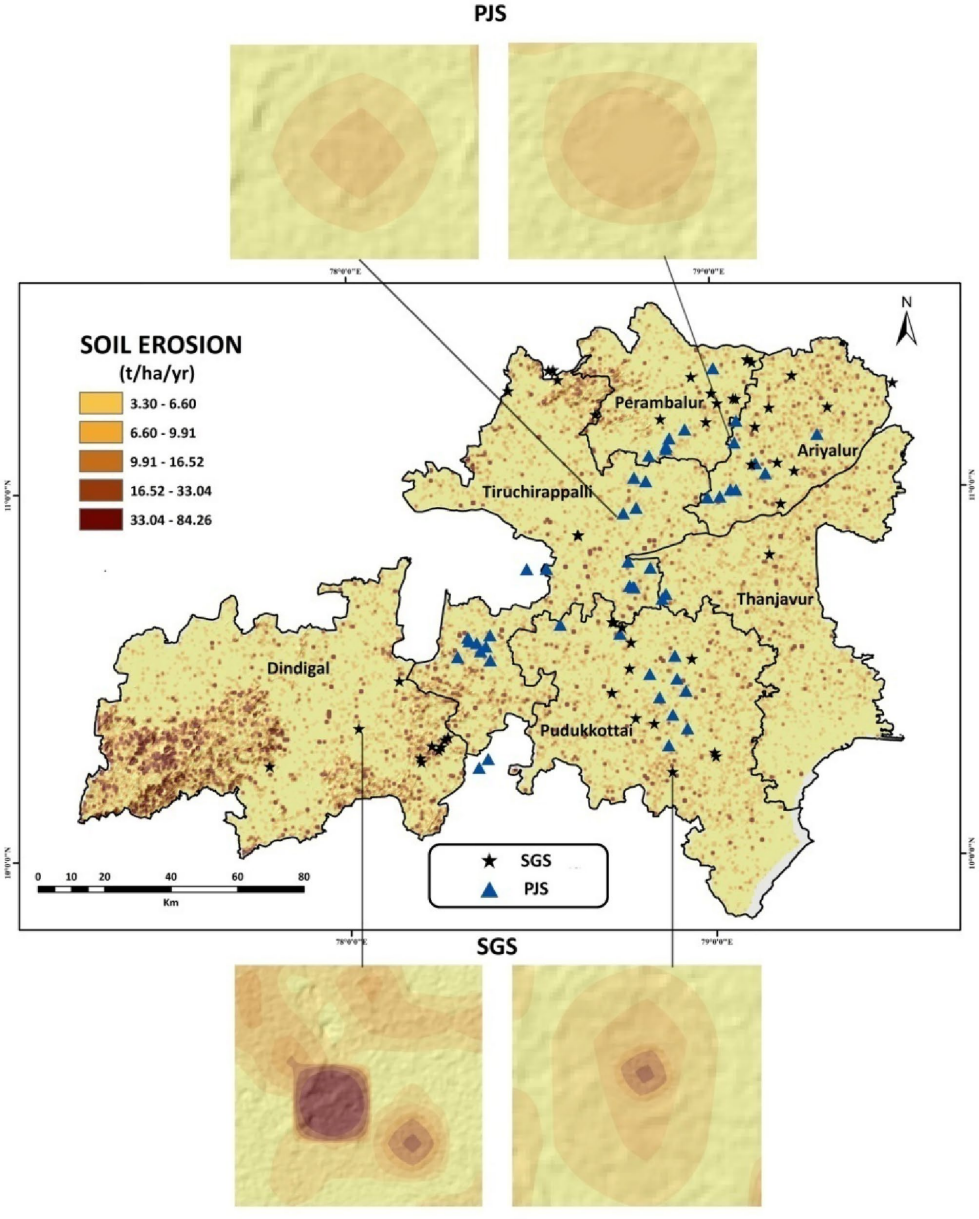
RUSLE model for soil erosion in Sacred Groves Stands (SGS) and *Prosopis juliflora* Stands (PJS) across the central region of Tamil Nadu.

RUSLE model revealed that soil erosion rates were significantly greater (29.5 ± 13.4 ton/ha/year) in SGS compared to PJS (7.52 ± 2.55 ton/ha/year) (Table 2). This difference between SGS and PJS might be attributed to the presence of a well-developed root system and higher soil organic carbon content and densely covered canopy in *Prosopis juliflora* Stands. These findings emphasize the importance of PJS in mitigating soil erosion, and any restoration plans in such invasive vegetation stands should consider retaining this characteristic feature (Fig. 5).

**Table 2:** Soil erosion rate in Sacred Groves Stands (SGS) and *Prosopis juliflora* Stands (PJS) across the central region of Tamil Nadu.

	Soil Erosion (ton / ha / year)	Kruskal Wallis Test (*p* < 0.05)
PJS	7.52 ± 2.55	0.01***
SGS	29.5 ± 13.4	

Significance Codes: 0 ‘***’ 0.001 ‘**’ 0.01 ‘*’ 0.05 ‘.’ 0.1 ‘ ’ 1.

Over all the functional diversity of SGS was found to be high (0.73 ± 0.09) in comparison to PJS (0.538 ± 0.264), but the functional evenness found to be comparatively high in PJS. This could be attributed to the high stand density of *Prosopis juliflora* (Tables S3 and S4).

### Photosynthetic rate of SGS and PJS in urban–rural gradient

The overall photosynthetic rate of *Prosopis juliflora* Stands (PJS) was notably higher with 3.90 micro moles CO_2_/m^2^/sec, in contrast to Sacred Groves Stands (SGS) which had a lower rate of 3.23 micro moles CO_2_/m^2^/sec (Fig. S2). Similarly, the transpiration rate in PJS was found to be significantly higher than SGS, as evidenced by the Kruskal–Wallis test. Higher transpiration rate in PJS could be possibly linked to the thick coppices of PJS, although individual *Prosopis juliflora* tree may transpire less if compared to a native tree in SGS (Fig. S3 and Table S3).

In the urban–rural gradient, there were significant variations in ambient CO_2_ levels and correspondingly photosynthesis rates also varied irrespective of SGS and PJS (Kruskal–Wallis test: *p* < 0.01). However, within Urban PJS and SGS, the Wilcoxon rank sum test revealed no significant differences (*p* > 0.05), same consistency was observed within Rural PJS and SGS (*p* > 0.05). Urban PJS demonstrated a notably higher photosynthesis rate of 3.43 ± 0.79 Micro mole CO_2_/m^2^/sec, followed by Urban SGS, Rural PJS, and Rural SGS; Wilcoxon rank sum test underscored a clear distinction among these four categories, with statistical significance (*p* < 0.05) (Table 3).

**Table 3:** Photosynthetic rate of Sacred Groves Stands and *Prosopis Juliflora* Stands across urban, rural gradient across the central region of Tamil Nadu. (n = number of SGS and PJS classified as urban and rural).

	CO_2_ (ppm)	Kruskal Wallis Test (*p* < 0.05)	Photosynthesis (Micro mole CO_2_/m^2^/sec)	Kruskal Wallis Test (*p* < 0.05)	Species richness	Kruskal Wallis Test (*p* < 0.05)
Urban PJS (n = 5)	410 ± 2.1^a^	*P* < 0.01	3.43 ± 0.79^a^	*P* < 0.01	6 ± 3	*P* < 0.01
Urban SGS (n = 9)	410 ± 2.5^a^		3.11 ± 0.38^b^		20 ± 7	
Rural PJS (n = 45)	386 ± 2.4^b^		1.91 ± 1.35^c^		8 ± 5	
Rural SGS (n = 41)	385 ± 6.7^b^		1.45 ± 1.07^d^		31 ± 15	

Significance Codes: 0 ‘***’ 0.001 ‘**’ 0.01 ‘*’ 0.05 ‘.’ 0.1 ‘ ’ 1.

### Measuring carbon sequestration potential of selected tree species through structural equation modeling by integrating morphological, biochemical, and eco-physiological carbon fixing functional traits.

**Figure 6:**
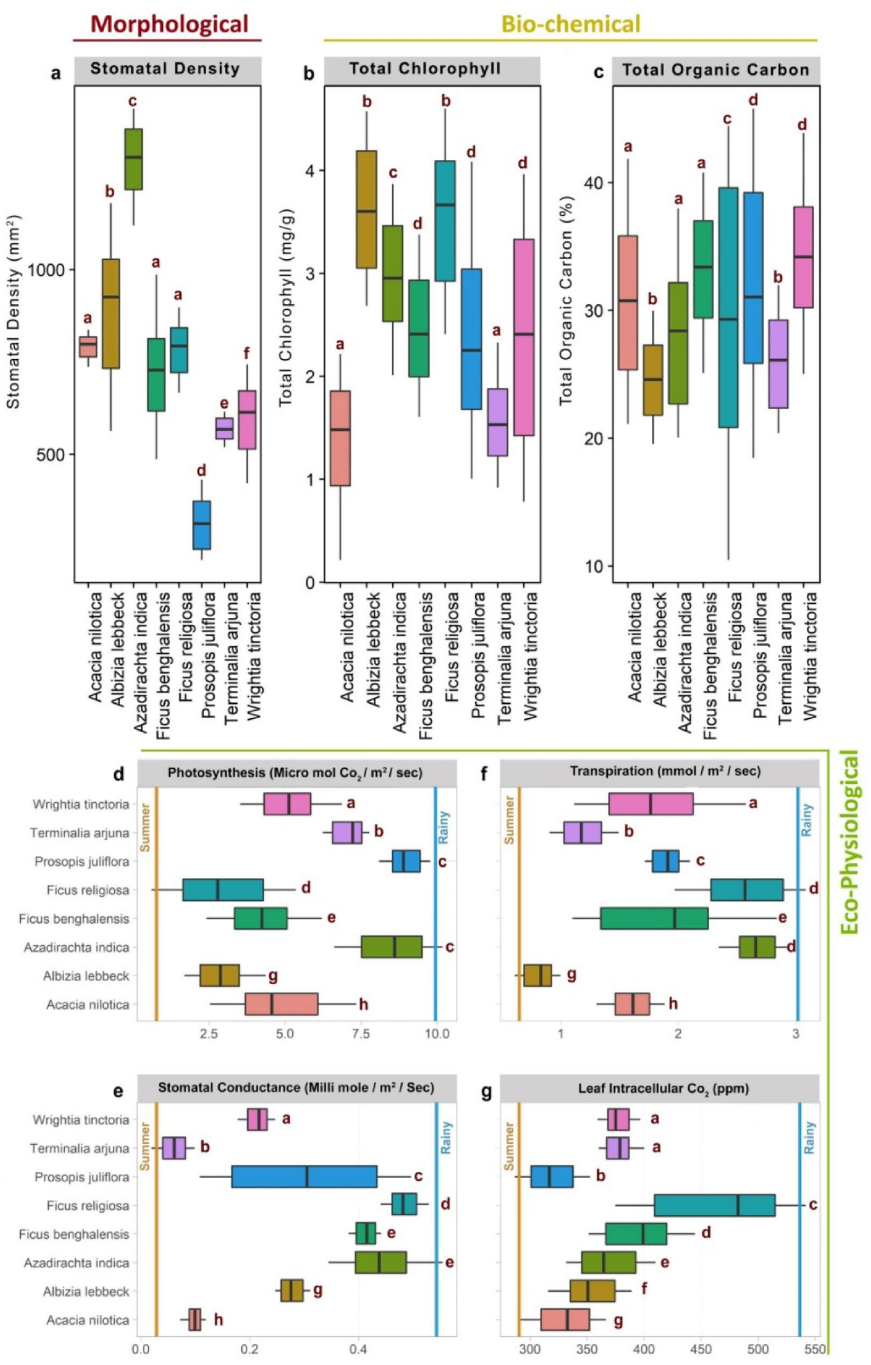
Morphological, Biochemical & Eco-physiological carbon sequestering functional trait of selected tree species in SGS and PJS across the central region of Tamil Nadu.; (**a**) Stomatal density; (**b**) Total chlorophyll; (**c**) Total organic carbon; (**d**) Photosynthesis; (**e**) Stomatal Conductance; (**f**) Transpiration; (**g**) Leaf Intracellular CO_2_ levels. Kruskal Wallis test and Pair wise comparisons using the Wilcoxon rank-sum test with continuity correction, employed to differentiate means, species sharing different letter exhibit significant differences at *p* < 0.05.

The mean adaxial and abaxial stomatal density among the tree species (selected for the experiment) showed significant variation (*p* < 0.01***—Kruskal–Wallis rank sum test) (Fig. 6a). *Azadirachta indica* exhibited the highest density (1300 ± 198 mm^2^), while *Prosopis juliflora* displayed the lowest density (287 ± 148 mm^2^) (Fig. S1).

The mean total chlorophyll levels were high in *Ficus religiosa* (3.3 ± 1.1 mg/g), while the species with the lowest chlorophyll content was *Acacia nilotica* (1.3 ± 0.87 mg/g). The higher proportion of chlorophyll in *Ficus religiosa* can be attributed to its greater demand for chlorophyll due to its larger mass and proportions (Fig. 6b). *Prosopis juliflora* exhibited the highest mean plant Total Organic Carbon (leaf, stem, bark), followed by *Wrightia tinctoria, Acacia nilotica, Prosopis juliflora, Albizia lebbeck, Azardiracta indica, Ficus religiosa, Ficus benghalensis, and Terminalia arjuna*. The highest organic carbon storage in *Wrightia tinctoria* could be attributed to its ability to fix larger proportions of organic carbon in its plant body (Fig. 6c).

The photosynthesis rates of selected tree species showed significant interspecific variation (*p* < 0.01***) in the Kruskal–Wallis rank sum test. Among the trees, *Azadirachta indica* and *Prosopis juliflora* exhibited higher photosynthesis rates, measuring 8.1 ± 2.0 µmol CO_2_/m^2^/sec and 8.0 ± 1.86 µmol CO_2_/m^2^/sec, respectively. The remaining species were ranked as follows: *Azadirachta indica* > *Prosopis juliflora* > *Terminalia arjuna* > *Wrightia tinctoria* > *Acacia nilotica* > *Ficus benghalensis* > *Albizia lebbeck* > *Ficus religiosa*. Kruskal–Wallis test and Pair wise comparisons using the Wilcoxon rank-sum test with continuity correction, exhibited significant differences at *p* < 0.05. These findings underscore the variations in photosynthesis rates among the studied tree species, providing insights into their relative photosynthetic capacities (Fig. 6d).

In the case of Stomatal Conductance*, Azadirachta indica* showed higher values (0.42 ± 0.15 millimol/m^2^ /sec), followed by *Ficus religiosa, Ficus benghalensis, Prosopis juliflora, Albizia lebbeck, Wrightia tinctoria, Acacia nilotica, and Terminalia arjuna*. Kruskal–Wallis test and Pair-wise comparisons using the Wilcoxon rank-sum test with continuity correction inferred significant differences at *p* < 0.05 (Fig. 6e).

The mean transpiration rate of tree species was found to be highest in *Ficus religiosa,* followed by *Azadirachta indica, Ficus benghalensis, Wrightia tinctoria, Prosopis juliflora, Acacia nilotica, Terminalia arjuna, and Albizia lebbeck*. Kruskal Wallis test and Pair-wise comparisons using the Wilcoxon rank-sum test with continuity correction exhibit significant differences at *p* < 0.05 (Fig. 6f).

Leaf intracellular CO_2_ was observed to be higher in *Ficus religiosa* and *Ficus benghalensis, followed by Azadirachta indica, Wrightia tinctoria, Albizia lebbeck, Terminalia arjuna, Prosopis juliflora, Acacia nilotica*. The higher intracellular CO_2_ concentration in *Ficus religiosa* and *Ficus benghalensis* can be attributed to their greater leaf thickness than the other selected tree species. Kruskal Wallis test and Pair-wise comparisons using the Wilcoxon rank-sum test with continuity correction signified differences at *p* < 0.05 (Fig. 6g).

**Figure 7:**
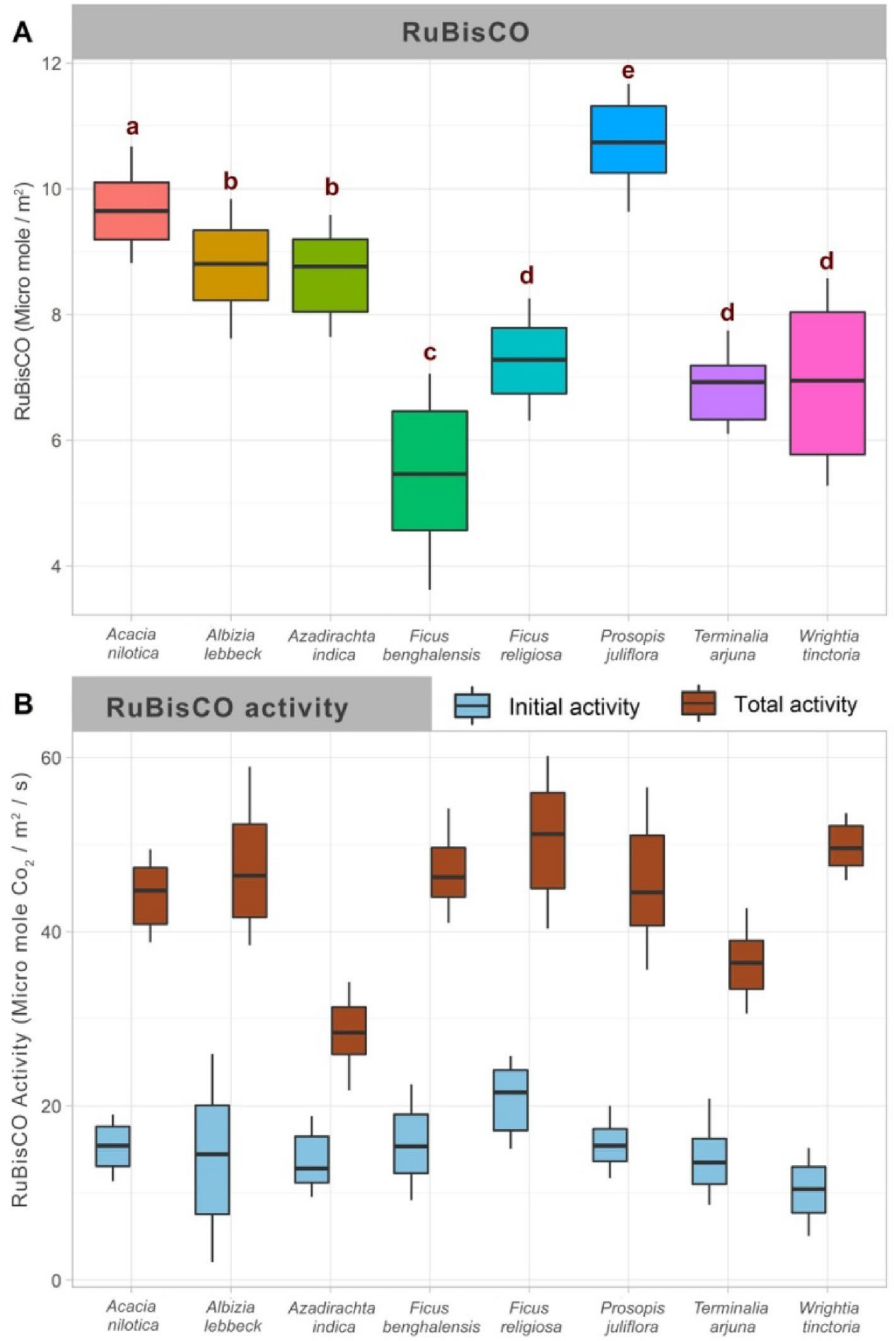
RuBisCO quantity and activity for selected tree species in Sacred Groves Stands (SGS) and *Prosopis juliflora* Stands (PJS) across the central region of Tamil Nadu. Kruskal Wallis test and Pair wise comparisons using the Wilcoxon rank-sum test with continuity correction, employed to differentiate means, species sharing different letter exhibit significant differences at *p* < 0.05.

Mean RuBisCO content in the selected eight tree species was very high in *Prosopis juliflora* (10.8 ± 1.08 micromole/m^2^) compared to other trees, implying its higher efficiency in primary productivity. RuBisCO content varied in the order: *Acacia nilotica* > *Albizia lebbeck* > *Azardiracta indica* > *Wrightia tinctoria* > *Ficus religiosa* > *Terminalia arjuna* > *Ficus benghalensis* (Fig. 7A)*.* Total RuBisCO activity across the selected species was in the following order: *Wrightia tinctoria* > *Albizia lebbeck* > *Prosopis juliflora* > *Acacia nilotica* > *Azadirachta indica* > *Ficus benghalensis* > *Ficus religiosa* > *Terminalia arjuna* (Fig. 7B).

**Figure 8:**
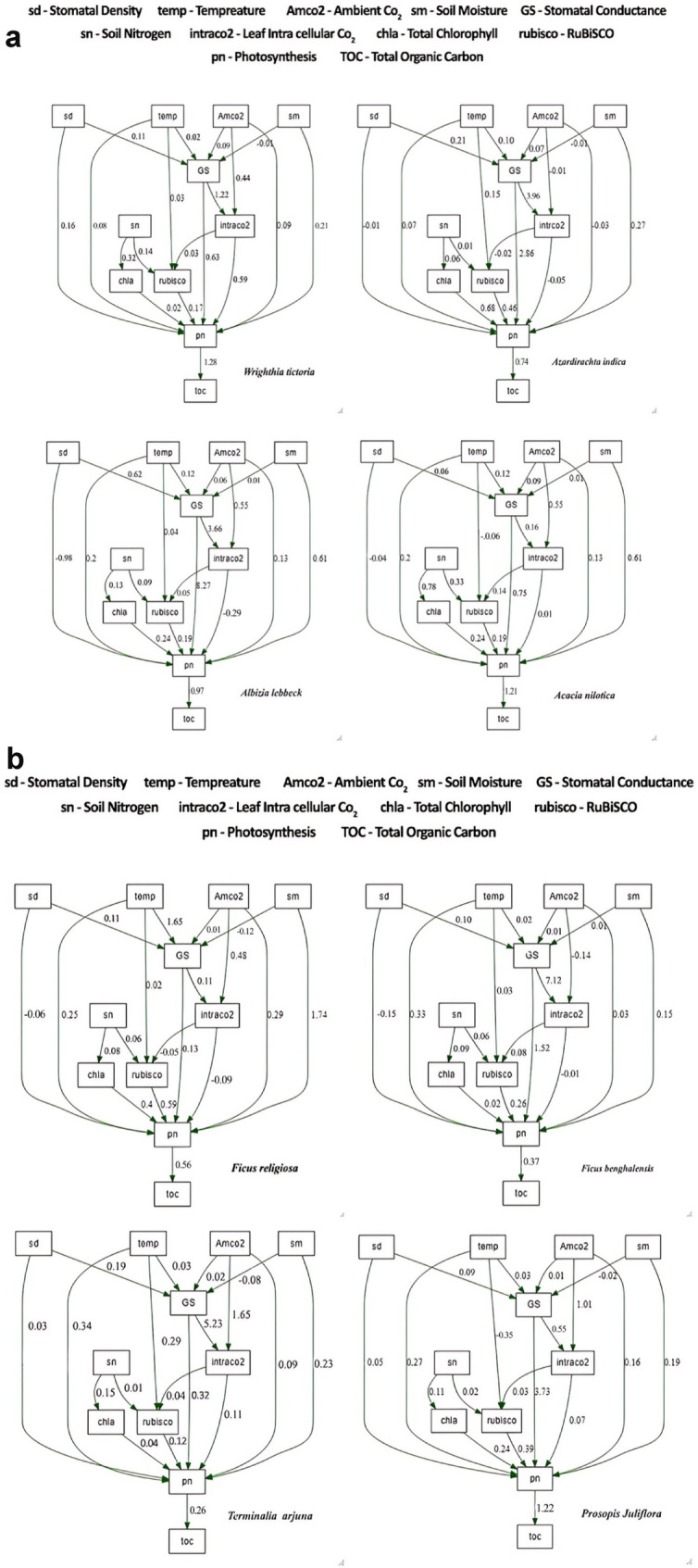
(**A**) Structural Equation Model (SEM) testing the carbon sequestering potential selected tree species involving environmental, morphological, biochemical and physiological carbon fixing functional traits. Parameters of each SEM of the respective tree species were acquired using the multiple model validation indices—lowest Akaike information criterion (AIC) value, Comparative Fit Index (CFI) > 0.95, Tucker-Lewis index (TFI) > 0.90, model test chi-square value *p* > 0.05, Root mean Square of Appropriation (RMSEA) and Standardized Root Mean Square Residuals (SRMR) < 0.08. The numbers next to the arrows in the model path diagrams denote the standardized coefficients for each path. (**B**) Structural Equation Model (SEM) testing the carbon sequestering potential selected tree species involving environmental, morphological, biochemical and physiological carbon fixing functional traits. Parameters of each SEM of the respective tree species were acquired using the multiple model validation indices—lowest Akaike information criterion (AIC) value, Comparative Fit Index (CFI) > 0.95, Tucker-Lewis index (TFI) > 0.90, model test chi-square value *p* > 0.05, Root mean Square of Appropriation (RMSEA) and Standardized Root Mean Square Residuals (SRMR) < 0.08. The numbers next to the arrows in the model path diagrams denote the standardized coefficients for each path.

The best model fit was attained for the Structural Equation Model (SEM) to test the carbon sequestrating potential of selected tree species utilizing corresponding morphological, biochemical and physiological functional traits (Fig. 8A,B). Stomatal density, ambient CO_2_, ambient temperature, available soil nitrogen and soil moisture significantly affected (positive/negative) the endogenous variable photosynthesis.

SEM-derived outputs on stomatal density's effects on tree species' photosynthesis rate showed a predominant negative relationship in species with higher stomatal density. Higher stomatal density (≥ 1000 mm^2^) showed an insignificant (*p* > 0.05) direct negative effect on the photosynthesis rate of the following species—*Azadirachta indica* (Estimated coefficient: −0.012, *p* = 0.082)*, Albizia lebbeck* (−0.09, *p* = 0.952)*.* However, in *Terminalia arjuna* (0.09, *p* = 0.003)*, Prosopis juliflora* (0.05, *p* = 0.04)*, Acacia nilotica* (0.10, *p* = 0.01)*, Wrightia tinctoria* (0.16, *p* = 0.03), *Ficus benghalensis* (0.15, *p* = 0.009)*, and Ficus religiosa* (0.16, *p* = 0.002) stomatal density < 1000 mm^2^ positively affected the photosynthetic rate significantly (*p* < 0.05) (Fig. 8A,B).

Ambient CO_2_ levels indirectly and negatively affected the optimal photosynthetic rate in the case of *Azadirachta indica* (Estimated coefficient: −0.03, *p* = 0.490)*.* However, other species exhibited indirect, neutral and positive relationships to ambient CO_2_ levels (Fig. 8A,B). Ambient temperature (32.5 ± 2.02 °C) had an overall direct positive effect on the photosynthesis rate of all the selected tree species, significance was observed for *Azadirachta indica* (0.07, *p* = 0.09) *Albizia lebbeck* (0.12, *p* = 0.004), *Ficus benghalensis* (0.03, *p* = 0.006), *Ficus religiosa* (0.28, *p* = 0.001), *Prosopis juliflora* (0.16, *p* = 0.01), *Terminalia arjuna* (0.09, *p* = 0.003)*, Wrightia tinctoria* (0.09, *p* = 0.03), *Acacia nilotica* (0.13, *p* = 0.04). The ambient temperature exhibited to have significant (*p* = 0.01) direct negative effect on the photosynthetic enzyme RuBisCO in *Prosopis juliflora* (Estimated coefficient: −0.09) and *Acacia nilotica* (−0.24, *p* = 0.04), RuBisCO in rest of the tree remain unaffected to temperature, the following trait could be attributed to the leaf thickness, as high leaf thickness lessens enzymes susceptibility to high ambient temperature. (Fig. 8A,B).

In all the eight species, a positive direct effect was observed between soil nitrogen, chlorophyll and RuBisCO. Subsequently a direct positive relation was seen between chlorophyll, RuBisCO and photosynthesis in all the eight species. In all these species, soil moisture had a positive effect on photosynthesis; higher significance was found in *Albizia lebbeck* (*p* = 0.01), *Ficus benghalensis* (*p* = 0.001), *Ficus religiosa* (*p* = 0.001), *Prosopis juliflora* (*p* = 0.001), *Terminalia arjuna* (0.09, *p* = 0.003)*, Wrightia tinctoria* (0.09, *p* = 0.03), *Acacia nilotica* (0.13, *p* = 0.04), *Azadirachta indica* (*p* = 0.005). Our findings advocate that the high carbon fixing efficiency of *Albizia lebbeck, Prosopis juliflora, Wrightia tinctoria, and Acacia nilotica* can be attributed to the general trait of the Fabaceae family, which enables them to fix nitrogen to the soil, and soil nitrogen is explained to have an indirect positive relation with photosynthesis. Furthermore, it is validated that nitrogen availability plays a crucial role in supporting essential biosynthetic metabolic processes. SEM elucidated the carbon sequestering potential of selected tree species depicted in the following hierarchy: *Wrightia tinctoria* (estimated coefficient: 1.28, *p* = 0.02) > *Prosopis juliflora* (1.22, *p* = 0.01) > *Acacia nilotica* (1.21, *p* = 0.03) > *Albizia lebbeck* (0.97, *p* = 0.01) > *Azadirachta indica* (0.74, *p* = 0.02) > *Ficus religiosa* (0.56, *p* = 0.08) > *Ficus benghalensis* (0.37, *p* = 0.002) > *Terminalia arjuna* (0.26, *p* = 0.001) (Fig. 8A,B). SEM outputs revealed that Fabaceae family members have higher carbon sequestration potential—*Wrightia tinctoria* (estimated coefficient: 1.28, *p* = 0.02) > *Prosopis juliflora* (1.22, *p* = 0.01) > *Acacia nilotica* (1.21, *p* = 0.03) > *Albizia lebbeck* (0.97, *p* = 0.01) compared to other families members Meliaceae—*Azadirachta indica* (0.74, *p* = 0.02); Moraceae—*Ficus religiosa* (0.56, *p* = 0.08), *Ficus benghalensis* (0.37, *p* = 0.002); Combretaceae—*Terminalia arjuna* (0.26, *p* = 0.001) (Fig. 8A,B).

Effects of endogenous physiological variables such as RuBisCO, Chlorophyll, and intracellular CO_2_ on the photosynthesis rate of eight selected tree species did not exhibit uniformity. All three endogenous variables exhibited a positive direct effect on photosynthesis in the following tree species: *Prosopis juliflora* (Estimated coefficients: 0.39, 0.24, 0.07)*, Acacia nilotica* (0.39, 0.24, 0.07)*, Albizia lebbeck, Wrightia tinctoria, Terminalia arjuna* (0.04, 0.12, 0.11). However, in the case of *Azadirachta indica* (−0.05, *p* = 0.04)*, Ficus benghalensis* (−0.01, *p* = 0.04*) and Ficus religiosa* (−0.09, *p* < 0.01), leaf intracellular CO_2_ tend to have a direct negative effect on optimal photosynthesis very significantly. RuBisCO (*Azadirachta indica*: 0.46; *Ficus benghalensis*: 0.26; *Ficus religiosa*: 0.59) and chlorophyll (*Azadirachta indica*: 0.68; *Ficus benghalensis*: 0.02; *Ficus religiosa*: 0.40) is observed to have direct positive relation (Fig. 8A,B). A negative relationship between temperature and RuBisCO was observed in *Prosopis juliflora and Acacia nilotica.* However, the temperature did not affect RuBisCO in *Ficus religiosa*, *Ficus benghalensis*, *Azardiracta indica, Albizia lebbeck, Wrightia tinctoria*, and *Terminalia arjuna*. This trait could be attributed to the leaf thickness, as the leaf thickness lessens enzymes susceptibility to high ambient temperature.

## Discussions

Field expeditions and analytical assessments conducted to examine the floral ecology of SGS and PJS revealed distinct levels of floral diversity. SGS exhibited high floral diversity and Shannon wiener index compared to PJS, which has low floral diversity, Shannon wiener index. Despite these differences, our study hints that both these vegetation types offer considerable regulating ecosystem services. Previous research highlighted the pivotal role of biodiversity in governing and regulating ecosystem services in forest ecosystems^51^. Wherever the vegetation and the canopy are dense, such as sacred grove-like forest fragments rich in biodiversity, it is associated with substantial carbon stocks^30^, nutrient-rich soils^51^, better water quality^52^, higher groundwater availability^53^, pathogen resistance^54^, and resistance to exotic taxa^55^. And, once the floral diversity and species richness decline, it has negative implications on the ecosystem functions and eventually on the ecosystem services^56^. Greater species richness is also linked to remarkable genetic diversity, fostering ecosystem resilience against various biotic and abiotic factors, exemplifying climate-resilient forests^57^. Diverse ecosystems hold a variety of species populations with diverse functional traits contributing to sustainable ecosystem services throughout their lifespans^51^. Where as in the *Prosopis juliflora* dominated vegetative stands, earlier studies elsewhere had documented monotonous ecosystem services^58–60^. Although PJS demonstrates high carbon stocks and nutrient-rich soils in the arid and semi-arid regions, it draws flak from the public due to groundwater depletion^61^. The current study noted the loss of floral diversity due to the invasion of *Prosopis juliflora*, reinforcing the detrimental impact of this invasive species on ecosystem composition. Future reforestation and ecological restoration of invasive vegetation must focus on selecting appropriate native trees that could potentially overcome the ill effects induced on the landscape by invasive taxa.

Regarding nutrient enrichment, current research demonstrates that PJS exhibit higher soil nutrient proportions than SGS. Higher soil nutrient in PJS could be associated with the higher abundance of Fabaceae family members. Notably, within the PJS stand, the representation of the Fabaceae family members was quantified as 364 trees per hectare (Fig. 1d), in contrast SGS had 74 Fabaceae members per hectare (Fig. 1c). The plant species of the family Fabaceae are known to have symbiotic associations between their nodulated roots and an array of rhizobacteria^62,63^. In line with this phenomenon, plants in the study sites like *Prosopis juliflora*, *Albizia lebbeck, Wrightia tinctoria*, etc., facilitate the nitrogen fixation process, enriching the soil with biologically available nitrogen. Consequently, this nutrient enrichment stimulates other nutrients’ cycling, further increasing the invasion rate within the ecosystem^64^. Earlier studies on this aspect have validated such occurrences^49,65^ for instance, the levels of available nitrogen increased from 0.75 to 1.2 g/kg, while organic carbon content rose from 0.2 g/100 g to 1 g/100 g from 1981 to 1989 in the experimental plots of *Prosopis juliflora*. The dynamics include direct fixation of nutrients, deposition of organic matter through litter fall, promotion of root exudation and aeration in the root zone, which facilitates the activity of mutualistic aerobic microorganisms, consequently enabling nutrient cycling and enhancing the overall soil nutrient pool^66^. Nutrient-enriched soil acts as a conducive substrate for the germinating seeds/saplings, taxa that don’t have the functional trait to generate nutrient islands beneath its canopy, and source its nutrients from others with potential functional traits to fix nutrients^62^. The outcomes of this study suggest that transforming PJS to forest groves using apt native tree species could be a viable alternative despite certain challenges. The major challenge confronting such an exercise could be the recalcitrant nature of the soil, perhaps due to the presence of allelochemicals (L-tryptophan, 3-oxo-juliprosine) released by the *P.juliflora* ^67^. The native trees deemed to coexist or can outcompete invasive species in those landscapes can potentially be used for restoration. Nevertheless, the nutrient-rich soil, as observed in the present study, could bolster the transformation and restoration of native vegetation.

Microscopic evaluation from the present investigation revealed that the stomatal density of tree species varied significantly between the selected species. An earlier study hints that individual gene expression (STOMAGEN) and genetic imprint can significantly affect the variations of stomatal densities in the tree species^68^. In the present investigations, photosynthesis, transpiration, stomatal conductance, leaf intracellular CO_2_, total chlorophyll, total organic carbon and RuBisCO significantly varied among the selected species, which could also be attributed to the specific genetic imprints^69–73^. In an attempt to unravel the ill-famed ground water-depleting trait of *Prosopis juliflora,* the present study compared the transpiration rate in a communal scale between SGS and PJS, and surprisingly, *Prosopis juliflora* recorded only a medial position. Therefore, it may be interpreted that despite moderate transpiration in *Prosopis juliflora*, the large leaf area and dense canopy could be linked to groundwater depletion. This is in conjunction with other studies in arid and semiarid regions of Ethiopia that report that a mature *Prosopis juliflora* tree consumes 4.74 ± 1.97 L/day, while *Senegalia senegala* (a native tree) consumes 6.46 ± 3.7 L/day. It also ascertains that *Prosopis juliflora* coppices at the community scale with stand density of 1200–1600 trees consume 5688–7584 L/day/ha, whereas *Senegalia Senegal* (stand density: 400–600 trees) consumed 2584–3876 L/day/ha^74,75^*.* Furthermore, the evergreen nature of *Prosopis juliflora,* despite the region's aridity, ostensibly necessitates high transpiration and water consumption, consequently depleting ground water^74,76^. On the other hand, most of the native trees in the arid and semiarid regions are deciduous; transpiration per unit area is seasonally variable and, overall, comparatively lower.

The current findings show that the above-ground biomass (AGB) and below-ground biomass (BGB) was high in PJS (AGB = 56 ± 35 tons/ha; BGB = 16.2 ± 10.3 tons/ha) compared to SGS (AGB = 45 ± 31 tons/ha; BGB = 9.6 ± 8.8 tons/ha). Measurements from the current study with respect to the basal area in PJS (38 ± 18 m^2^ /ha) and SGS (25 ± 11 m^2^ /ha) validate the differences mentioned above in AGB and BGB between PJS and SGS. Several studies have pointed out that AGB and BGB are attributed to the existing vegetation's high basal area, carbon fixing potential and robust growth of the tree species^77–82^. The current study found carbon stock in PJS and SGS to be 32 ± 20.6 tons/ha and 27.3 ± 22.4 tons/ha, respectively. The findings of the current study are in line with a recent study^83^ on deciduous forests (denoted as Sacred Groves Stands—Outside the protected area, Pachaimalai tropical hill forest) in central Tamil Nadu in which mean carbon stock ranged between 7.6 t ha^-1^ to 23.4 t ha^-1^. While in the case of *Prosopis juliflora* stands, no authenticated information on carbon stock in India is reported, but a study in Ethiopia recorded it to be in the range from 85.8 ± 4.7 Mg/ha (in dense thickets) to 48.2 ± 4.4 Mg/ha (in sparse thickets)^84^.

The present study revealed lower Photosynthetic Potential/Gross Primary Productivity of SGS compared to PJS. The present study also articulated higher Gross Primary Productivity/ Photosynthesis of species belonging to Fabaceae, Capparaceae, Moraceae, and Meliaceae, contributing to the Net Ecosystem Production (NEP). The Gross Primary Productivity (GPP) of vegetation stands are determined by the individual photosynthetic potential of tree species, genetic imprint and relative abundance^36^. The variation in carbon sequestration is also likely influenced by temperature, precipitation, atmospheric pressure, solar light availability and elevation^85^. Concerning NEP, the current study recognized that PJS and SGS are potential carbon sinks during the young stage. As the transition from young to mature occurred, SGS acted like a sink while the PJS remained a source. However, as PJS proceeds to the old-growth phase, it becomes a potential CO_2_ source; but in the case of SGS, even after the transition from mature to the old-growth phase, the growth of young saplings allowed the system to act partially as a CO_2_ sink. The phenomenon of younger vegetation serving as a carbon sink and older vegetation as a carbon source is presumably universal^86^. A recent report from Amazon tropical forest also ascertains that as the trees undergo ageing processes, during decay and decomposition, the organic matter in older vegetation breaks down, and consequently release a bulk volume of carbon dioxide (CO_2_) back into the atmosphere^87^. In the present study, soil respiration in older stands was higher in PJS than in SGS. Soil respiration increases with soil microbial load: an ecological study recorded 350 ± 121 /g microbial strains under the canopy soil of dense *Prosopis juliflora* stands^88^ in the case of SGS, microbial diversity was recorded as 134 ± 87 /g^89^. Higher proportions of root exudates and organic matter in the soil stimulate the growth of soil microbiota, and a higher microbial population emits higher proportions of CO_2_ into the atmosphere via soil respiration.

In the current investigation, PJS demonstrated notably reduced levels of soil erosion compared to SGS. Subsequent analysis of soil nutrient composition within the present study indicated that the diminished soil erosion observed in PJS could be ascribed to its elevated soil organic carbon content. Consequently, these findings substantiate the postulation put forth in a prior study that delved into the pivotal functions of soil organic carbon within the rhizosphere of plants, particularly within the substantial reservoirs of soil. Furthermore, the study posited that carbon-rich root exudates betine and other polysaccharides, which function as adhesive agents, effectively stabilizing the soil and mitigating soil erosion. Many earlier studies had ascertained the efficacy of this adhesive relationship in reducing soil erodibility^90–94^. A recent study in the Dianchi watershed region of China substantiated the relationship, in which it was observed that young forests with low soil organic carbon exhibited an annual soil erosion rate of 11.02 ± 4.77 tons per hectare. In contrast, mature forests with high soil organic carbon exhibited a rate of 3.34 ± 1.88 tons per hectare^95^. Similarly, in India, research conducted in the forest landscape in the Western Ghats region reported a controlled annual soil erosion rate of 68.26 tons per hectare with vegetation, while in open lands, soil erosion rate was 89.36 tons per hectare^96^.

According to the SEM Model output, the morphological, biochemical, and physiological functional traits play a pivotal role in determining the photosynthetic efficiency of different tree species. In the current study, stomatal density varied in the order: *Azadirachta indica* > *Albizia lebbeck* > *Ficus religiosa* > *Acacia nilotica* > *Ficus benghalensis* > *Wrightia tinctoria* > *Terminalia arjuna* > *Prosopis juliflora*. While relating the stomatal density to the photosynthetic efficiency, water availability is an important parameter to reckon with in the semi-arid regions, and conditions such as stomatal density are inversely proportional to photosynthetic efficiency. This phenomenon was previously explained by experimental studies in controlled conditions (heat/drought), where plants with high stomatal density tend to lose more water, which triggers stomata closing, eventually decreasing the photosynthesis rate^97,98^. Even moderate water stress can limit photosynthesis by stomatal resistance (closure) and carboxylation inhibition^99,100^. Furthermore, when plants are exposed to water stress, they produce ABA (Abscisic acid) that signals the stomatal closure to control water loss^101,102^. This phenomenon of higher stomatal density > 1000 mm^2^ implicates the lower photosynthetic rate in *Azadirachta indica* during water stress conditions in summer, while *Prosopis juliflora* showed comparatively higher photosynthetic rate in the present study.

Our field observations also indicated that ambient CO_2_ level fluctuations (398 ± 12 ppm) directly correlated with atmospheric temperature, affecting the photosynthesis rate. In response to higher ambient temperature, stomata get closed to control the water loss, and consequently, CO_2_ intake via stomatal conductance is reduced, diminishing the photosynthesis rate of tree species^103–108^. As the impacts of climate change are becoming more tangible in India, extreme summer and prolonged drought stress could potentially affect the photosynthetic rate of native tree species. Studies also speculate certain native tree species can withstand a shorter period of stress; however, prolonged and extended summers could alter the photosynthesis while the trees tend to set forth their energy for survival rather than increasing productivity levels^109–112^. In that context, the outcomes of this study reveal native trees like *Acacia nilotica, Terminalia arjuna, Wrightia tinctoria,* and *Ficus benghalensis* are likely to perform and thrive well in the ensuing climate change scenarios in the semi-arid regions of India. This study also demonstrated that an increase/decrease in other exogenous parameters, such as soil moisture, intracellular CO_2_, chlorophyll, RuBisCO, leaf water potential, stomatal conductance, and ambient temperature, tends to regulate photosynthesis rate in a positive/negative manner similar to earlier observations^107,113–119^. Particularly, the present study observed the sensitivity of RuBisCO activity to heat, especially for the species with lower leaf thickness. Earlier studies infer that high temperature limits photosynthesis by ceasing the activity of the enzyme RuBisCOactivase^117,120,121^; however, the activity is reversed upon cooling^122^. This study also found a positive relationship between soil nitrogen availability and photosynthesis rate, and it was predominantly high in the taxa that had the potential to fix nitrogen in the soil, also substantiated by earlier investigations^123–126^. Decisively, the current investigation leads to the conclusion that the high carbon-fixing potential of tree species is intricately linked to the presence of distinct adaptive functional traits, encompassing stomatal density below 1000 mm^2^, nitrogen-fixing capability, intracellular CO_2_the presence of chlorophyll, and the content of the RuBisCO enzyme. The following species belongs to the family Fabaceae (*Prosopis juliflora, Wrightia tinctoria, Acacia nilotica,* and *Albizia lebbeck)* exhibited higher productivity levels due to the aforementioned functional traits.

Future research can focus on the significance of adaptive capacities of forest sacred groves with emphasis to native trees and shrubs as well. Furthermore, the significance of agro climatic and microclimatic variations can provide deeper insights on region specific management of sacred forest groves. *Prosopis juliflora* coppices are least studied in terms of pollination and other ecological indices; future research can also focus on ecological restoration at PJS rather than complete removal. Given the eradication drive of *Prosopis Julifora* in India and elsewhere across the world, careful ecological consideration is warranted in ecologically sensitive zones (such as wetlands, forests, and biosphere reserves). Vegetative remediation using selected native tree species (recorded by the current study) may help to ecologically restore the invasive landscapes. In the arid and semi-arid regions of India, characterized by extensive dry landscapes, the proliferation of *Prosopis juliflora* is of substantial magnitude. The findings of this study hold the potential to provide insights for restoration ecologists, land managers, and policymakers engaged in formulating strategies for ecological restoration.

## Conclusion

*Prosopis juliflora* Stands (PJS) and Sacred Groves Stands (SGS) have demonstrated significant disparities in floral diversity, soil nutrient content, and soil erosion rates. SGS exhibited elevated functional diversity with low functional evenness, while PJS displayed low functional diversity and high functional evenness. Specific functional traits, such as species richness and photosynthesis rates, varied distinctly between the two ecosystems, with SGS recording high species richness and low photosynthesis, and PJS exhibiting low species richness and high photosynthesis rates. This indicates the potential carbon sequestration capacity of *Prosopis juliflora* Stands. But regarding carbon dynamics and long term retention of carbon in terrestrial ecosystems, the study found that *Prosopis juliflora* Stands (PJS) upon reaching the old-growth phase, they act as potential CO_2_ sources. Conversely, in Sacred Groves Stands (SGS), even after the transition from mature to old-growth phases the system to function partially as a CO_2_ sink, could be attributed to the growth of young saplings, where in PJS *Prosopis juliflora* inhibits the growth of other plant species. Structural Equation Modeling (SEM) outputs unveiled. Ambient CO_2_ levels had an indirect and negative effect on the optimal photosynthetic rate in the case of *Azadirachta indica* (Estimated coefficient: -0.03) *and Albizia lebbeck* (Estimated coefficient: -0.01). A negative relationship between temperature and RuBisCO was observed in *Prosopis juliflora and Acacia nilotica, while* no notable effects was observed in *Ficus religiosa, Ficus benghalensis, Azardirachta indica, Albizia lebbeck, Wrightia tinctoria, and Terminalia arjuna*. Carbon sequestering potential of selected tree species had following hierarchy: *Wrightia tinctoria* (estimated coefficient: 1.28) > *Prosopis juliflora* (1.22) > *Acacia nilotica* (1.21) > *Albizia lebbeck* (0.97) > *Azadirachta indica* (0.74) > *Ficus religiosa* (0.56) > *Ficus benghalensis* (0.37) > *Terminalia arjuna* (0.26). *Prosopis juliflora*, *Albizia lebbeck, Wrightia tinctoria* predominantly facilitates the process of nitrogen fixation, resulting in the enrichment of soil with biologically available nitrogen. The high carbon sequestration potential of the Fabaceae family compared to other families could be attributed to the specific carbon sequestering functional traits such as stomatal density below 1000 mm^2^, nitrogen-fixing capability, chlorophyll presence, and the content of the RuBisCO enzyme. Particularly, the study recommends the utilization of native Fabaceae members for ecological restoration initiatives in the arid and semi-arid zones of Southern India and similar landscapes elsewhere.

## Materials and methods

### Study area and sampling sites

**Figure 9:**
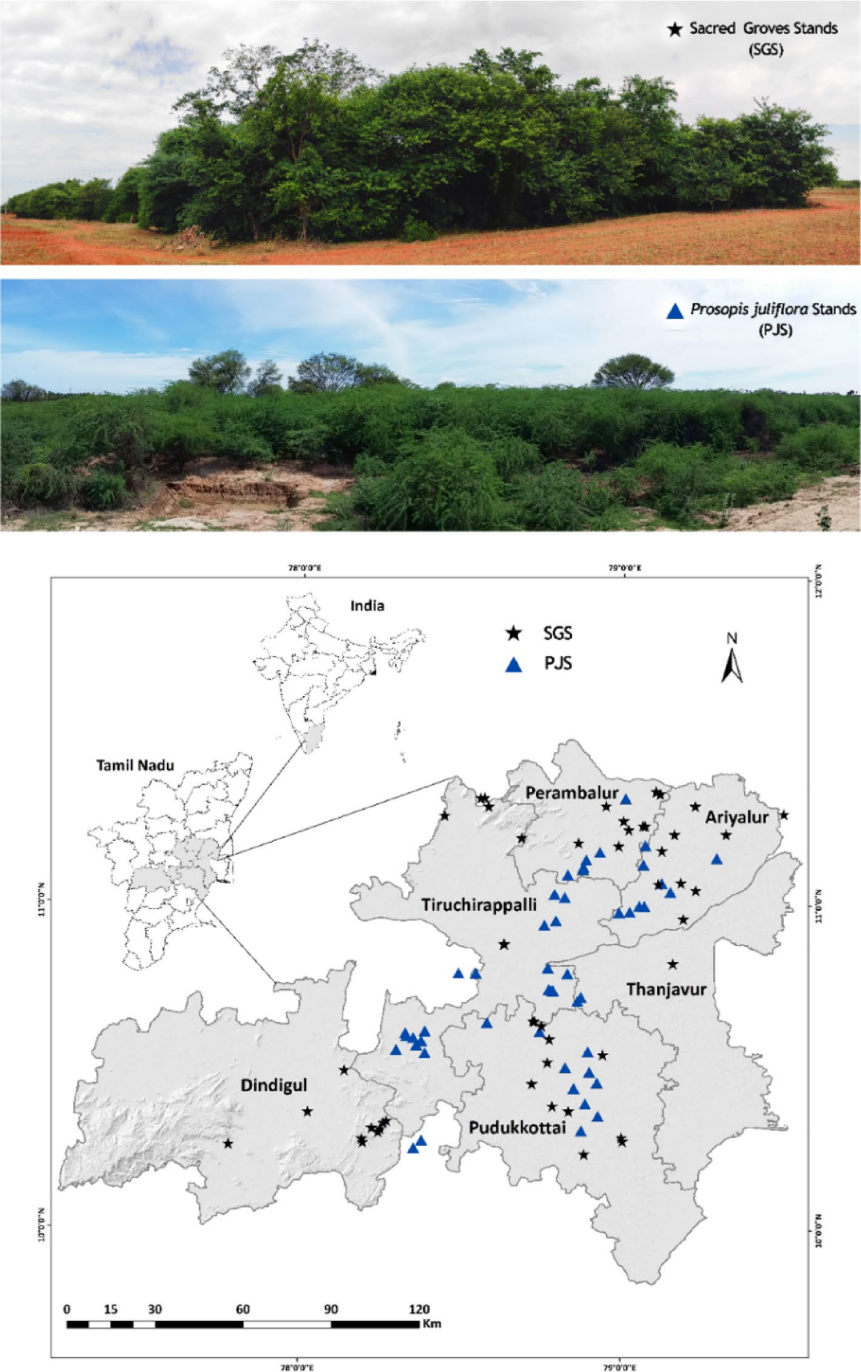
Study sites—Sacred Groves Stands (SGS) and *Prosopis juliflora* Stands (PJS) across the central region of Tamil Nadu., India.

**Figure 10:**
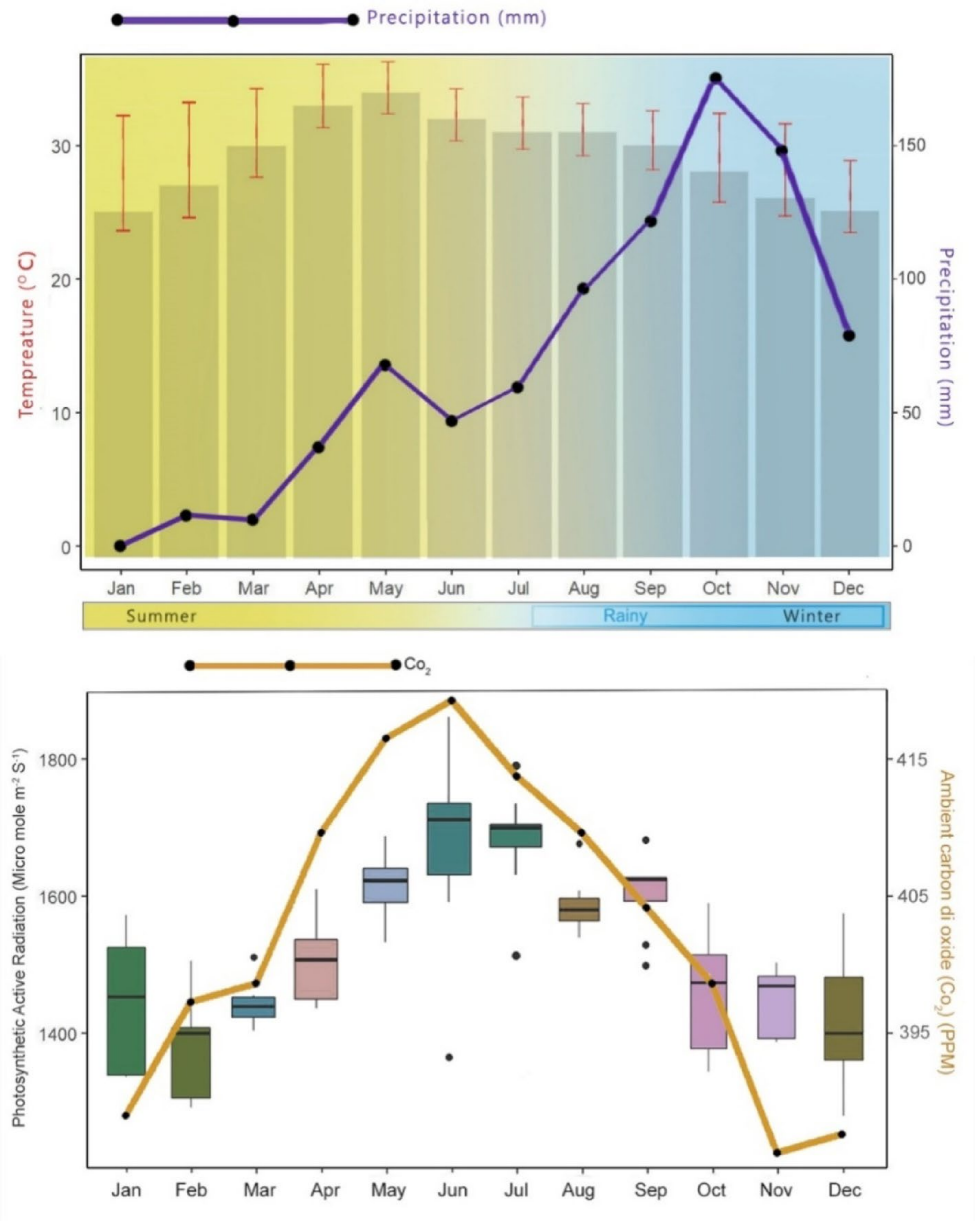
Ambient temperature and precipitation during the year 2022 across the central region of Tamil Nadu. (**a**) Observed ambient temperature (°C) and mean precipitation levels (mm). (**b**) Photosynthetic Active radiation (µ mole m^-2^ s^-1^) and mean ambient Carbon dioxide (ppm).

Fifty Sacred Grove Stands (SGS) and another fifty sites representing invasive *Prosopis juliflora* stands (PJS) were chosen in a typical semi-arid region (Tables S1 and S2). These study sites lie in the central districts (Tiruchirappalli, Pudukkottai, Dindigul, Ariyalur, Thanjavur and Perambalur) of Tamil Nadu, India, spreading across 14,371 km^2^ (10^◦^ 19′ to 11^◦^ 26′ North latitudes and 77^◦^ 30′ to 79^◦^ 19′ E (Fig. 9). A recent study currently monitoring the Pan-India invasive species, reports that *Prosopis juliflora* had invaded 21,332 ha in Tiruchirappalli, 31,422 ha in Pudukkottai, 32,397 ha in Dindigul, 7,987 ha in Ariyalur, 20,194 ha in Thanjavur and 17,842 ha in Perambalur^127^. A portion of the study area falls under the Cauvery River Deltaic Region and the quaternary sediments are of fluvial type^128^. The annual mean temperature of the study districts ranges between 27.5 and 36.0 °C, and the annual precipitation ranges from 286 to 880 mm (Fig. 10). At each site, soil physicochemical parameters, vegetation analysis, including species richness and stand density, and other parameters representing the regulatory ecosystem services were estimated.

### Communal functional trait assessment of SGS and PJS

#### Ecological survey

In all the 100 sites, four quadrates of dimensions 25 × 25 m were laid for tree survey and parameters such as stand density, species richness, abundance, basal area and leaf area index were recorded. Characteristics such as the Shannon diversity index, Simpson dominance index, and Pielou’s evenness index were derived using R package #vegan. Litter fall in all the sites was recorded using typical rectangular baskets of dimensions 51 × 50 cm; all the accumulated litter were collected and weighed^129^. The Leaf Area Index was estimated using the Digital Hemispherical photographic technique; a digital camera (NIKON 7700D) with fisheye lens (Sigma EX-DC 4.5 mm) was erected vertically 1.5 m above the ground towards the forest canopy to capture canopy pictures. To classify the threshold between sky and canopy data, preprocessing was done using *Sidelook* software v1.1.01; subsequently, LAI was calculated using the software *Gap Light Analyzer*^103^. Such that in each location (SGS—50, PJS—50) three hemispherical pictures were taken to arrive a successive mean.

#### Soil nutrient analysis

In all the 100 sites [50—Sacred Groves Stands (SGS), 50—*Prosopis juliflora* Stands (PJS)], composite soil samples were collected at depths 0–30 cm for nutrient profiling (N, P, K, OC), bulk density, pH and electrical conductivity. Soil samples were dried in shade, sieved, and then stored in sterile containers under dark conditions for future analysis. Standard methodologies were adopted to analyze soil pH, electrical conductivity, bulk density^130^, organic carbon^131^, available nitrogen content^132^, available phosphorus content^133^, and available potassium content^134^.

#### Carbon stock assessment

Above and Below Ground Biomass (AGB and BGB) of SGS & PJS were estimated based on the field measurements 1) tree height, 2) diameter at breast height (DBH), and 3) wood density (wood density was acquired from the existing wood density database in the R package BIOMASS) (Table S6); AGB was estimated using general allometric Eq. 4^135^ in R package #BIOMASS. Furthermore, to assess the total carbon content of the selected tree species, stem, leaf, and bark samples from each species were collected and total carbon content was quantified under laboratory conditions^136^ following the formula: Total carbon (%) = 100—(Ash weight) + Molecular weight of O_2_ (53.3) in C_6_ H_12_ O_6._

### Carbon dynamics

#### Plant respiration

Leaf nocturnal dark respiration measurements for 50 tree species of SGS and PJS were carried out continuously for 24 h in 3-day intervals^137,138^. The rates of leaf dark respiration, i.e. CO_2_ efflux, were quantified for the sampled leaves in triplicates using a Plant Photosynthesis meter (Bio Base—BK 3051 C—standard leaf chamber area: 11 cm^2^); airflow rate and standard reference CO_2_ was set at 400 μmol mol^−1^ and 380 μmol mol^−1^ respectively^139^. Ambient CO_2_ levels were recorded using Plant Photosynthesis meter.

#### Soil respiration

Soil Respiration was measured with a stratified random sampling design (based on tree age as criteria for stratification) in each land-use type to account for the spatial variability in soil properties and vegetation cover. A closed soil chamber of 962 cm^3^ in volume and 72 cm^2^ in area was used for soil respiration measurements; PVC chamber mount collars were inserted into the soil prior (24 h); the measurement surface was kept free from autotrophic respiration by removing herbs and seedlings. Then, the soil chamber was mounted on the PVC collar and locked. Before starting the experiment, the initial concentration of CO_2_ in the respiration chamber was measured. Then, the increase in soil respiration under closed conditions was measured using (IRGA) CO_2_ sensor (GasAlertMicroIR5, BW Tech., Honeywell Inc., Charlotte, NC, USA). To avoid any high midday temperature, measurements were performed from 09.00 am to 11.00 am^140^.

### Dendrochronological assessment

The age of the tree was measured using incremental borer technique^141^. Trees were bored twice, from the trunk's northern and eastern sides. The bore was set at a right angle to the trunk. The drilling depth was equal to half of the DBH, with a margin of 5 to 10 cm, except for trees with a diameter of more than 70 cm, where the length of the boring bit limited the depth of the drilled hole. Based on the incremental borer survey data stepwise linear regression model was fitted. Another field survey was conducted in 25 timber mills in the study region to increase the regression model's accuracy. Before boring, the trees selected for boring were subjected to vascular ring count, DBH and circumference measurements. Thus, in total, 800 measurements were recorded. To construct a precise predication regression model, R packages #mass and #Random Forest were used (results presented in Fig. S5).

#### Soil erosion

**Figure 11:**
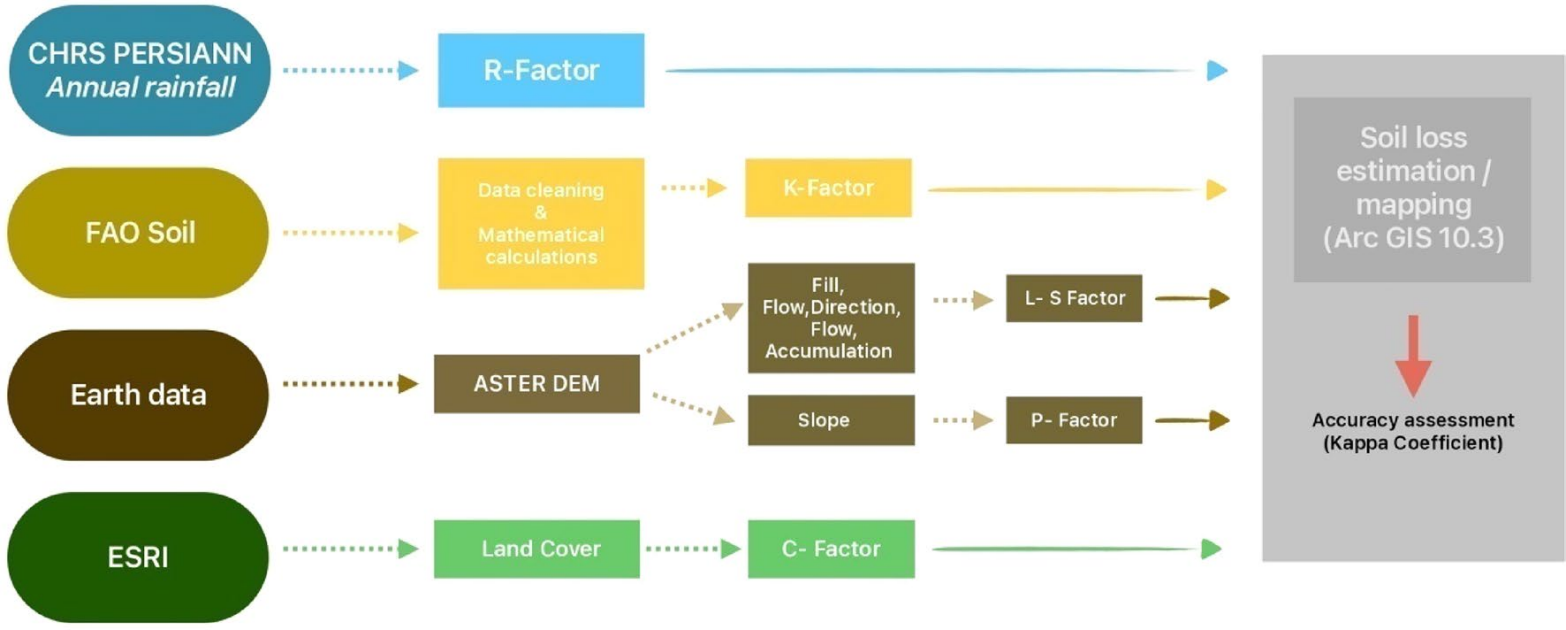
RUSLE soil erosion mapping methodology, RUSLE—model validation attained using Kappa Coefficient with threshold limit > 90%.

The revised Universal Soil Loss Equation (RUSLE) model, in conjunction with ArcGIS Pro and ArcGIS 10.3, was adopted to map soil loss. Five factors were considered for soil loss estimation: rainfall erosivity (Fig. S7), soil erodibility (Fig. S8), slope steepness and length (Fig. S9), crop management factor (Fig. S11), and support practice for the RUSLE model (Fig. S10). Datasets were prepared at varying resolutions (Fig. 11). The factors varied spatially and temporally and were interdependent with other factors. Aster DEM at 30 m resolution and ESRI land cover data were used to compute the factors in ArcGIS Pro and ArcGIS 10.3. This method is based on quantitative analysis using the analytical tools of ArcGIS to assess the statistical properties of rainfall, soil erosion data, and digital elevation model ^142^. The Kappa coefficient was used to validate the constructed RUSLE model for soil erosion (Table S15). All the above mentioned communal attributes are subjected for functional diversity and evenness estimation in SGS and PJS using the R package – fundiveristy.

### Measuring carbon sequestration potential of selected tree species through structural equation modeling by integrating morphological, biochemical, and eco-physiological functional traits

#### Morphological characterizations

Based on the preliminary study on stand density of trees, rank abundance, and Photosynthesis, seven native trees from SGS and one invasive tree (*Prosopis Juliflora*) from PJS, were selected for further investigation on carbon fixing functional traits assessment. Morphological trait, i.e. stomatal density in the adaxial and abaxial sides of the leaf, was determined using the leaf imprints method. Clear nail polish was applied on the adaxial and abaxial sides of the mature leaf from the selected eight tree species; later dry nail polish layer was peeled off using clear tape and pasted on a clear sterile microscopic slide. Mounted specimens are observed under a light microscope at 40 × magnification using the software Image View; microscopic images of the stomata were captured using APTINA trinocular camera^143^.

#### Eco-physiological characterization

Photosynthesis Rate (µmole CO_2_ m^2^/sec), Air temperature (°C), Leaf intracellular CO_2_ Concentration (ppm), Ambient Photosynthetic Active Radiation (µmole m^2^/sec), Transpiration rate (m. mole H_2_O m^2^/sec) were determined for the 51 tree species existed in SGS and PJS using Plant Photosynthesis system (Bio Base—BK 3051 C). All the above parameters were measured in the tree's top, middle, and low canopy leaves in triplicates to arrive at a successive mean.

#### Biochemical characterization

Total chlorophyll content in leaves was determined following a methodology established by Arnon^144^. Fresh leaf samples (0.5 g) were ground with 10 ml of 80% acetone, centrifuged at 3000 rpm for 10 min, and the supernatant containing the chlorophyll extract was collected for spectrometric analysis. Absorbance measurements were carried out at three specific wavelengths, namely 663 nm, 645 nm, and 470 nm, using a UV spectrophotometer. Arnon's equation was followed to calculate the amounts of chlorophyll a, chlorophyll b, and total chlorophyll.

Ribulose-1,5-bisphosphate carboxylase oxygenase (RuBisCO) quantity and activity were determined following the methods described by Fraquhar and Lilley ^145,146^. Fresh leaf samples of selected trees of weight 0.5 g were homogenized in a pre-chilled mortar using an ice-cold extraction buffer solution. The extraction buffer solution consisted of 50 mM Tris–HCl (pH 7.5), 1 mM ethylene diamine tetra acetic acid (EDTA), 1 mM magnesium chloride (MgCl2), 12.5% (v/v) glycerin, 10% poly vinyl pyrrolidone (PVP), and 10 mM mercaptoethanol. Homogenized samples were filtered through four layers of Mira cloth and centrifuged at 10,000 rpm for 10 min at 4 °C, and the supernatant was used for the enzyme assay. The initial RuBisCO activity was measured by adding 0.1 ml of an activation solution to the supernatant. The activation solution consisted of 33 mM Tris–HCl (pH 7.5), 0.67 mM EDTA, 33 mM MgCl_2_, and 10 mM sodium bicarbonate (NaHCO_3_). The mixture was incubated for 15 min, followed by the addition of RuBisCO enzyme. The initial absorbance was measured at 340 nm. To determine the total RuBisCO activity, the mixture was further incubated for 5 min, and the absorbance was measured at 340 nm after 90 s.

Structural Equation Model (SEM) was constructed using a conceptual Meta model (Fig. S6, Fig. 5A,B) with the vital input parameters (Eco-physiological, Morphological, Biochemical characterization) of selected tree species (seven native species and *Prosopis Juliflora)*; the rationale behind the constructed model is described in Table S5. SEMs for the species were fitted using the *LAVAAN* package^147^. Significance and goodness of fit of the SEMs were tested using multiple model fit validation indices, (i) the robust model chi-square (χ2) with a non-significant *p*-value (*p* > 0.05) indicating that the model-implied covariance matrix equals the observed covariance matrix, (ii) Comparative Fit Index (CFI), (best fit at CFI > 0.95), (iii) Robust root Mean Square Error of Approximation (RMSEA) (best fit at RMSEA < 0.05), and iv) Standardized Root Mean squared Residual (SRMR) (best fit at SRMR < 0.08). The model with the complete best fit was arrived for each species, and each path in the final model was assessed for standardized coefficients, significant contributions, and explained variances (R^2^) per response variable were calculated.

Soil sampling, vegetation survey, field eco-physiological assessments, carbon dynamics measurements, and respective laboratory and field experiments were carried out from Jan 2022 to May 2023. Eco-physiological assessments of tree species were carried out during clear sky (PAR ≥ 1200 μmol m^−2^ s^−1^) in the morning/forenoon when photosynthesis is likely to peak.

### Ethical approval

The authors declare that the research was carried out in compliance with the IUCN Policy Statement on Research Involving Species at Risk of Extinction and the Convention on the Trade in Endangered Species of Wild Fauna and Flora.

### Supplementary Information

Supplementary Information.

## Data Availability

The datasets generated during and/or analyzed during the current study are available from the corresponding author on reasonable request.
