# Supplementary material for: Characterization of carbon fluxes, stock and nutrients in the sacred forest groves and invasive vegetation stands within the human dominated landscapes of a tropical semi-arid region
Source: Sci Rep. 2024 Feb 24;14:4513. doi: 10.1038/s41598-024-55294-0 (PMC10894248; doi:10.1038/s41598-024-55294-0)
Supplement: Supplementary file 1 — Supplementary Information. [file 41598_2024_55294_MOESM1_ESM.docx]

**Supplementary files**

**Characterization of carbon fluxes, stock and nutrients in the sacred forest groves and invasive vegetation stands within the human dominated landscapes of a tropical semi-arid region**

Akil Prasath R.V ^a^, Mohanraj R.^a^, Balaramdas K.R.^a^, Jhony Kumar Tagore A.^b^, Raja P.^b^, Rajasekaran A.^c^

a Department of Environmental Science and Management, Bharathidasan University, Tiruchirappalli 620024, India

b St. Joseph’s College, India

c Institute of Forest Genetics and Tree Breeding, Coimbatore 641002, India

**Corresponding author contact:** mohan@bdu.ac.in (R. Mohanraj).

**Table S1.** Location details, anthropogenic pressure, Elevation and Ambient CO_2_ (ppm - Annual mean) levels in Sacred Groves Stands and *Prosopis* *juliflora* Stands

| **Site** | **Longitude** | **Latitude** | **Location type** | **Elevation (meter)** | **Ambient CO_2_** | **Anthropogenic pressure** |
| --- | --- | --- | --- | --- | --- | --- |
| SGS 1 | 79°19'17.20"E | 11°13'4.12"N | Rural | 89 | 384 | Cattle Grazing, Wood Logging |
| SGS 2 | 79° 6'38.91"E | 11° 3'49.75"N | Rural | 92 | 382 | Cattle Grazing |
| SGS 3 | 79° 5'18.30"E | 11° 8'9.28"N | Urban | 88 | 407 | Vehicular , industrial emission, Cattle Grazing, Wood Logging |
| SGS 4 | 79°13'38.80"E | 11° 2'42.88"N | Urban | 68 | 410 | Vehicular emission, Cattle Grazing, Wood Logging |
| SGS 5 | 79°10'52.37"E | 11° 4'5.64"N | Rural | 62 | 395 | Cattle Grazing, Wood Logging |
| SGS 6 | 79°11'22.22"E | 10°57'25.71N | Urban | 92 | 409 | Vehicular emission, Cattle Grazing, Wood Logging |
| SGS 7 | 79° 7'17.66"E | 11° 9'58.82"N | Rural | 95 | 395 | Cattle Grazing |
| SGS 8 | 79° 9'37.63"E | 11°13'1.90"N | Rural | 90 | 396 | Cattle Grazing |
| SGS 9 | 79°30'3.28"E | 11°16'52.75"N | Rural | 91 | 400 | Cattle Grazing |
| SGS 10 | 79°13'27.94"E | 11°18'16.82"N | Rural | 110 | 400 | Cattle Grazing |
| SGS 11 | 78°43'42.18"E | 10°38'24.52"N | Rural | 109 | 397 | Cattle Grazing |
| SGS 12 | 78°43'21.94"E | 10°38'26.47"N | Rural | 105 | 395 | Cattle Grazing |
| SGS 13 | 78°37'59.14"E | 10°52'37.03"N | Rural | 473 | 398 | Cattle Grazing |
| SGS 14 | 78°37'50.86"E | 10°52'39.76"N | Rural | 112 | 397 | Cattle Grazing |
| SGS 15 | 78°26'39.63"E | 11°16'17.33"N | Rural | 110 | 399 | Cattle Grazing |
| SGS 16 | 78°34'51.78"E | 11°18'1.04"N | Rural | 477 | 398 | Cattle Grazing |
| SGS 17 | 78°33'22.94"E | 11°19'34.81"N | Rural | 429 | 397 | Cattle Grazing |
| SGS 18 | 78°34'6.92"E | 11°19'38.86"N | Rural | 439 | 396 | Cattle Grazing |
| SGS 19 | 78°41'2.38"E | 11°12'16.14"N | Rural | 748 | 397 | Cattle Grazing |
| SGS 20 | 79° 9'26.12"E | 10°49'10.97"N | Rural | 65 | 384 | Cattle Grazing |
| SGS 21 | 78°11'46.58"E | 10°15'55.07"N | Rural | 473 | 400 | Cattle Grazing |
| SGS 22 | 78°11'35.82"E | 10°16'33.25"N | Rural | 471 | 400 | Cattle Grazing |
| SGS 23 | 77°46'46.32"E | 10°15'24.60"N | Rural | 472 | 395 | Cattle Grazing |
| SGS 24 | 78° 8'15.89"E | 10°29'10.82"N | Rural | 98 | 398 | Cattle Grazing |
| SGS 25 | 78° 1'29.36"E | 10°21'28.71"N | Rural | 152 | 398 | Cattle Grazing |
| SGS 26 | 78°14'38.41"E | 10°17'50.58"N | Rural | 473 | 385 | Cattle Grazing |
| SGS 27 | 78°15'2.35"E | 10°18'22.55"N | Rural | 475 | 397 | Cattle Grazing |
| SGS 28 | 78°15'37.29"E | 10°19'31.83"N | Rural | 473 | 396 | Cattle Grazing |
| SGS 29 | 78°16'11.41"E | 10°19'53.00"N | Rural | 473 | 396 | Cattle Grazing |
| SGS 30 | 78°13'28.45"E | 10°18'33.17"N | Rural | 452 | 398 | Cattle Grazing |
| SGS 31 | 78°47'5.18"E | 10°22'44.34"N | Rural | 115 | 386 | Cattle Grazing |
| SGS 32 | 79° 0'12.44"E | 10°16'20.55"N | Rural | 111 | 400 | Cattle Grazing |
| SGS 33 | 79° 0'0.23"E | 10°16'58.46"N | Rural | 115 | 391 | Cattle Grazing |
| SGS 34 | 78°56'26.21"E | 10°32'17.12"N | Rural | 110 | 399 | Cattle Grazing |
| SGS 35 | 78°45'0.31"E | 10°37'33.16"N | Urban | 98 | 408 | Vehicular emission |
| SGS 36 | 78°46'27.01"E | 10°35'5.74"N | Rural | 98 | 398 | Cattle Grazing |
| SGS 37 | 78°46'9.46"E | 10°30'49.83"N | Rural | 110 | 398 | Cattle Grazing |
| SGS 38 | 78°43'14.17"E | 10°26'52.11"N | Rural | 119 | 400 | Cattle Grazing |
| SGS 39 | 78°50'7.40"E | 10°21'49.18"N | Urban | 110 | 415 | Vehicular emission |
| SGS 40 | 78°53'2.93"E | 10°13'54.11"N | Urban | 116 | 413 | Vehicular emission |
| SGS 41 | 79° 1'5.21"E | 11°13'51.24"N | Rural | 87 |  | Cattle Grazing |
| SGS 42 | 78°56'49.90"E | 11°18'14.79"N | Urban | 79 | 412 | Vehicular emission |
| SGS 43 | 79° 0'6.53"E | 11°15'29.90"N | Rural | 87 | 403 | Cattle Grazing |
| SGS 44 | 79° 4'10.18"E | 11°14'33.67"N | Rural | 87 | 405 | Cattle Grazing |
| SGS 45 | 79° 3'44.05"E | 11°14'36.71"N | Rural | 86 | 396 | Cattle Grazing |
| SGS 46 | 78°51'41.87"E | 11°11'23.56"N | Rural | 89 | 388 | Cattle Grazing |
| SGS 47 | 79° 6'56.48"E | 11°20'29.31"N | Rural | 86 | 398 | Cattle Grazing |
| SGS 48 | 79° 6'13.77"E | 11°20'52.59"N | Rural | 90 | 401 | Cattle Grazing |
| SGS 49 | 79° 6'10.99"E | 11°20'59.76"N | Rural | 90 | 385 | Cattle Grazing |
| SGS 50 | 78°59'12.72"E | 11°10'51.07"N | Urban | 88 | 409 | Vehicular emission |
| PJS 1 | 11°13'4.12"N | 79°19'17.20"E | Rural | 97 | 396 | Cattle Grazing, Wood Logging |
| PJS 2 | 11° 3'49.75"N | 79° 6'38.91"E | Rural | 97 | 386 | Vehicular emission |
| PJS 3 | 11° 8'9.28"N | 79° 5'18.30"E | Rural | 97 | 396 | Vehicular emission |
| PJS 4 | 11° 2'42.88"N | 79°13'38.80"E | Rural | 97 | 401 | Cattle Grazing, Wood Logging |
| PJS 5 | 11° 4'5.64"N | 79°10'52.37"E | Rural | 97 | 384 | Vehicular emission |
| PJS 6 | 10°57'25.71"N | 79°11'22.22"E | Rural | 98 | 404 | Vehicular emission |
| PJS 7 | 11° 9'58.82"N | 79° 7'17.66"E | Rural | 98 | 401 | Vehicular emission |
| PJS 8 | 11°13'1.90"N | 79° 9'37.63"E | Rural | 98 | 392 | Vehicular emission |
| PJS 9 | 11°16'52.75"N | 79°30'3.28"E | Rural | 97 | 403 | Vehicular emission |
| PJS 10 | 11°18'16.82"N | 79°13'27.94"E | Rural | 96 | 403 | Vehicular emission |
| PJS 11 | 10°38'24.52"N | 78°43'42.18"E | Rural | 112 | 399 | Cattle Grazing, Wood Logging |
| PJS 12 | 10°38'26.47"N | 78°43'21.94"E | Rural | 112 | 396 | Cattle Grazing, Wood Logging |
| PJS 13 | 10°52'37.03"N | 78°37'59.14"E | Rural | 108 | 401 | Cattle Grazing, Wood Logging |
| PJS 14 | 10°52'39.76"N | 78°37'50.86"E | Rural | 110 | 403 | Cattle Grazing, Wood Logging |
| PJS 15 | 11°16'17.33"N | 78°26'39.63"E | Rural | 111 | 397 | Cattle Grazing, Wood Logging |
| PJS 16 | 11°18'1.04"N | 78°34'51.78"E | Rural | 112 | 402 | Cattle Grazing, Wood Logging |
| PJS 17 | 11°19'34.81"N | 78°33'22.94"E | Rural | 116 | 385 | Cattle Grazing, Wood Logging |
| PJS 18 | 11°19'38.86"N | 78°34'6.92"E | Rural | 114 | 395 | Vehicular emission |
| PJS 19 | 11°12'16.14"N | 78°41'2.38"E | Rural | 114 | 382 | Cattle Grazing, Wood Logging |
| PJS 20 | 10°49'10.97"N | 79° 9'26.12"E | Rural | 114 | 402 | Cattle Grazing, Wood Logging |
| PJS 21 | 10°15'55.07"N | 78°11'46.58"E | Rural | 154 | 404 | Vehicular emission |
| PJS 22 | 10°16'33.25"N | 78°11'35.82"E | Rural | 145 | 398 | Vehicular emission |
| PJS 23 | 10°15'24.60"N | 77°46'46.32"E | Rural | 134 | 401 | Vehicular emission |
| PJS 24 | 10°29'10.82"N | 78° 8'15.89"E | Rural | 133 | 403 | Cattle Grazing, Wood Logging |
| PJS 25 | 10°21'28.71"N | 78° 1'29.36"E | Rural | 133 | 385 | Vehicular emission |
| PJS 26 | 10°17'50.58"N | 78°14'38.41"E | Rural | 143 | 405 | Vehicular emission |
| PJS 27 | 10°18'22.55"N | 78°15'2.35"E | Rural | 139 | 384 | Vehicular emission |
| PJS 28 | 10°19'31.83"N | 78°15'37.29"E | Rural | 135 | 398 | Vehicular emission |
| PJS 29 | 10°19'53.00"N | 78°16'11.41"E | Rural | 148 | 404 | Cattle Grazing, Wood Logging |
| PJS 30 | 10°18'33.17"N | 78°13'28.45"E | Urban | 156 | 405 | Vehicular emission, constructions |
| PJS 31 | 10°22'44.34"N | 78°47'5.18"E | Urban | 98 | 415 | Vehicular emission |
| PJS 32 | 10°16'20.55"N | 79° 0'12.44"E | Rural | 111 | 391 | Vehicular emission |
| PJS 33 | 10°16'58.46"N | 79° 0'0.23"E | Rural | 112 | 384 | Cattle Grazing, Wood Logging |
| PJS 34 | 10°32'17.12"N | 78°56'26.21"E | Rural | 112 | 388 | Cattle Grazing, Wood Logging |
| PJS 35 | 10°37'33.16"N | 78°45'0.31"E | Rural | 110 | 395 | Cattle Grazing, Wood Logging |
| PJS 36 | 10°35'5.74"N | 78°46'27.01"E | Urban | 91 | 413 | Vehicular emission |
| PJS 37 | 10°30'49.83"N | 78°46'9.46"E | Rural | 95 | 405 | Cattle Grazing, Wood Logging |
| PJS 38 | 10°26'52.11"N | 78°43'14.17"E | Rural | 98 | 395 | Cattle Grazing, Wood Logging |
| PJS 39 | 10°21'49.18"N | 78°50'7.40"E | Rural | 87 | 402 | Cattle Grazing, Wood Logging |
| PJS 40 | 10°13'54.11"N | 78°53'2.93"E | Rural | 90 | 402 | Vehicular emission |
| PJS 41 | 11°13'51.24"N | 79° 1'5.21"E | Rural | 92 | 384 | Cattle Grazing, Wood Logging |
| PJS 42 | 11°18'14.79"N | 78°56'49.90"E | Rural | 92 | 404 | Cattle Grazing, Wood Logging |
| PJS 43 | 11°15'29.90"N | 79° 0'6.53"E | Rural | 90 | 387 | Cattle Grazing, Wood Logging |
| PJS 44 | 11°14'33.67"N | 79° 4'10.18"E | Rural | 90 | 404 | Cattle Grazing, Wood Logging |
| PJS 45 | 11°14'36.71"N | 79° 3'44.05"E | Urban | 89 | 414 | Highway road, constructions |
| PJS 46 | 11°11'23.56"N | 78°51'41.87"E | Rural | 87 | 395 | Cattle Grazing, Wood Logging |
| PJS 47 | 11°20'29.31"N | 79° 6'56.48"E | Rural | 79 | 390 | Cattle Grazing, Wood Logging |
| PJS 48 | 11°20'52.59"N | 79° 6'13.77"E | Urban | 80 | 410 | Vehicular, industrial emission, constructions |
| PJS 49 | 11°20'59.76"N | 79° 6'10.99"E | Urban | 84 | 409 | Vehicular, industrial emission, constructions |
| PJS 50 | 11°10'51.07"N | 78°59'12.72"E | Rural | 83 | 392 | Cattle Grazing, Wood Logging |

**Table S2.** Agro-climatic zones, soil types of Sacred Groves Stands and *Prosopis* *juliflora* Stands across the study area

| **Agro climatic zone** | **Soil type** | **Locations** |
| --- | --- | --- |
| *Cauvery Delta zone -*  Tiruchirappalli, Thanjavur,  Ariyalur, Perambalur,  Dindigul | Entisols, Inceptisols, Alfisols, Vertisols | **PJS** 4, 5, 6, 7, 8, 9,10, 13, 14, 21, 22, 23, 24, 25, 26, 27, 28, 29, 30, 31, 32, 33, 34, 35 ,36, 37, 38, 39, 40, 41, 42, 43, 44, 45, 46, 47, 48, 49, 50  **SGS** 1, 2, 3, 4, 5, 6, 7, 8,9, 10,13, 14, 15,16, 17, 18, 19, 20, 21, 22, 23, 24, 25, 26, 27, 28, 29, 30, 41, 42, 43, 44, 45, 46, 47, 48, 49, 50 |
| *Southern zone -*  Pudukkottai | Entisols, Inceptisols, Alfisols, Vertisols | **PJS** 1, 2, 3, 11, 12, 15, 16, 17, 18, 19, 20  **SGS** 11, 12, 31, 32, 33, 34, 35, 36, 37, 38, 39, 40 |

**Table S3.** Community weighted Mean comparing the of the proxies of functional traits related to regulatory eco-system services in Sacred Groves Stands and *Prosopis* *juliflora* Stands

| **Communal functional traits** | **SGS** | **PJS** | **Man Whitney U Test** |
| --- | --- | --- | --- |
| Species richness (/ha) | 34 | 9 | <0.01 |
| Shannon Weiner diversity index | 3.65 | 0.46 | <0.01 |
| Family richness (/ha) | 18 | 6 | <0.05 |
| Available nitrogen(mg / kg) | 127 | 108 | <0.05 |
| Available Phosphorus (mg / kg) | 4.8 | 8.9 | <0.05 |
| Available Potassium (mg / kg) | 69.8 | 115 | <0.01 |
| pH | 7.23 | 7.65 | 0.07 |
| EC (dSm-1) | 0.84 | 2.68 | <0.05 |
| Bulk Density (g/cm3) | 1.25 | 1.23 | <0.05 |
| Organic carbon (%) | 0.37 | 0.59 | <0.05 |
| Litter fall (g / m2) | 3.67 | 2.58 | <0.05 |
| Soil Moisture (g/m2) | 7.49 | 5.02 | <0.05 |
| Photosynthesis  (Micro mole CO2/m2/sec) | 3.23 | 3.90 | <0.05 |
| Transpiration  (Micro mole water/m2/sec) | 1.96 | 3.93 | <0.05 |
| Stomatal conductance  (milli mole /m2/sec) | 0.21 | 0.38 | <0.05 |
| Intra cellular CO2 (ppm) | 354 | 365 | <0.07 |
| Total Organic carbon (%) | 0.32 | 0.48 | <0.05 |
| Significance Codes: 0 ‘***’ 0.001 ‘**’ 0.01 ‘*’ 0.05 ‘.’ 0.1 ‘ ’ 1 | | | |

**Table S4.** Functional diversity, evenness of Sacred Groves Stands and *Prosopis* *juliflora* Stands across the study area

|  | **SGS** | **PJS** | **Man Whitney U Test** |
| --- | --- | --- | --- |
| Functional diversity | 0.733 ±  0.09 | 0.538 ±  0.264 | <0.05 |
| Functional evenness | 0.807 ±  0.132 | 0.870 ±  0.145 | 0.07 |


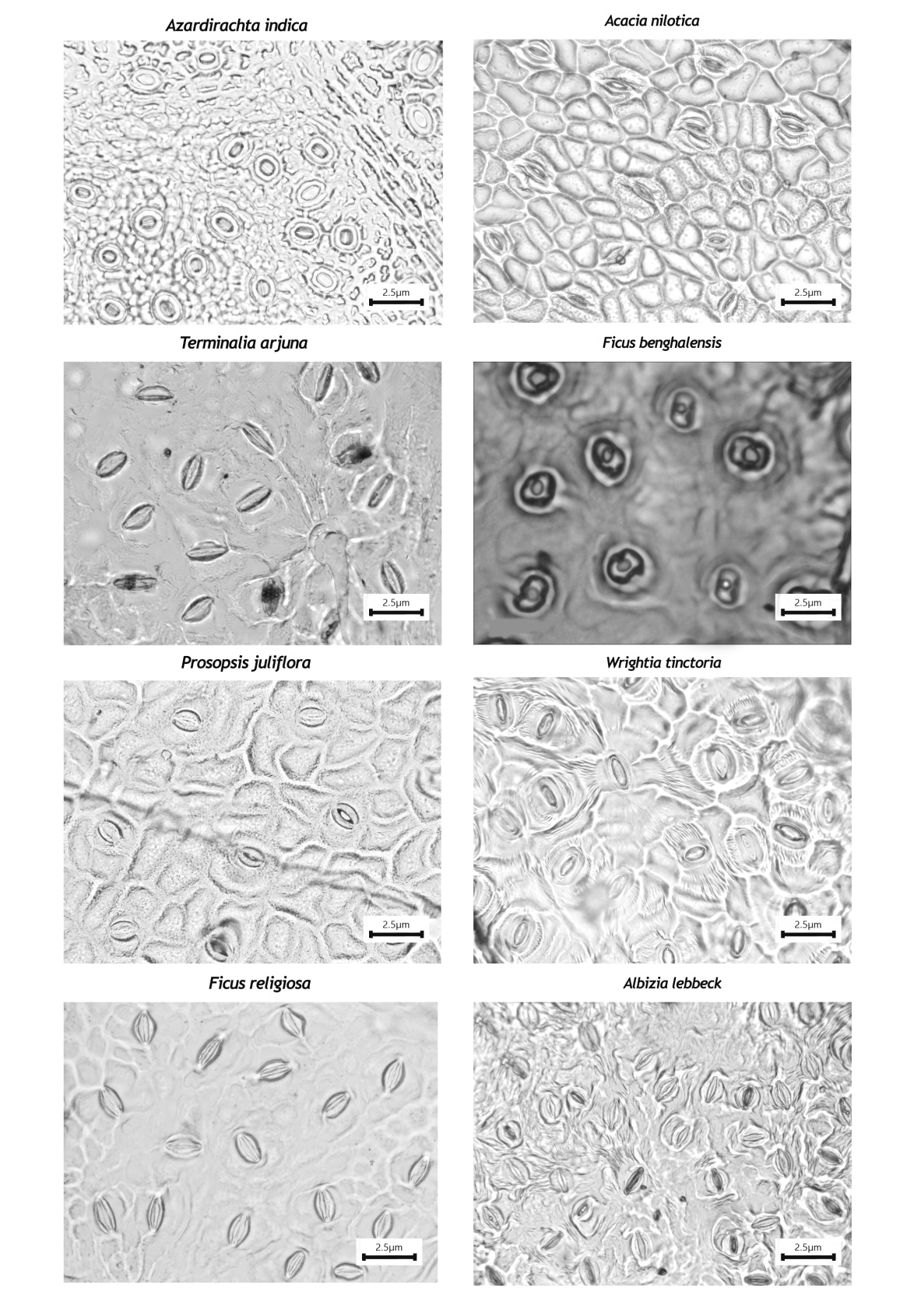


**Fig. S1. |** Stomatal micrographs (40 x magnifications) of selected eight tree species depicting the stomatal morphology and density


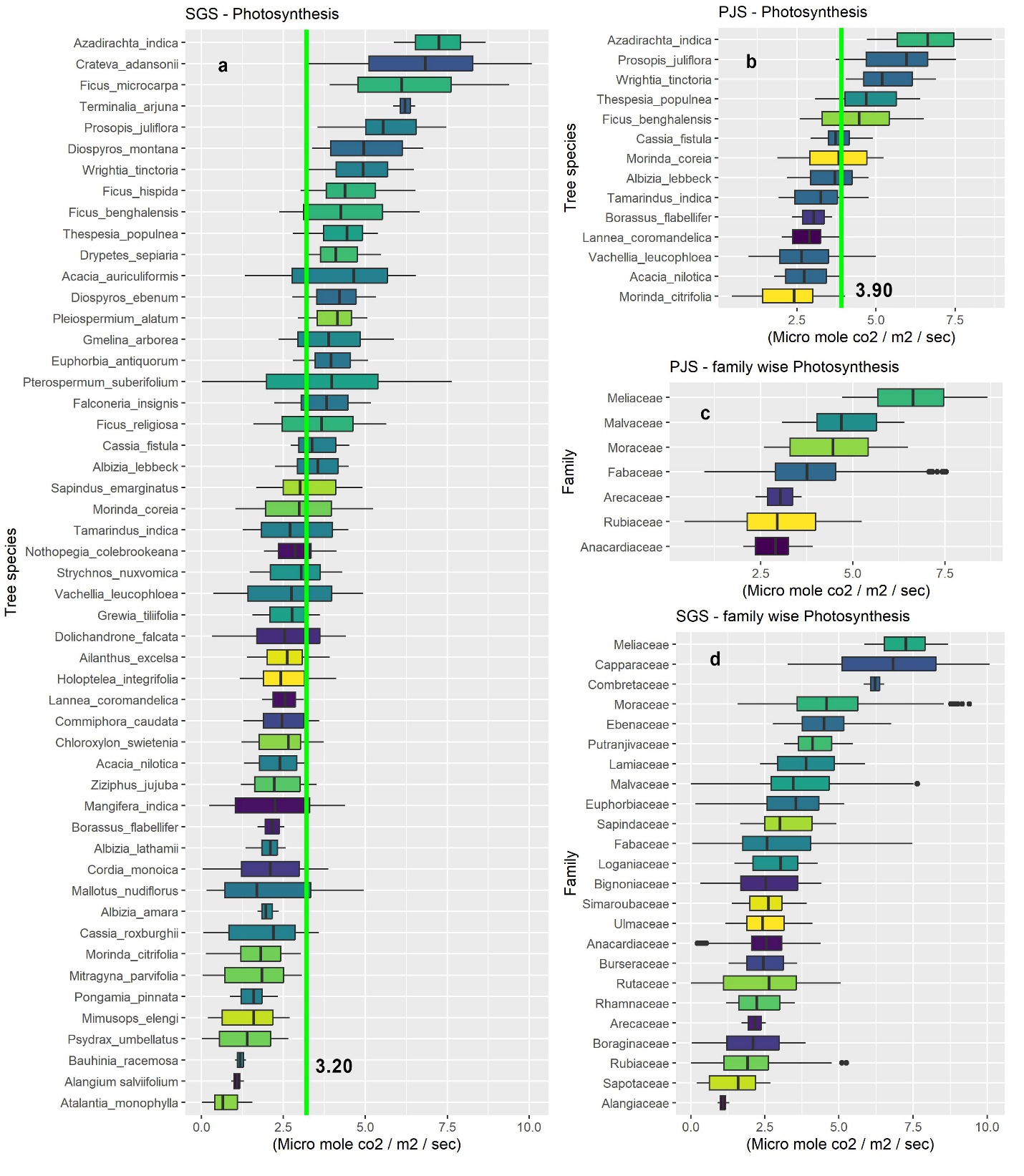


**Fig. S2.** Photosynthesis rate of individual tree species and the respective family in Sacred groves stands and *Prosopis* *juliflora* Stands across the study area


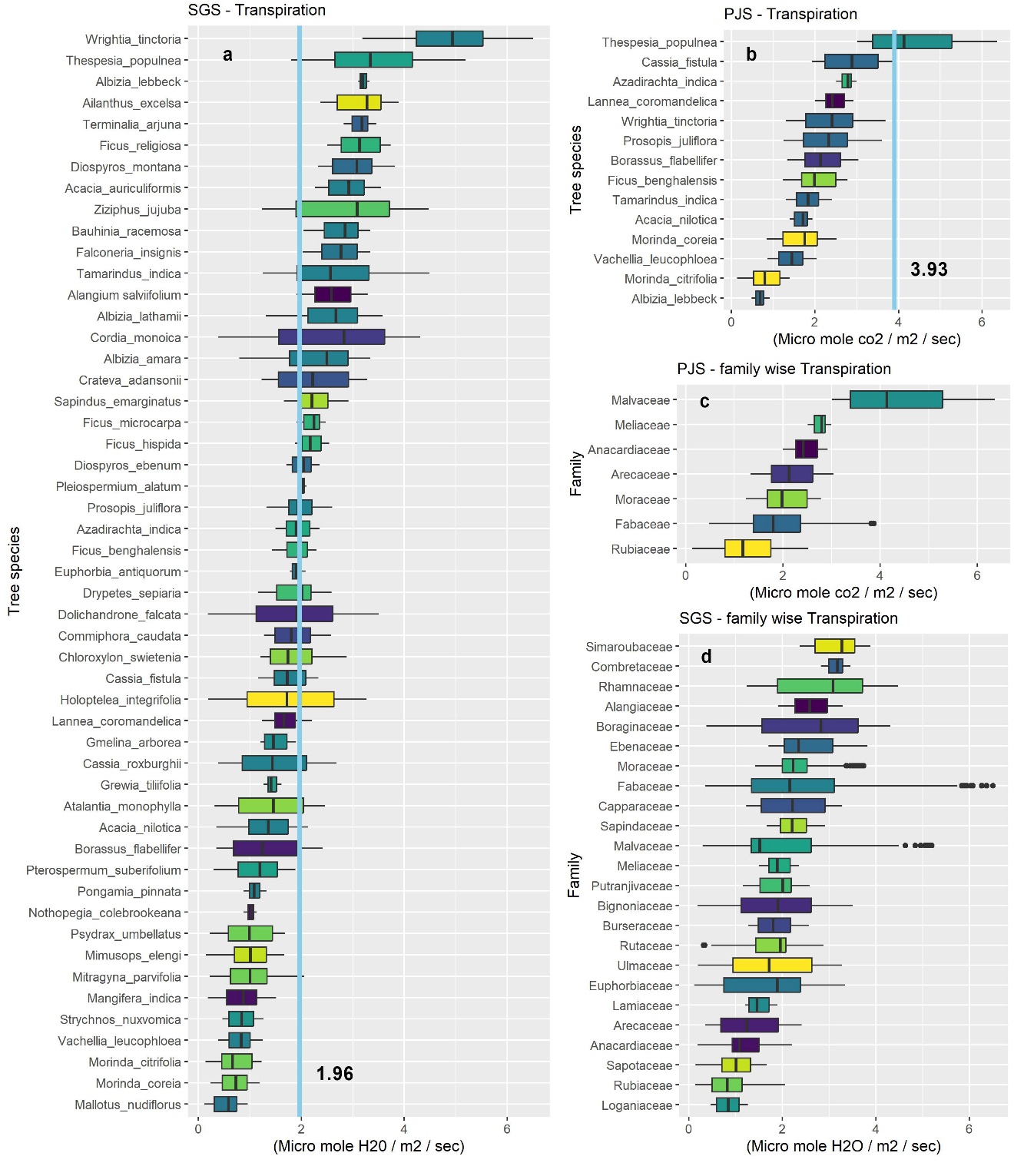


**Fig. S3.** Transpiration rate of individual tree species and the respective family in Sacred Groves Stands and *Prosopis* *juliflora* Stands across the study area


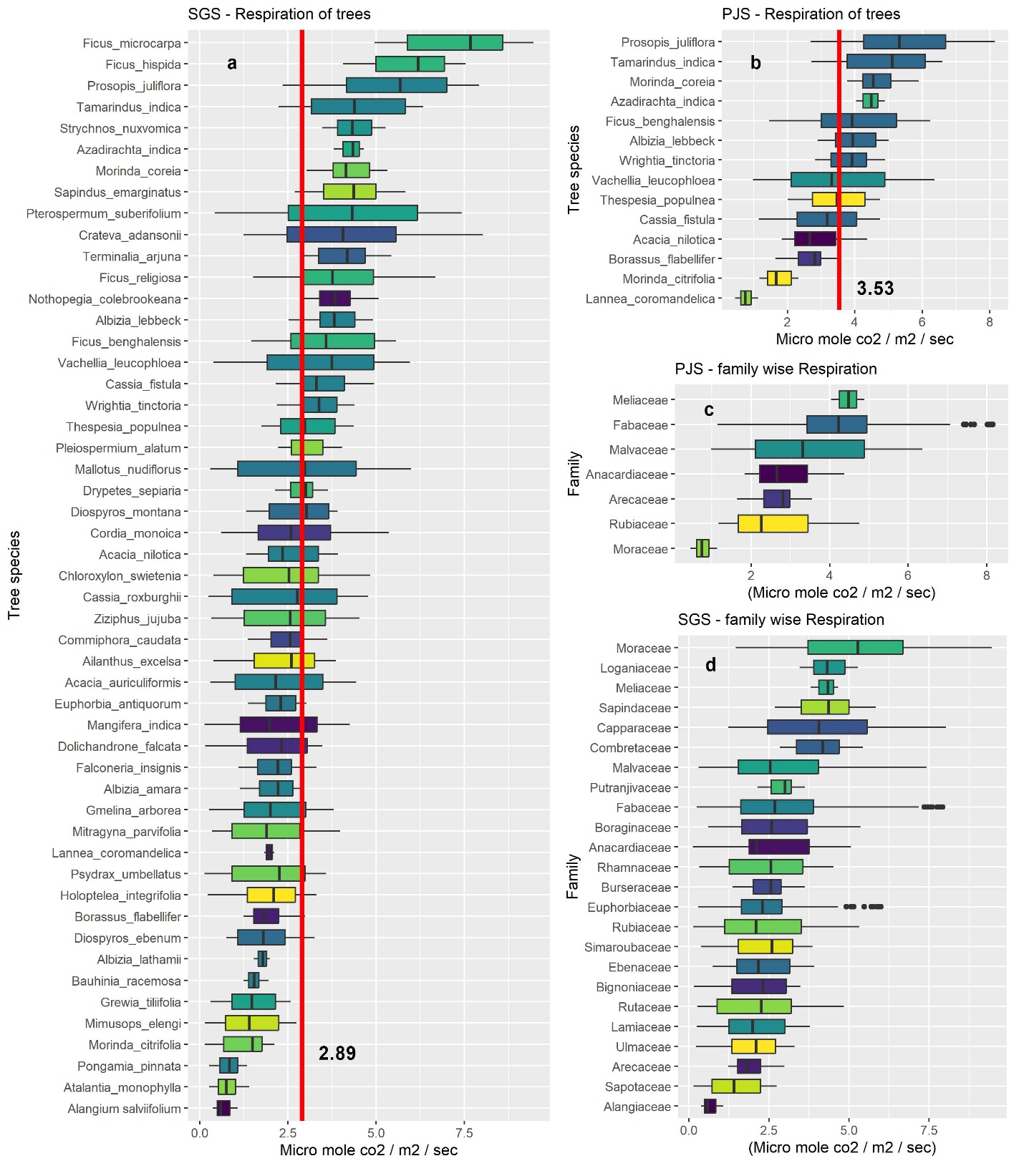


**Fig. S4.** Respiration rate of individual tree species and the respective family in Sacred Groves Stands and *Prosopis* *juliflora* Stands across the study area


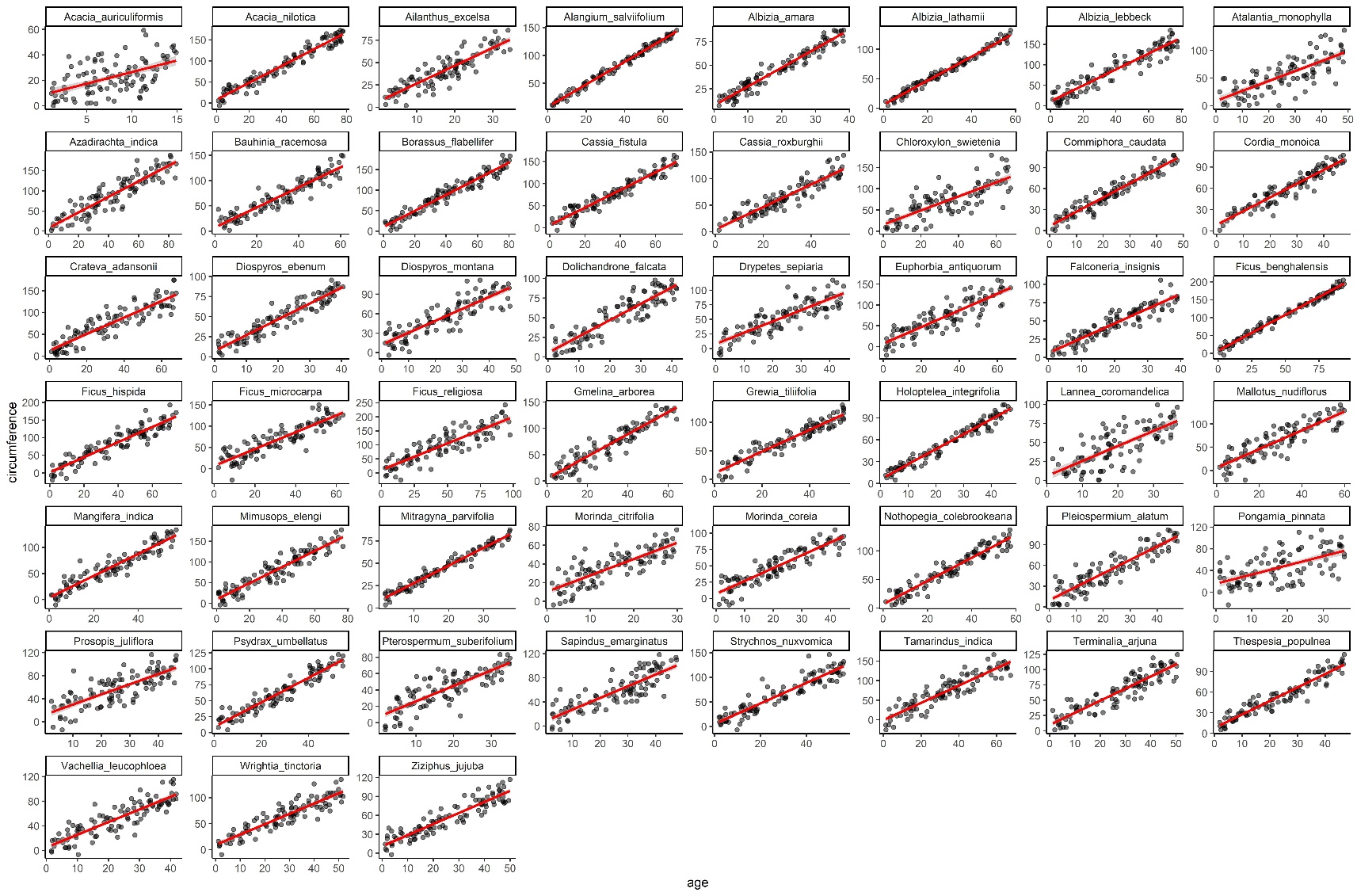


**Fig S5.** Tree age linear model testing the effects on age of selected trees on variation with increase in stem circumference (cm) as a predictor variable. (Parameters based the decision tree model was achieved on the lowest Akaike information criterion (AIC) value.) (Grey bars in parallel with the regression line depict the 95% confidence interval).

Construction of Structural Equation Model (SEM)

A conceptual meta model (Fig S6) was constructed using 11 variables (*pn:* Photosynthesis, *sd:* Stomatal Density, *GS:* Stomatal Conductance, *Temp:* Temperature, *intraco2:* Leaf Intracellular CO_2_, *Amco2:* Ambient CO_2_, *sm:* Soil moisture, *sn:* Soil nitrogen, *rubisco:* Ribulose-1,5-bisphosphate carboxylase/oxygenase, *chla:* Total Chlorophyll, *toc:* Total Organic Carbon). It was hypothesized that exogenous variables do influence the endogenous variable. The rationale behind each hypothesized path between the endogenous and exogenous variable is briefed in Table S5. SEMs for selected species were fitted using the Lavaan package ^142^. Significance and goodness of fit of the SEMs were tested using multiple model fit validation indices: i) the robust model chi-square (χ2) with a non-significant p-value (p > 0.05) indicating that the model-implied covariance matrix equals the observed covariance matrix, ii) Comparative Fit Index (CFI), (best fit at CFI > 0.95), iii) Robust Root Mean Square Error of Approximation (RMSEA) (best fit at RMSEA < 0.05), and iv) Standardized Root Mean squared Residual (SRMR) (best fit at SRMR < 0.08). The model with the complete best fit was arrived for tree species. Each path in the final model was assessed for standardized coefficients, significant contribution, and explained variance (R^2^) per response variable were calculated.

*
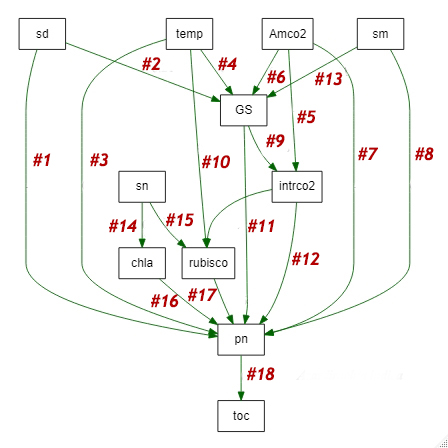
*

*pn:* Photosynthesis, *sd:* Stomatal Density, *GS:* Stomatal Conductance, *Temp:* Temperature, *intraco2:* Leaf Intracellular CO_2_, *Amco2:* Ambient CO_2_, *sm:* Soil moisture, *sn:* Soil nitrogen, *rubisco:* Ribulose-1,5-bisphosphate carboxylase/oxygenase, *chla:* Total Chlorophyll, *toc:* Total Organic Carbon.

**Fig S6.** Meta Model - the rationale behind each path of carbon fixing functional trait is given in Table. S 5

**Table S5.** Rationale behind each hypothesized path in the Meta model Path

| **#Path** | **Pathway** | **Described underlying mechanisms behind the hypothesized relationship** | **Reference** |
| --- | --- | --- | --- |
| 1 | pn ~ sd | Photosynthesis increase with stomatal density | ^151^ |
| 2 | GS ~ sd | Stomatal conductance increased with Stomatal density | ^151^ |
| 3 | pn ~ temp | Photosynthesis ceases with increase in temperature | ^152^ |
| 4 | GS ~ temp | Stomatal conductance increases against temperature | ^99^ |
| 5 | intraco2 ~  Amco2 | Significant increase in Ambient CO_2_ caused increase in intracellular CO_2_ | ^153^ |
| 6 | GS ~ Amco2 | Increasing Ambient CO_2_ drastically increases stomatal conductance | ^94^ |
| 7 | pn ~ Amco2 | Current trends (2023) Ambient CO_2_ does enhance photosynthesis | ^88^ |
| 8 | pn ~sm | High Soil moisture leads to increase in Photosynthesis rate | ^84^ |
| 9 | intraco2 ~ GS | Higher stomatal conductance leads to increased intracellular CO_2_ levels | ^94^ |
| 10 | rubisco ~  temp | Increased temperature delimits RuBisCo | ^98^ |
| 11 | pn ~ GS | Photosynthesis increase with stomatal conductance | ^154^ |
| 12 | pn ~ intraco2 | Increase in intracellular CO_2_ raises photosynthesis | ^155^ |
| 13 | GS ~ sm | A linear relation was observed between Stomatal conductance and soil moisture | ^154^ |
| 14 | chla ~ sn | Leaf chlorophyll increase with soil nitrogen content | ^156^ |
| 15 | rubisco ~ sn | Soil nitrogen significantly correlated with rubisco | ^157^ |
| 16 | pn ~ chla | Chlorophyll and photosynthesis are positively correlated | ^96^ |
| 17 | pn ~ rubisco | RuBisCO and photosynthesis are positively correlated | ^158^ |
| 18 | toc ~ pn | Total organic carbon increases with an increase in Photosynthesis | ^159^ |

***Model validation for selected tree species***

1. *Azardirachta indica*

Model Test User Model:

Test statistic 3.112

Degrees of freedom 26

P-value (Chi-square) 0.015

Model Test Baseline Model:

Test statistic 58.086

Degrees of freedom 45

P-value 0.091

User Model versus Baseline Model:

Comparative Fit Index (CFI) 0.957

Tucker-Lewis Index (TLI) 0.959

Root Mean Square Error of Approximation:

RMSEA 0.074

90 Percent confidence interval - lower 0.000

90 Percent confidence interval - upper 0.142

P-value H_0: RMSEA <= 0.050 0.291

P-value H_0: RMSEA >= 0.080 0.082

Standardized Root Mean Square Residual:

SRMR 0.077

Parameter Estimates:

Standard errors Standard

Information Expected

Information saturated (h1) model Structured

Regressions:

Estimate Std.Err z-value P(>|z|) Std.lv Std.all

chla ~

sn 0.067 0.003 2.510 0.029 -0.027 -0.210

rubisco ~

sn 0.015 0.002 0.044 0.965 0.000 0.006

pn ~

sd -0.012 0.002 1.741 0.082 0.003 0.227

rubisco 0.465 0.253 -1.920 0.055 -0.485 -0.263

chla 0.683 0.286 -0.464 0.642 -0.133 -0.060

temp 0.077 0.476 1.695 0.090 0.807 0.760

intraco2 -0.054 0.007 -0.653 0.043 -0.004 -0.093

sm 0.275 0.420 0.440 0.005 0.185 0.259

Amco2 -0.032 0.086 0.691 0.490 0.060 0.485

GS ~

Amco2 0.070 0.005 0.863 0.388 0.004 0.647

intrco2 ~

Amco2 -0.018 0.364 -1.019 0.308 -0.371 -0.138

rubisco ~

temp 0.015 0.077 0.053 0.958 0.004 0.007

intrco2 -0.022 0.003 -2.653 0.008 -0.009 -0.353

toc ~

pn 0.738 0.653 0.264 0.022 0.173 0.037

GS ~

temp 0.109 0.027 -0.344 0.731 -0.009 -0.166

sd 0.211 0.000 0.144 0.885 0.000 0.020

pn ~

GS 2.861 2.626 0.967 0.334 2.538 0.132

GS ~

sm -0.015 0.023 -1.084 0.279 -0.025 -0.680

intrco2 ~

GS 3.963 56.556 2.192 0.028 23.963 0.298

Variances:

Estimate Std.Err z-value P(>|z|) Std.lv Std.all

.chla 0.255 0.051 5.000 0.000 0.255 0.956

.rubisco 0.339 0.068 5.000 0.000 0.339 0.876

.pn 1.081 0.216 5.000 0.000 1.081 0.820

.GS 0.003 0.001 5.000 0.000 0.003 0.955

.intrco2 382.105 2.421 5.000 0.000 2.105 0.904

.toc 8.081 5.616 5.000 0.000 8.081 0.999

R-Square:

Estimate

chla 0.644

rubisco 0.424

pn 0.280

GS 0.445

intrco2 0.096

toc 0.441

1. *Albizia Lebbeck*

Model Test User Model:

Test statistic 17.393

Degrees of freedom 26

P-value (Chi-square) 0.897

Model Test Baseline Model:

Test statistic 52.240

Degrees of freedom 45

P-value 0.213

User Model versus Baseline Model:

Comparative Fit Index (CFI) 1.000

Tucker-Lewis Index (TLI) 3.058

Root Mean Square Error of Approximation:

RMSEA 0.000

90 Percent confidence interval - lower 0.000

90 Percent confidence interval - upper 0.050

P-value H_0: RMSEA <= 0.050 0.949

P-value H_0: RMSEA >= 0.080 0.015

Standardized Root Mean Square Residual:

SRMR 0.057

Parameter Estimates:

Standard errors Standard

Information Expected

Information saturated (h1) model Structured

Regressions:

Estimate Std.Err z-value P(>|z|) Std.lv Std.all

chla ~

sn 0.139 0.006 -1.650 0.099 -0.009 -0.227

rubisco ~

sn 0.090 0.006 -1.594 0.111 -0.010 -0.218

pn ~

sd -0.980 0.003 -0.060 0.952 -0.000 -0.007

rubisco 0.192 0.137 1.401 0.161 0.192 0.168

chla 0.239 0.147 1.627 0.104 0.239 0.192

temp 0.197 0.086 2.301 0.021 0.197 0.277

intraco2 -2.990 0.004 -0.032 0.975 -0.000 -0.004

sm 0.609 0.256 -2.379 0.017 -0.609 -1.275

Amco2 0.127 0.044 2.883 0.004 0.127 1.542

GS ~

Amco2 0.060 0.001 0.243 0.808 0.000 0.147

intraco2 ~

Amco2 0.552 0.338 1.634 0.102 0.552 0.231

rubisco ~

temp 0.041 0.085 0.479 0.632 0.041 0.066

intraco2 0.055 0.004 1.143 0.253 0.005 0.155

toc ~

pn 0.973 0.562 1.571 0.016 0.883 0.217

GS ~

temp 0.124 0.003 1.454 0.146 0.004 0.195

sd 0.620 0.000 0.149 0.882 0.000 0.020

pn ~

GS 8.270 4.633 1.785 0.074 8.270 0.222

GS ~

sm 0.010 0.008 0.659 0.510 0.005 0.400

intraco2 ~

GS 3.660 1.063 0.738 0.461 0.540 0.104

Variances:

Estimate Std.Err z-value P(>|z|) Std.lv Std.all

.chla 0.360 0.072 5.000 0.000 0.360 0.948

.rubisco 0.415 0.083 5.000 0.000 0.415 0.921

.pn 0.407 0.081 5.000 0.000 0.407 0.689

.GS 0.000 0.000 5.000 0.000 0.000 0.899

.intraco2 408.888 93.778 5.000 0.000 408.888 0.947

.toc 9.333 1.867 5.000 0.000 9.333 0.953

R-Square:

Estimate

chla 0.252

rubisco 0.179

pn 0.311

GS 0.101

intraco2 0.053

toc 0.747

1. *Ficus Benghalensis*

Model Test User Model:

Test statistic 6.437

Degrees of freedom 26

P-value (Chi-square) 0.439

Model Test Baseline Model:

Test statistic 41.903

Degrees of freedom 45

P-value 0.604

User Model versus Baseline Model:

Comparative Fit Index (CFI) 0.970

Tucker-Lewis Index (TLI) 0.944

Root Mean Square Error of Approximation:

RMSEA 0.018

90 Percent confidence interval - lower 0.000

90 Percent confidence interval - upper 0.114

P-value RMSEA <= 0.05 0.011

Standardized Root Mean Square Residual:

SRMR 0.077

Parameter Estimates:

Standard errors Standard

Information Expected

Information saturated (h1) model Structured

Regressions:

Estimate Std.Err z-value P(>|z|) Std.lv Std.all

chla ~

sn 0.093 0.005 -0.679 0.007 -0.003 -0.096

rubisco ~

sn 0.062 0.010 -0.161 0.002 -0.002 -0.023

pn ~

sd -0.015 0.001 -0.996 0.009 -0.001 -0.132

rubisco 0.261 0.142 -1.837 0.006 -0.261 -0.228

chla 0.021 0.276 0.066 0.007 0.018 0.008

temp 0.332 0.140 2.363 0.008 0.332 0.309

intraco2 -0.014 0.005 0.806 0.000 0.004 0.100

sm 0.147 0.411 -0.357 0.001 -0.147 -0.204

Amco2 0.034 0.071 0.473 0.006 0.034 0.270

GS ~

Amco2 0.011 0.001 -1.167 0.003 -0.001 -0.721

intraco2 ~

Amco2 -0.143 0.442 -0.324 0.006 -0.143 -0.046

rubisco ~

temp -0.016 0.134 -0.044 0.005 -0.006 -0.006

intraco2 0.080 0.005 -0.029 0.007 -0.000 -0.004

toc ~

pn 0.372 0.568 0.250 0.002 0.142 0.035

GS ~

temp 0.022 0.002 0.793 0.008 0.002 0.113

sd 0.010 0.000 0.994 0.000 0.000 0.143

pn ~

GS 1.524 8.156 1.981 0.048 6.154 0.256

GS ~

sm 0.010 0.007 1.193 0.003 0.008 0.735

intraco2 ~

GS 7.124 4.316 -0.138 0.040 0.944 -0.019

Variances:

Estimate Std.Err z-value P(>|z|) Std.lv Std.all

.chla 0.269 0.054 5.000 0.000 0.269 0.991

.rubisco 1.029 0.206 5.000 0.000 1.029 0.999

.pn 1.035 0.207 5.000 0.000 1.035 0.770

.GS 0.000 0.000 5.000 0.000 0.000 0.925

.intraco2 446.251 169.250 5.000 0.000 846.251 0.998

.toc 21.669 4.334 5.000 0.000 21.669 0.999

R-Square:

Estimate

chla 0.609

rubisco 0.101

pn 0.230

GS 0.275

intraco2 0.602

toc 0.658

1. ***Ficus religiosa***

Model Test User Model:

Test statistic 2.182

Degrees of freedom 26

P-value (Chi-square) 0.679

Model Test Baseline Model:

Test statistic 8.648

Degrees of freedom 45

P-value 0.328

User Model versus Baseline Model:

Comparative Fit Index (CFI) 0.982

Tucker-Lewis Index (TLI) 0.911

Loglikelihood and Information Criteria:

Loglikelihood user model (H0) -522.353

Loglikelihood unrestricted model (H1) -511.262

Akaike (AIC) 1094.706

Bayesian (BIC) 1142.506

Sample-size adjusted Bayesian (SABIC) 1064.036

Root Mean Square Error of Approximation:

RMSEA 0.000

90 Percent confidence interval - lower 0.000

90 Percent confidence interval - upper 0.091

P-value H_0: RMSEA <= 0.050 0.806

P-value H_0: RMSEA >= 0.080 0.079

Standardized Root Mean Square Residual:

SRMR 0.075

Parameter Estimates:

Standard errors Standard

Information Expected

Information saturated (h1) model Structured

Regressions:

Estimate Std.Err z-value P(>|z|) Std.lv Std.all

chla ~

sn 0.082 0.006 -0.364 0.016 -0.002 -0.051

rubisco ~

sn 0.061 0.006 0.406 0.085 0.002 0.058

pn ~

sd -0.061 0.003 0.251 0.002 0.001 0.031

rubisco 0.587 0.285 2.060 0.039 0.587 0.237

chla 0.396 0.254 1.557 0.019 0.396 0.179

temp 0.246 0.164 -1.497 0.034 -0.246 -0.179

intraco2 -0.093 0.003 0.943 0.046 0.003 0.110

sm 1.744 0.484 -3.606 0.000 -1.744 -1.894

Amco2 0.286 0.083 3.435 0.001 0.286 1.800

GS ~

Amco2 0.019 0.002 -0.042 0.066 -0.000 -0.026

intraco2 ~

Amco2 0.476 0.800 -0.596 0.051 -0.476 -0.083

rubisco ~

temp 0.020 0.079 0.249 0.003 0.020 0.035

intraco2 -0.051 0.002 -0.160 0.073 -0.000 -0.023

toc ~

pn 0.277 1.005 -0.275 0.083 -0.277 -0.039

GS ~

temp 1.652 0.003 -0.404 0.086 -0.001 -0.057

sd 0.112 0.000 1.537 0.024 0.000 0.221

pn ~

GS 0.131 6.724 0.816 0.014 5.488 0.098

GS ~

sm -0.121 0.010 0.156 0.076 0.002 0.098

intraco2 ~

GS 0.110 0.941 1.125 0.061 316.021 0.157

Variances:

Estimate Std.Err z-value P(>|z|) Std.lv Std.all

.chla 0.451 0.090 5.000 0.000 0.451 0.997

.rubisco 0.358 0.072 5.000 0.000 0.358 0.995

.pn 1.458 0.292 5.000 0.000 1.458 0.664

.GS 0.001 0.000 5.000 0.000 0.001 0.939

.intraco2 411.525 553.305 5.000 0.000 407.525 0.970

.toc 41.067 22.213 5.000 0.000 11.067 0.998

R-Square:

Estimate

chla 0.713

rubisco 0.205

pn 0.336

GS 0.461

intraco2 0.630

toc 0.402

1. ***Prosopis juliflora***

Model Test User Model:

Test statistic 6.512

Degrees of freedom 26

P-value (Chi-square) 0.093

Model Test Baseline Model:

Test statistic 1192.958

Degrees of freedom 45

P-value 0.000

User Model versus Baseline Model:

Comparative Fit Index (CFI) 0.960

Tucker-Lewis Index (TLI) 0.958

Root Mean Square Error of Approximation:

RMSEA 0.003

90 Percent confidence interval - lower 0.002

90 Percent confidence interval - upper 0.109

P-value RMSEA <= 0.05 0.110

Standardized Root Mean Square Residual:

SRMR 0.012

Parameter Estimates:

Standard errors Standard

Information Expected

Information saturated (h1) model Structured

Regressions:

Estimate Std.Err z-value P(>|z|) Std.lv Std.all

chla ~

sn 0.119 0.004 30.180 0.000 0.109 0.974

rubisco ~

sn 0.027 0.014 1.216 0.024 0.017 0.395

pn ~

sd 0.059 0.051 -0.947 0.043 -0.049 -0.083

rubisco 0.392 0.187 2.103 0.035 0.392 0.079

chla 0.243 0.138 1.762 0.018 0.243 0.126

temp 0.270 0.185 1.443 0.049 0.267 0.117

intraco2 0.074 0.028 -2.612 0.009 -0.074 -0.304

sm 0.198 0.146 1.283 0.000 0.188 0.122

Amco2 0.163 0.046 3.506 0.010 0.163 0.614

GS ~

Amco2 0.011 0.001 -6.583 0.000 -0.009 -0.652

intraco2 ~

Amco2 1.652 0.164 -6.131 0.000 -1.006 -0.916

rubisco ~

temp -0.349 0.115 -3.036 0.006 -0.349 -0.754

intraco2 0.026 0.014 -1.836 0.066 -0.026 -0.522

toc ~

pn 1.221 0.050 45.832 0.000 2.281 0.988

GS ~

temp 0.028 0.008 3.391 0.001 0.028 0.227

sd 0.009 0.002 -2.010 0.044 -0.005 -0.152

pn ~

GS 3.732 2.655 1.406 0.060 3.732 0.204

GS ~

sm -0.023 0.007 -3.243 0.001 -0.023 -0.273

intraco2 ~

GS 0.558 11.327 0.485 0.027 5.498 0.072

Variances:

Estimate Std.Err z-value P(>|z|) Std.lv Std.all

.chla 0.085 0.017 5.000 0.000 0.085 0.052

.rubisco 0.058 0.012 5.000 0.000 0.058 0.230

.pn 0.101 0.020 5.000 0.000 0.101 0.016

.GS 0.000 0.000 5.000 0.000 0.000 0.016

.intraco2 372.622 0.524 5.000 0.000 372.62 0.025

.toc 0.758 0.152 5.000 0.000 0.758 0.023

R-Square:

Estimate

chla 0.548

rubisco 0.370

pn 0.684

GS 0.784

intraco2 0.675

toc 0.177

1. ***Terminalia arjuna***

Model Test User Model:

Test statistic 3.512

Degrees of freedom 26

P-value (Chi-square) 0.043

signifies model fits the data or data fits the model

Model Test Baseline Model:

Test statistic 1592.958

Degrees of freedom 43

P-value 0.000

User Model versus Baseline Model:

Comparative Fit Index (CFI) 0.950

Tucker-Lewis Index (TLI) 0.938

Root Mean Square Error of Approximation:

RMSEA 0.007

90 Percent confidence interval - lower 0.044

90 Percent confidence interval - upper 0.123

P-value RMSEA <= 0.05 0.123

Standardized Root Mean Square Residual:

SRMR 0.035

Parameter Estimates:

Standard errors Standard

Information Expected

Information saturated (h1) model Structured

Regressions:

Estimate Std.Err z-value P(>|z|) Std.lv Std.all

chla ~

sn 0.119 0.004 30.180 0.000 0.409 0.974

rubisco ~

sn 0.010 0.014 -2.216 0.014 -0.117 -0.295

pn ~

sd 0.039 0.551 -1.847 0.003 -0.049 -0.383

rubisco 0.124 0.137 3.103 0.055 0.292 0.279

chla 0.040 0.128 3.462 0.068 0.243 0.526

temp 0.340 0.285 2.423 0.049 0.467 0.517

intraco2 0.116 0.028 -2.632 0.029 -0.174 -0.104

sm 0.231 0.346 1.283 0.010 0.288 0.122

Amco2 0.094 0.546 3.506 0.030 0.163 0.614

GS ~

Amco2 0.021 0.001 -6.583 0.011 -0.009 -0.552

intraco2 ~

Amco2 1.656 0.164 -6.131 0.001 -1.306 -0.516

rubisco ~

temp 0.299 0.115 -3.036 0.001 -0.449 -0.854

intraco2 0.046 0.014 -1.836 0.056 -0.026 -0.222

toc ~

pn 0.261 0.050 45.832 0.000 2.481 0.188

GS ~

temp 0.038 0.008 3.391 0.002 0.128 0.227

sd 0.192 0.002 -2.010 0.042 -0.005 -0.152

pn ~

GS 0.321 2.655 1.406 0.032 1.132 0.204

GS ~

sm -0.081 0.007 -3.243 0.043 -0.123 -0.273

intraco2 ~

GS 5.231 11.327 0.485 0.011 5.298 0.072

Variances:

Estimate Std.Err z-value P(>|z|) Std.lv Std.all

.chla 0.023 0.027 5.000 0.000 0.085 0.052

.rubisco 0.011 0.012 5.000 0.000 0.058 0.230

.pn 0.154 0.021 5.000 0.000 0.101 0.016

.GS 0.046 0.022 5.000 0.000 0.000 0.016

.intraco2 1.124 0.312 5.000 0.000 2.622 0.025

.toc 0.248 0.552 5.000 0.000 0.758 0.023

R-Square:

Estimate

chla 0.248

rubisco 0.570

pn 0.284

GS 0.584

intraco2 0.212

toc 0.347

1. ***Wrightia tinctoria***

Model Test User Model:

Test statistic 0.487

Degrees of freedom 26

P-value (Chi-square) 0.768

Model Test Baseline Model:

Test statistic 38.734

Degrees of freedom 45

P-value 0.733

User Model versus Baseline Model:

Comparative Fit Index (CFI) 0.980

Tucker-Lewis Index (TLI) 0.923

Root Mean Square Error of Approximation:

RMSEA 0.000

90 Percent confidence interval - lower 0.000

90 Percent confidence interval - upper 0.079

P-value H_0: RMSEA <= 0.050 0.870

P-value H_0: RMSEA >= 0.080 0.048

Standardized Root Mean Square Residual:

SRMR 0.068

Parameter Estimates:

Standard errors Standard

Information Expected

Information saturated (h1) model Structured

Regressions:

Estimate Std.Err z-value P(>|z|) Std.lv Std.all

chla ~

sn 0.321 0.009 -0.195 0.046 -0.002 -0.028

rubisco ~

sn 0.145 0.010 -1.456 0.045 -0.015 -0.194

pn ~

sd 0.163 0.001 -0.210 0.034 -0.000 -0.029

rubisco 0.165 0.121 1.363 0.073 0.165 0.190

chla 0.022 0.136 0.164 0.070 0.022 0.022

temp 0.081 0.129 -0.316 0.052 -0.041 -0.043

intraco2 0.593 0.013 -1.748 0.080 -0.023 -0.247

sm 0.211 0.390 -1.537 0.024 -0.599 -0.954

Amco2 0.092 0.067 1.375 0.039 0.092 0.851

GS ~

Amco2 0.092 0.001 1.526 0.027 0.002 0.944

intraco2 ~

Amco2 0.442 0.161 -1.174 0.040 -0.189 -0.164

rubisco ~

temp 0.028 0.144 0.198 0.043 0.028 0.026

intraco2 0.031 0.014 2.187 0.029 0.031 0.290

toc ~

pn 1.288 0.637 -1.519 0.029 -0.968 -0.210

GS ~

temp 0.023 0.003 0.082 0.034 0.000 0.012

sd 0.111 0.000 -0.773 0.039 -0.000 -0.109

pn ~

GS 0.631 6.365 -0.204 0.038 -1.298 -0.028

GS ~

sm -0.014 0.008 -1.626 0.004 -0.014 -1.005

intraco2 ~

GS 1.223 68.278 -0.127 0.099 -8.693 -0.018

Variances:

Estimate Std.Err z-value P(>|z|) Std.lv Std.all

.chla 0.982 0.196 5.000 0.000 0.982 0.999

.rubisco 1.189 0.238 5.000 0.000 1.189 0.876

.pn 0.909 0.182 5.000 0.000 0.909 0.888

.GS 0.000 0.000 5.000 0.000 0.000 0.931

.intraco2 402.207 10.441 5.000 0.000 402.207 0.973

.toc 20.775 4.155 5.000 0.000 20.775 0.956

R-Square:

Estimate

chla 0.511

rubisco 0.624

pn 0.612

GS 0.069

intraco2 0.127

toc 0.544

1. ***Acacia nilotica***

Model Test User Model:

Test statistic 62.723

Degrees of freedom 26

P-value (Chi-square) 0.000

Model Test Baseline Model:

Test statistic 1.550

Degrees of freedom 45

P-value 0.230

User Model versus Baseline Model:

Comparative Fit Index (CFI) 0.981

Tucker-Lewis Index (TLI) 0.903

Root Mean Square Error of Approximation:

RMSEA 0.007

90 Percent confidence interval - lower 0.381

90 Percent confidence interval - upper 0.474

P-value H_0: RMSEA <= 0.050 0.000

P-value H_0: RMSEA >= 0.080 0.010

Standardized Root Mean Square Residual:

SRMR 0.029

Parameter Estimates:

Standard errors Standard

Information Expected

Information saturated (h1) model Structured

Regressions:

Estimate Std.Err z-value P(>|z|) Std.lv Std.all

chla ~

sn 0.781 0.005 0.873 0.013 0.004 0.123

rubisco ~

sn 0.334 0.001 25.357 0.000 0.014 0.538

pn ~

sd -0.042 0.016 -1.561 0.019 -0.025 -0.235

rubisco 0.019 2.221 2.710 0.007 6.019 0.731

chla 0.249 0.317 -1.547 0.022 -0.491 -0.081

temp 0.020 0.163 0.120 0.004 0.020 0.006

intraco2 0.112 0.031 5.618 0.000 0.174 1.287

sm 0.611 0.484 -0.629 0.029 -0.304 -0.149

Amco2 0.132 0.083 0.196 0.044 0.016 0.046

GS ~

Amco2 0.091 0.001 -0.071 0.044 -0.000 -0.045

intraco2 ~

Amco2 0.529 0.369 0.462 0.044 0.170 0.066

rubisco ~

temp -0.062 0.008 -1.165 0.044 -0.009 -0.025

intraco2 0.142 0.000 -9.006 0.000 -0.014 -0.827

toc ~

pn 1.216 0.262 -2.081 0.037 -0.546 -0.282

GS ~

temp 0.122 0.002 -1.220 0.022 -0.002 -0.171

sd 0.063 0.000 0.294 0.068 0.000 0.041

pn ~

GS 0.750 2.242 -1.420 0.056 7.380 0.076

GS ~

sm 0.001 0.006 -0.141 0.088 -0.001 -0.089

intraco2 ~

GS 1.600 240.170 0.528 0.098 126.805 0.075

Variances:

Estimate Std.Err z-value P(>|z|) Std.lv Std.all

.chla 0.290 0.058 5.000 0.000 0.290 0.985

.rubisco 0.004 0.001 5.000 0.000 0.004 0.022

.pn 1.460 0.292 5.000 0.000 1.460 0.135

.GS 0.000 0.000 5.000 0.000 0.000 0.955

.intraco2 383.059 116.612 5.000 0.000 383.059 0.991

.toc 37.146 7.429 5.000 0.000 37.146 0.920

R-Square:

Estimate

chla 0.015

rubisco 0.978

pn 0.865

GS 0.045

intraco2 0.009

toc 0.580

**Table S6.** Mean diameter, wood density and height of tree species in Sacred Groves Stands and *Prosopis* *juliflora* Stands across the study area

| **Species** | **DBH (cm)** | **Wood Density (g/cm^3^)** | **Height (m)** |
| --- | --- | --- | --- |
| *Alangium_salviifolium* | 17 | 0.86 | 5.49 |
| *Morinda_citrifolia* | 7 | 0.64 | 2.74 |
| *Tamarindus_indica* | 32 | 0.43 | 3.66 |
| *Ailanthus_excelsa* | 14 | 0.80 | 3.96 |
| *Prosopis_juliflora* | 19 | 0.29 | 3.05 |
| *Acacia_auriculiformis* | 7 | 0.48 | 4.27 |
| *Albizia_amara* | 9 | 0.19 | 1.52 |
| *Albizia_lebbeck* | 13 | 0.35 | 5.79 |
| *Atalantia_monophylla* | 19 | 0.72 | 1.22 |
| *Azadirachta_indica* | 19 | 0.46 | 9.14 |
| *Bauhinia_racemosa* | 16 | 0.45 | 4.27 |
| *Borassus_flabellifer* | 15 | 0.68 | 2.13 |
| *Cassia_fistula* | 12 | 0.53 | 10.6 |
| *Cassia_roxburghii* | 9 | 0.54 | 7.01 |
| *Chloroxylon_swietenia* | 7 | 0.30 | 6.10 |
| *Commiphora_caudata* | 29 | 0.47 | 2.44 |
| *Crateva_adansonii* | 24 | 0.37 | 7.62 |
| *Diospyros_ebenum* | 17 | 0.58 | 9.14 |
| *Diospyros_montana* | 34 | 0.79 | 5.91 |
| *Dolichandrone_falcata* | 12 | 0.63 | 4.88 |
| *Drypetes_sepiaria* | 19 | 0.41 | 6.71 |
| *Euphorbia_antiquorum* | 7 | 0.25 | 4.57 |
| *Falconeria_insignis* | 17 | 0.63 | 8.53 |
| *Ficus_hispida* | 16 | 0.65 | 3.05 |
| *Ficus_microcarpa* | 21 | 0.26 | 8.23 |
| *Ficus_religiosa* | 15 | 0.74 | 10.0 |
| *Ficus_benghalensis* | 14 | 0.77 | 7.92 |
| *Gmelina_arborea* | 33 | 0.55 | 2.13 |
| *Grewia_tiliifolia* | 45 | 0.27 | 2.44 |
| *Holoptelea_integrifolia* | 15 | 0.63 | 9.14 |
| *Lannea_coromandelica* | 26 | 0.28 | 0.91 |
| *Acacia_nilotica* | 14 | 0.82 | 6.10 |
| *Mallotus_nudiflorus* | 15 | 0.64 | 7.01 |
| *Mangifera_indica* | 15 | 0.51 | 7.92 |
| *Mimusops_elengi* | 7 | 0.69 | 8.84 |
| *Mitragyna_parvifolia* | 6 | 0.87 | 8.23 |
| *Morinda_coreia* | 18 | 0.28 | 8.53 |
| *Nothopegia_colebrookeana* | 7 | 0.38 | 3.35 |
| *Pleiospermium_alatum* | 12 | 0.61 | 5.79 |
| *Pongamia_pinnata* | 18 | 0.23 | 7.92 |
| *Psydrax_umbellatus* | 16 | 0.26 | 9.14 |
| *Pterospermum_suberifolium* | 7 | 0.58 | 9.14 |
| *Sapindus_emarginatus* | 8 | 0.22 | 5.79 |
| *Strychnos_nuxvomica* | 7 | 0.65 | 5.49 |
| *Terminalia_arjuna* | 20 | 0.48 | 7.32 |
| *Thespesia_populnea* | 18 | 0.65 | 5.00 |
| *Vachellia_leucophloea* | 26 | 0.23 | 7.62 |
| *Wrightia_tinctoria* | 22 | 0.83 | 8.53 |
| *Ziziphus_jujuba* | 32 | 0.74 | 5.79 |

***Mapping soil erosion using the RUSLE technique***

The study used the Revised Universal Soil Loss Equation (RUSLE) in conjunction with ArcGIS Pro and ArcGIS 10.3 to map soil loss. For estimating the soil erosion, all the datasets were terminated using raster analysis. The factors had the same projected coordinates system (WGS1984/UTM 44N). Five factors were considered for soil loss estimation: rainfall erosivity, soil erodibility, slope steepness and length, crop management factor, and support practice for the RUSLE model. Datasets were prepared at varying resolutions. The factors varied spatially and temporally, dependent on other factors. Asters DEM at 15m resolution, ESRI land cover sentinel-2 dataset, FAO/ UNESCO soil map and CHRS Persiann with a resolution of 0.25° x 0.25° were used to compute the factors in ArcGIS Pro and ArcGIS 10.3. This method is based on quantitative analysis using the analytical tools of ArcGIS to assess the statistical properties of rainfall, Digital Elevation Model (DEM), and to estimate soil erosion. All the datasets were fused and integrated with remote sensing and GIS to model the Revised Universal Soil Loss Equation (RUSLE). The RUSLE model is commonly used for sheet and rill erosion, influenced by land use patterns.

**A = R*LS*P*C*K** (1)

*A* is the average of soil loss, *R* is the rainfall erosivity factor [MJ mm ha^−1^ h^−1^ year^−1^)]; *K* is the soil erodibility factor [t ha h ha^−1^MJ^−1^ mm^−1^)]; *L* is the slope length factor; *S* is the slope steepness factor; *C* is the cover and management factor, and *P* is the conservation support-practices factor. The above equation is used to estimate the average soil loss for a long period. This equation is calculated in ArcGIS 10.3 using Map algebra to prepare the soil loss map. The other research works have proved that these parameters (R, K, S, C, L and P) of the RUSLE model can be attained, but validating those sources is vital in the result. Therefore, an Accuracy assessment was done to check the accuracy of the map and reduce the error; thus, Kappa index, user accuracy, producer accuracy and overall accuracy indices were used to validate the result of soil erosion.

**Table S7.** Types of data utilized in the current study and their respective description sources.

| **Data type** | **Source** | **Description** | **Resolution** |
| --- | --- | --- | --- |
| Soil map | FAO | Soil profile | Vector data |
| Land use land cover | ESRI Land cover | Land cover using sentinel -2 | 10m |
| Rainfall | CHRS | Persiann | 27.75 km |
| Terrain | Aster DEM | Topographic map | 15m |

***R- Factor Rainfall Erosivity***

R factor quantifies the effect of the rain droplets' impact on soil regime, the kinetic energy of raindrops consequently forming a sheet of water leading to rill erosion. The R-factor is usually expressed in units of MJ/mm/ha/h^-1^/yr^-1,^ which shows the amount of energy per unit area/time leading to soil erosion; the higher the rainfall erosivity greater the probability for soil erosion. During the current study, the highest rainfall recorded in 2022 ranged from 286 mm to 880 mm across the study area. The highest rainfall was recorded in the study area's northeastern part (Ariyalur), with a mean of 349.3 mm. The rainfall gradually decreased from northeastern to southwestern areas. Dindigul, Pudukkottai and Tiruchirappalli recorded moderate and below moderate rainfall, indicating less possibility of eroding soil. Ariyalur and Perambalur have high rainfall erosivity that accelerates soil erosion.

R = 0.562 *P -8.12 (2)

Where R is the rainfall erosivity (MJ/mm/ha/hr^-1^/yr^-1^) and P is the mean Annual Rainfall (mm)
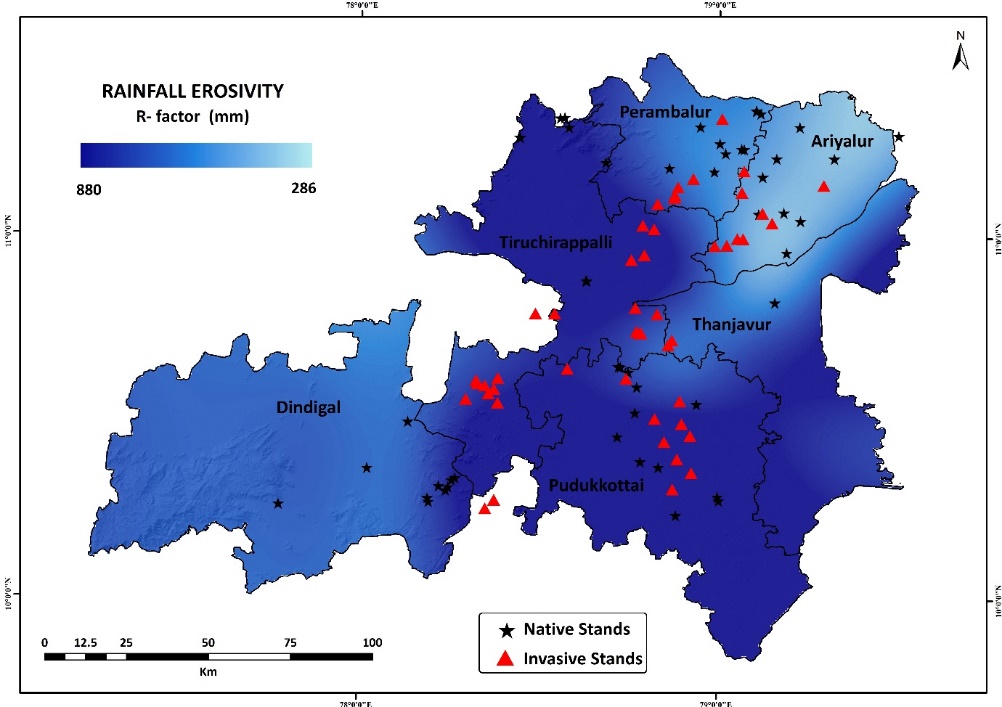


**Fig S7.** Rainfall Erosivity in the study region at Central Districts of Tamil Nadu

***K Factor Soil erodibility***

The Soil Erodibility Factor (K) determines a surface material or soil's vulnerability to erosion, sediment transportability, and runoff rate, measured under standard conditions using a unit plot with a 9% gradient and 22.6 meters length, tilled up and down the hill and maintained as continuous fallow influenced by soil texture, profile structure, organic matter content, and permeability ^160^. Its values range from 0.01 for stable soils to 0.70 for fragile soils ^161^, indicating the rate of soil loss based on the rainfall erosivity (R) index. A study, focusing on the Chilika watershed, established a relationship between organic matter and soil texture to employ the soil erodibility factor ^150^. The current study used the following equation, developed by Williams ^162^, to determine the soil erodibility factor (K).

**K_RUSLE_ = K_w_ = F_csand_ * F_cl-si_ * F_orgc_ * F_hisand_**  (3)

**^F^_csand_** is the factor that lowers the K indicator in soil with high coarse sand content and higher for soil with little sand.

**^F^_Ci-Si is_** the low soil erodibility factor for the soil with high clay-to-slit- ratios.

F**_orgnc_** reduce the K value in soil with high organic content

**F_hisand_** Lowers the K content of the soil with extremely high sand content

**F_csand_ = (**0.2+0.3*exp [-0.256*m_s_*(1-m_silt_ ÷ 100)]**)** (1)

**F_cl-si_ = (**m_silt_ **÷** m_c_ + m_silt_**)**^0.3^  (2)

**F**_orgc_ **= (**1-0.25*orgc **÷** orgc + exp [3.72-2.95*orgc]**)** (3)

**F**_hisand_ **= [** 1- 0.7*(1-m_silt_ **÷** 100) **÷** (1-m_silt_ **÷** 100) +exp [-5.51+22.9*(1-m_silt_ **÷** 100)] **]** (4)

M_s_ is the sand fraction content (0.05-2.00mm diameter) [%]

M_slit_ is the slit fraction content (0.002 – 0.05 mm diameter) [%]

M_c_ is the clay fraction content (<0.002 mm diameter) [%]

orgC is the organic carbon (SOC) content [%]

| Soil unit symbol | sand % topsoil | silt % topsoil | clay % topsoil | OC % topsoil | F_csand_ | F_cl-si_ | F_orgc_ | F_hisand_ | K- Factor |  |
| --- | --- | --- | --- | --- | --- | --- | --- | --- | --- | --- |
| LC | 64.3 | 12.2 | 23.5 | 0.63 | 0.2 | 0.724615 | 0.997717 | 0.983034 | 0.142139 |  |
| VP | 25.1 | 12.2 | 62.7 | 0.68 | 0.201064 | 0.580181 | 0.997206 | 0.999995 | 0.116327 |  |
| I | 58.9 | 16.2 | 24.9 | 0.97 | 0.200001 | 0.756314 | 0.992542 | 0.994235 | 0.14927 |  |
| JE | 70.8 | 12.8 | 16.5 | 1.15 | 0.2 | 0.780015 | 0.988398 | 0.942194 | 0.14528 |  |
| AP | 57 | 15.6 | 27.1 | 1.09 | 0.200001 | 0.73928 | 0.989839 | 0.996086 | 0.145782 |  |
| LO | 76 | 9.9 | 14.1 | 0.41 | 0.2 | 0.766703 | 0.999175 | 0.86298 | 0.132221 |  |
| ND | 38.9 | 17.6 | 43.6 | 1.57 | 0.200082 | 0.688063 | 0.979618 | 0.999911 | 0.134851 |  |
| VC | 22.4 | 24.5 | 53 | 0.69 | 0.203952 | 0.707879 | 0.997095 | 0.999997 | 0.143954 |  |
| RD | 82.1 | 6.7 | 11.3 | 0.27 | 0.2 | 0.743431 | 0.999634 | 0.703729 | 0.104596 |  |
| AH | 31.3 | 24.8 | 43.8 | 3.34 | 0.200725 | 0.73695 | 0.974417 | 0.999983 | 0.144137 |  |

**Table S8.** Percentage of topsoil and k factor

**Table S9.** Soil type’s abbreviation

| **Soil Symbol** | **Abbreviation** |
| --- | --- |
| C | Chromic Luvisols |
| VP | Pellic Vertisols |
| I | Inceptisol |
| JE | Eutric Fluvisols |
| AP | Plinthic Acrisols |
| LO | Orthic Luvisols |
| ND | Dystric Nitosols |
| VC | Chromic Vertisols |
| RD | Dystric Regosols |
| AH | Humic Acrisols |


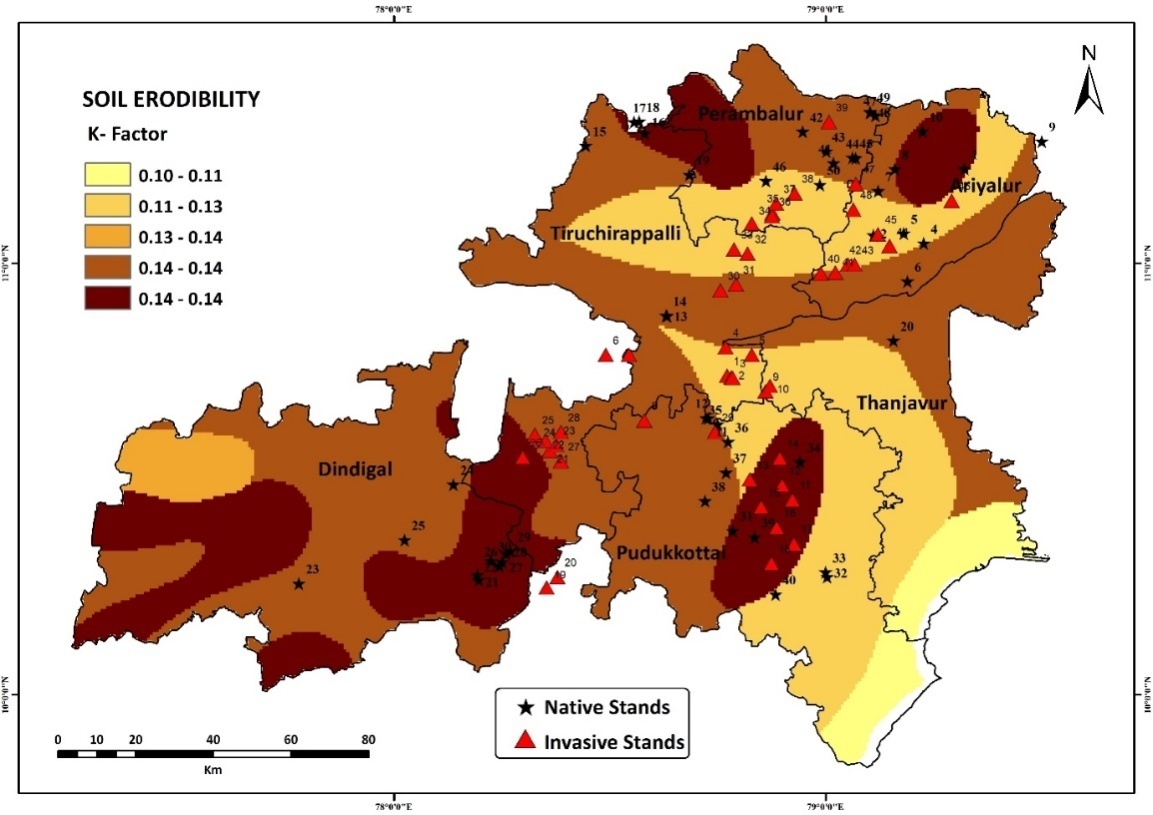


**Fig S8.** Soil Erodibility across the study region in central districts of Tamil Nadu

***Topographic or Slope Length/Steepness Factor (LS)***

Topography is an important factor in the RUSLE model to determine soil erosion. Slope length is the distance from the origin of overland flow along its path to the location of concentrated flow or deposition. The topographic factor comprises the elements influencing soil erosion through slope length and steepness; slope length and soil erosion are positively correlated. LS factor is a combination of fill, flow accumulation, flow direction, and slope contribute area per contour. In the current study, 80% of the study area is a low undulated terrain; in the northern and western parts, Small Mountain contributes to the overall steepness of the study area. Therefore, it is concluded that the study shows a very low steepness and length, resulting in a decline in soil loss over the area. LS factor for the present study was derived by slope and flow accumulation ^163^ as follows:

**LS= pow [(Flow accumulation)*cell size/22.13]0.4*pow [sin (slope*0.01745/0.0896]1.3** (4)


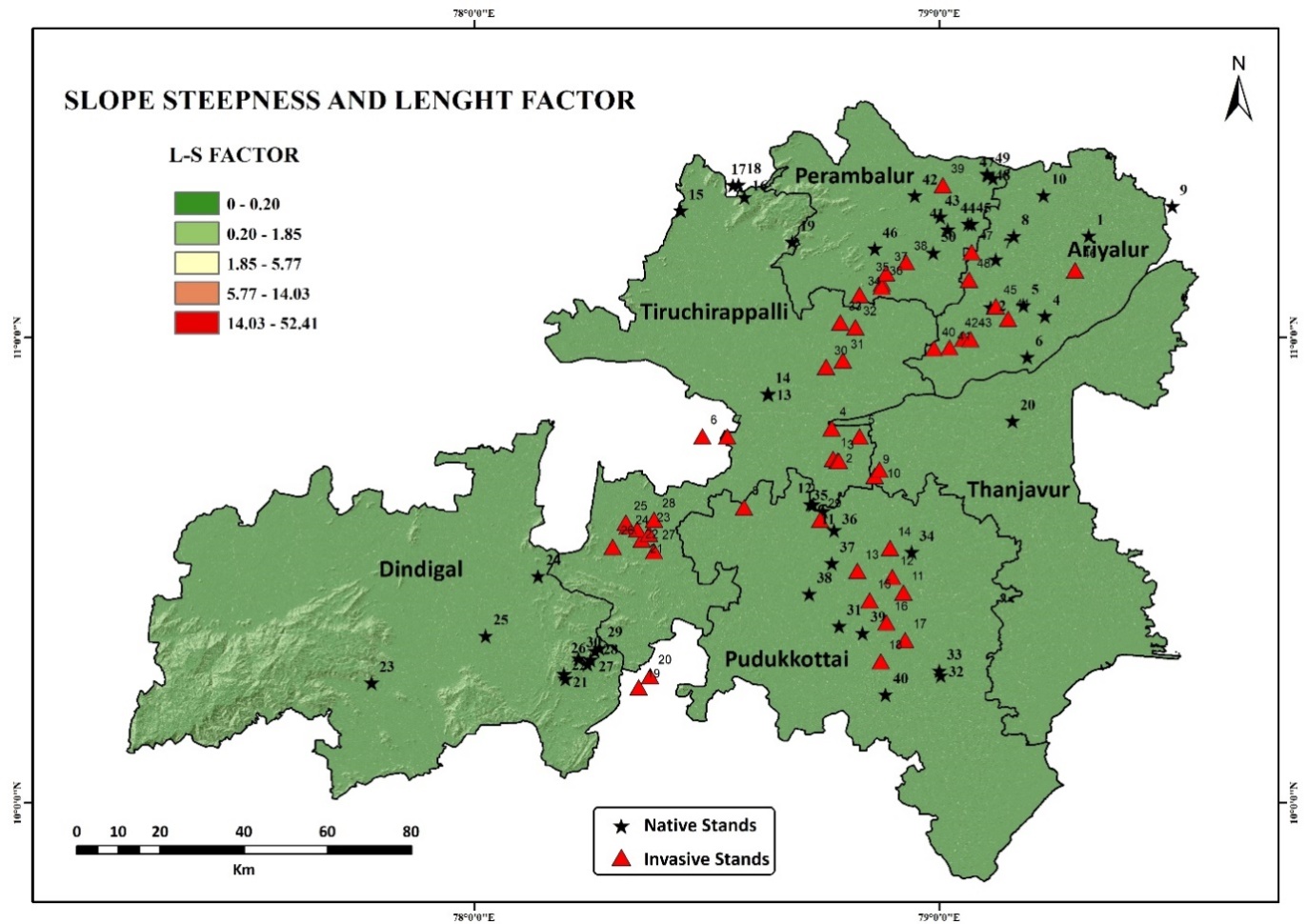


**Fig S9**. Slope steepness and length map of Central Districts of Tamil Nadu

***P-Factor support practice factor***

P-factor is associated with contouring, strip cropping, terracing and other activities by which land can be managed to stop soil erosion. P-value was assessed using land use land cover ^29^ ; due to the high influence of land use land cover in the study area ^168^. The effectiveness of erosion control practices can vary depending on land management practices and other local conditions. The value of the P factor is in the range of 0 to 1, where 0 represents complete vegetation cover and the highly effective control of soil erosion, and 1 represents bare soil with high soil erosion. The P-factor is attributed to the results of the overall management of the area through various measurements and land management practices. The P-factor was generated based on land use types in each land use class (Table S10).

**Table S10.** Support Practice factor

| **LULC class** | **P – factor value** | **Sources** |
| --- | --- | --- |
| Settlement | 1.0 | ^167^ |
| Barren land | 1.0 | ^167^ |
| Forest | 0.8 (0.4) |  |
| Water bodies | 1.0 | ^167^ |
| Wetland | 1.0 | ^167^ |
| Cropland | 0.5 (0.7) |  |
| Scrubland | 1.0 | ^167^ |


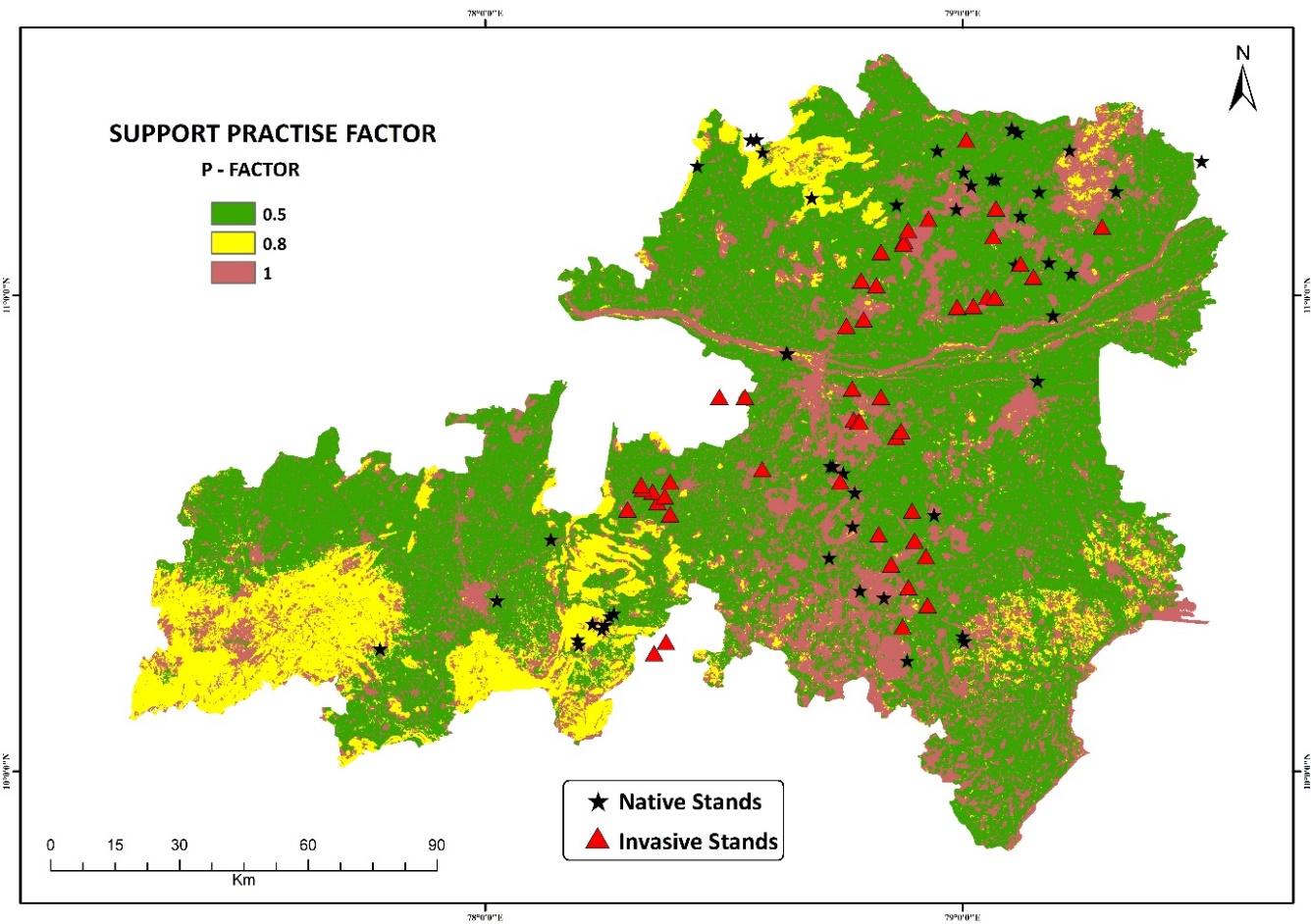


**Fig S10.** Conservation support practice factor map in central districts of Tamil Nadu.

***C- factor cover management***

The C-factor plays a crucial role in crop management; it is to be noted that when there is a loss of vegetation or uncovered vegetation, there is a chance for high soil erosion. To overcome this limitation, Karaburun^164^ calculated C-factor values, which were then utilized to assess the impact of management and cropping patterns on soil erosion rates and amounts in India's agricultural lands. Vegetation type and its quality, as well as management practices, significantly influence soil erosion in any region ^165,166^


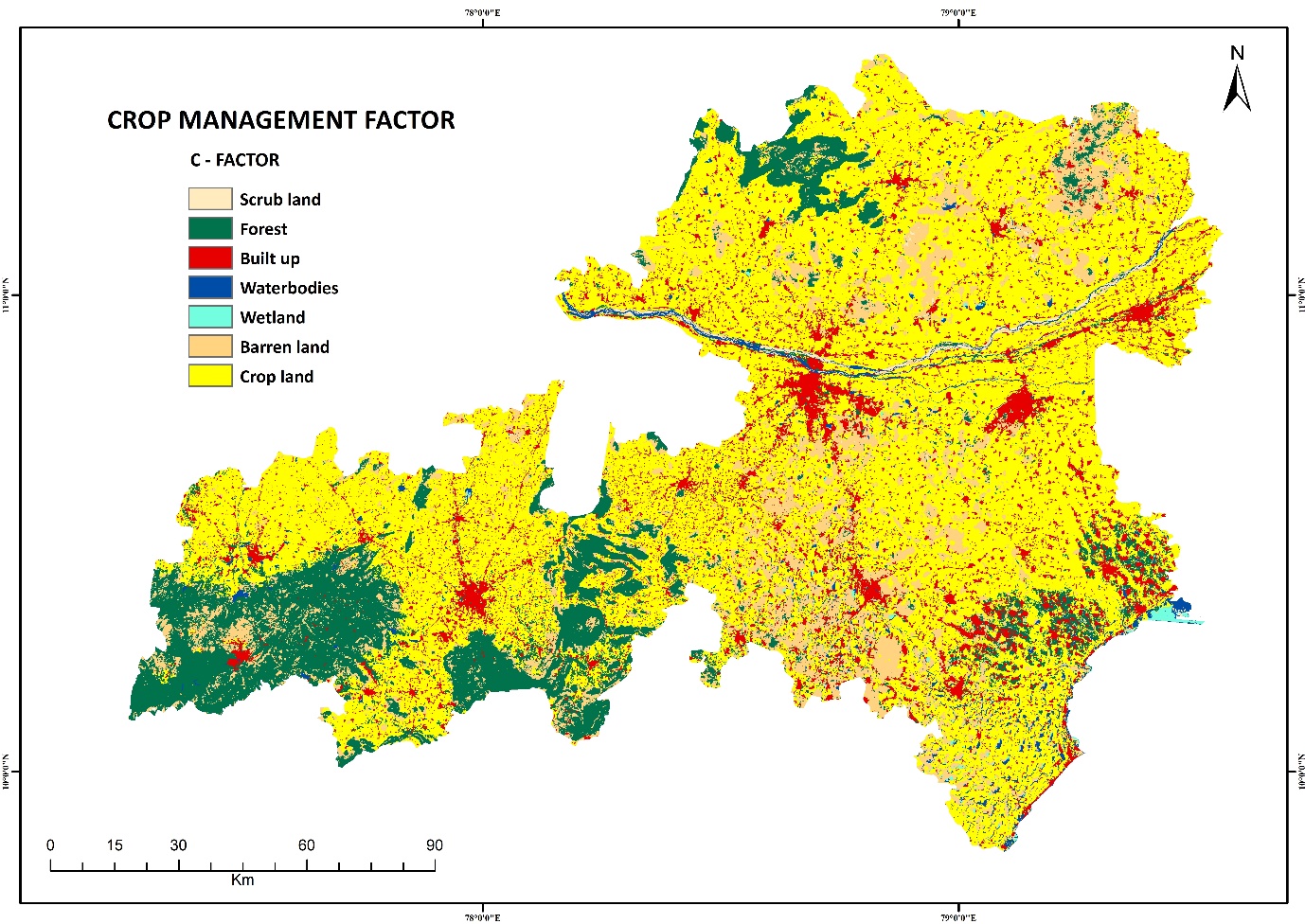


**Fig S11.** Land use Land cover map of central districts of Tamil Nadu

**Table. S11.** Crop management factor values for different land use.

| **LULC class** | **C- factor value** | **Sources** |
| --- | --- | --- |
| Settlement | 0.09 | ^167^ |
| Barren land | 0.50 | ^167^ |
| Forest | 0.003 | ^167^ |
| Water bodies | 0.28 |  |
| Wetland | 0.28 | ^167^ |
| Cropland | 0.63 |  |
| Scrubland | 0.001 | ^167^ |

**Table S12.** Land Use Land Cover area and its proportions across the study region (2022)

| **LULC** | **Area (Sq.km)** | **Area (%)** |
| --- | --- | --- |
| Water Bodies | 469.11 | 2.09 |
| Forest | 3150.68 | 14.5 |
| Flooded vegetation | 9.70 | 0.22 |
| Crop | 13688.58 | 61.07 |
| Built up | 2325.34 | 10.37 |
| Grassland | 105.20 | 0.46 |
| Barren land | 2574.64 | 11.29 |

**Table S13.** Statistical analysis of RUSLE model factors

| **Category** | **K factor** | **R factor** | **LS factor** | **C factor** | **P factor** |
| --- | --- | --- | --- | --- | --- |
| Maximum | 0.14 | 880 | 52.14 | 0.63 | 1.0 |
| Mean | 0.13 | 406 | 0.02 | 0.4 | 0.6 |
| Minimum | 0.10 | 286 | 0.0 | 0.001 | 0.5 |

***Soil loss estimation***

All across the study area, soil erosion was very high in montane regions and generally low in plains. The erosion rate encountered in the montane region was 84.26 ton/ha/year, which is relatively very high. In Invasive Stands, the mean soil loss/erosion estimated was 4.52 ± 2.55 ton/ha/year; in contrast, soil erosion under Native Stands was 33.5 ± 13.4 ton/ha/year, higher than in Invasive Stands. Low soil erosion in Invasive Stands might be attributed to the very high depositional rate and Soil Organic Carbon enrichment trait of *Prosopis* *juliflora*; organic exudates from roots create a highly adhesive humus layer, which in turn holds the soil and prevent erosion. Though the bulk densities of the under-canopy soil in Invasive stands were low compared to Native Stands, soil erosion rates were low in Invasive Stands across the study region. In montane regions, Native Stands tend to decrease soil erosion and increase land stability in multiple folds, but in open montane areas, a very high erosion rate was observed due to the higher slope and rainfall.

**Table S14.** Soil erosion proportions across the study area.

| **Soil erosion classes** | **Soil loss**  **(ton/ha/year)** | **Area (ha)** | **Area (%)** |
| --- | --- | --- | --- |
| Very low | 0.30 - 6.60 | 15,69,120 | 72 |
| Low | 6.60 - 9.90 | 2,68,992 | 12 |
| Moderate | 9.90 - 16.5 | 2,01,744 | 9 |
| High | 16.5 - 33.0 | 89,664 | 4 |
| Very high | 33.0 - 84.2 | 67,248 | 3 |

**Table. S15.** Kappa coefficient validating RUSLE model Accuracy totals for LULC across the study area

| **Class name** | **Reference total** | **Classified total** | **Number correct** | **Percent accuracy** | | **Overall accuracy (%)** | **Kappa coefficient**  **(%)** |
| --- | --- | --- | --- | --- | --- | --- | --- |
|  |  |  |  | **Producer accuracy (%)** | **User accuracy (%)** |  |  |
| Water bodies | 16 | 13 | 3 | 100 | 91.25 | 91.76 | 92.21 |
| Forests | 67 | 56 | 11 | 82.3 | 93.5 |  |  |
| Flooded vegetation | 03 | 03 | 0 | 89 | 100 |  |  |
| Cropland | 52 | 47 | 5 | 94.12 | 90.3 |  |  |
| Built up | 47 | 43 | 4 | 97.7 | 91.4 |  |  |
| Barren land | 58 | 42 | 16 | 89.5 | 82.1 |  |  |
| **Total** | 243 | 204 | 39 |  |  |  |  |

**Reference**:

1. Gadgil, M. & Vartak, V. D. The sacred groves of Western Ghats in India. *Econ. Bot.* **30**, 152–160 (1976).

2. Chandran, M. D. S. & Hughes, J. D. The sacred groves of south India: ecology, traditional communities and religious change. *Soc. compass* **44**, 413–427 (1997).

3. Chandran, M. D. S. & Mesta, D. K. On the conservation of the Myristica swamps of the Western Ghats. *For. Genet. Resour. status, Threat. Conserv. Strateg.* 1–19 (2001).

4. Douglass, K. & Zinke, J. Forging ahead by land and by sea: Archaeology and paleoclimate reconstruction in Madagascar. *African Archaeol. Rev.* **32**, 267–299 (2015).

5. Devi, N. B., Lepcha, N. T., Mahalik, S. S., Dutta, D. & Tsanglao, B. L. Urban sacred grove forests are potential carbon stores: A case study from Sikkim Himalaya. *Environ. Challenges* **4**, 100072 (2021).

6. Nganso, T. B., Kyerematen, R. & Obeng-Ofori, D. Review of biodiversity in sacred groves in Ghana and implications on conservation. *Curr. Trends Ecol.* **3**, 1–10 (2012).

7. Cardelus, C. L. *et al.* Shadow conservation and the persistence of sacred church forests in northern Ethiopia. *Biotropica* **49**, 726–733 (2017).

8. Ormsby, A. A. & Bhagwat, S. A. Sacred forests of India: a strong tradition of community-based natural resource management. *Environ. Conserv.* **37**, 320–326 (2010).

9. Wadley, R. L. & Colfer, C. J. P. Sacred forest, hunting, and conservation in West Kalimantan, Indonesia. *Hum. Ecol.* **32**, 313–338 (2004).

10. Bhagwat, S. A., Kushalappa, C. G., Williams, P. H. & Brown, N. D. A landscape approach to biodiversity conservation of sacred groves in the Western Ghats of India. *Conserv. Biol.* **19**, 1853–1862 (2005).

11. Mequanint, F. *et al.* Biodiversity conservation in the sacred groves of north-west Ethiopia: diversity and community structure of woody species. *Glob. Ecol. Conserv.* **24**, e01377 (2020).

12. Kibet, S. & Nyamweru, C. Cultural and biological heritage at risk; the case of the Rabai Kaya forests in Coastal Kenya. *J. Hum. Ecol.* **24**, 287–295 (2008).

13. Bhagwat, S. A., Nogué, S. & Willis, K. J. Cultural drivers of reforestation in tropical forest groves of the Western Ghats of India. *For. Ecol. Manage.* **329**, 393–400 (2014).

14. Chandrashekara, U. M. & Sankar, S. Ecology and management of sacred groves in Kerala, India. *For. Ecol. Manage.* **112**, 165–177 (1998).

15. Balvanera, P. & Cotler, H. Acercamientos al estudio de los servicios ecosistémicos. *Gac. ecológica* 8–15 (2007).

16. Newbold, T. *et al.* Global effects of land use on local terrestrial biodiversity. *Nature* **520**, 45–50 (2015).

17. Calzadilla, P. I., Carvalho, F. E. L., Gomez, R., Lima Neto, M. C. & Signorelli, S. Assessing photosynthesis in plant systems: A cornerstone to aid in the selection of resistant and productive crops. *Environ. Exp. Bot.* **201**, 104950 (2022).

18. Gaudiano, L. *et al.* Spatio-temporal behaviour of female wild boar in an agro-forestry–pastoral landscape of Southern Italy. *Mammal Res.* **67**, 163–172 (2022).

19. Li-Ping, G., Lan-Zhu, J., Wei-Dong, Z., Yue, Z. & Jun-Gang, X. Forest recovery state in wind disaster area of Changbai Mountains, Northeast China. *Yingyong Shengtai Xuebao* **21**, (2010).

20. MEa, M. E. A. Ecosystems and Human Well-Being: wetlands and water synthesis. (2005).

21. Hernández-Morcillo, M., Plieninger, T. & Bieling, C. An empirical review of cultural ecosystem service indicators. *Ecol. Indic.* **29**, 434–444 (2013).

22. Dearborn, D. C. & Kark, S. Motivations for conserving urban biodiversity. *Conserv. Biol.* **24**, 432–440 (2010).

23. Castro‐Díez, P. *et al.* Global effects of non‐native tree species on multiple ecosystem services. *Biol. Rev.* **94**, 1477–1501 (2019).

24. Valentine, K. *et al.* Climate-driven tradeoffs between landscape connectivity and the maintenance of the coastal carbon sink. *Nat. Commun.* **14**, 1137 (2023).

25. Heinrich, V. H. A. *et al.* The carbon sink of secondary and degraded humid tropical forests. *Nature* **615**, 436–442 (2023).

26. Trugman, A. T. *et al.* Climate and plant trait strategies determine tree carbon allocation to leaves and mediate future forest productivity. *Glob. Chang. Biol.* **25**, 3395–3405 (2019).

27. Malsatar, A. & Mehta, P. K. Sacred Groves: A Novice Idea for Carbon Sequestration. *J. Plant Sci. Res.* **39**, (2023).

28. Ke, S., Zhang, Z. & Wang, Y. China’s forest carbon sinks and mitigation potential from carbon sequestration trading perspective. *Ecol. Indic.* **148**, 110054 (2023).

29. Larson, W. E., Lindstrom, M. J. & Schumacher, T. E. The role of severe storms in soil erosion: a problem needing consideration. *J. Soil Water Conserv.* **52**, 90–95 (1997).

30. Parthasarathy, N. & Naveen Babu, K. Sacred Groves: Potential for Biodiversity and Bioresource Management. 865–880 (2021) doi:10.1007/978-3-319-95981-8_10.

31. WITT, A. B. R. Biofuels and invasive species from an African perspective - a review. *GCB Bioenergy* **2**, 321–329 (2010).

32. Ye, Q. *et al.* Dissolved organic matter characteristics in soils of tropical legume and non-legume tree plantations. *Soil Biol. Biochem.* **148**, 107880 (2020).

33. Brewer, M. J. & Elliott, N. C. Recent advances in agroecological research for increasing scope of areawide pest management of arthropods in cropping systems. *Curr. Opin. Insect Sci.* 101019 (2023).

34. Ormsby, A. A. Diverse values and benefits of urban sacred natural sites. *Trees, For. people* **6**, 100136 (2021).

35. Dar, A. A. & Parthasarathy, N. Ecological drivers of soil carbon in Kashmir Himalayan forests: Application of machine learning combined with structural equation modelling. *J. Environ. Manage.* **330**, 117147 (2023).

36. Dar, J. A. *et al.* Tree diversity, biomass and carbon storage in sacred groves of Central India. *Environ. Sci. Pollut. Res.* **26**, 37212–37227 (2019).

37. Mgumia, F. H. & Oba, G. Potential role of sacred groves in biodiversity conservation in Tanzania. *Environ. Conserv.* **30**, 259–265 (2003).

38. Ray, R., Chandran, M. D. S. & Ramachandra, T. V. Biodiversity and ecological assessments of Indian sacred groves. *J. For. Res.* **25**, 21–28 (2014).

39. Kumar, P. *et al.* Carbon sequestration and soil carbon build-up under Eucalyptus plantation in semi-arid regions of North-West India. *J. Sustain. For.* **40**, 319–331 (2021).

40. Yadav, V. S. *et al.* Carbon sequestration potential and CO2 fluxes in a tropical forest ecosystem. *Ecol. Eng.* **176**, 106541 (2022).

41. Ramanujam, M. P. & Praveen Kumar Cyril, K. Woody species diversity of four sacred groves in the Pondicherry region of South India. *Biodivers. Conserv.* **12**, 289–299 (2003).

42. Devi Khumbongmayum, A., Khan, M. L. & Tripathi, R. S. Sacred groves of Manipur, northeast India: biodiversity value, status and strategies for their conservation. *Biodivers. Conserv.* **14**, 1541–1582 (2005).

43. Kandari, L. S., Bisht, V. K., Bhardwaj, M. & Thakur, A. K. Conservation and management of sacred groves, myths and beliefs of tribal communities: a case study from north-India. *Environ. Syst. Res.* **3**, 1–10 (2014).

44. Meena, R. S., Yadav, A., Kumar, S., Jhariya, M. K. & Jatav, S. S. Agriculture ecosystem models for CO2 sequestration, improving soil physicochemical properties, and restoring degraded land. *Ecol. Eng.* **176**, 106546 (2022).

45. Oduor, N. M. & Githiomi, J. K. Fuel-wood energy properties of Prosopis juliflora and Prosopis pallida grown in Baringo District, Kenya. *African J. Agric. Res.* **8**, 2476–2481 (2013).

46. Dey, A. *et al.* Effect of conservation agriculture on soil organic and inorganic carbon sequestration and lability: A study from a rice–wheat cropping system on a calcareous soil of the eastern Indo‐Gangetic Plains. *Soil Use Manag.* **36**, 429–438 (2020).

47. Ayanu, Y. *et al.* Ecosystem engineer unleashed: Prosopis juliflora threatening ecosystem services? *Reg. Environ. Chang.* **15**, 155–167 (2015).

48. Elfadl, M. A. & Luukkanen, O. Field studies on the ecological strategies of Prosopis juliflora in a dryland ecosystem: 1. A leaf gas exchange approach. *J. Arid Environ.* **66**, 1–15 (2006).

49. Garg, V. K. Interaction of tree crops with a sodic soil environment: Potential for rehabilitation of degraded environments. *L. Degrad. Dev.* **9**, 81–93 (1998).

50. Tiessen, H., Menezes, R. S. C., Salcedo, I. H. & Wick, B. Organic matter transformations and soil fertility in a treed pasture in semiarid NE Brazil. *Plant Soil* **252**, 195–205 (2003).

51. Dar, J. A. *et al.* Tree diversity, biomass and carbon storage in sacred groves of Central India. *Environ. Sci. Pollut. Res.* **26**, 37212–37227 (2019).

52. Oliveira, M. T. *et al.* Seasonal variability in physiological and anatomical traits contributes to invasion success of Prosopis juliflora in tropical dry forest. *Tree Physiol.* **37**, 326–337 (2017).

53. Iftikhar Hussain, M., El-Keblawy, A. & Tsombou, F. M. Leaf age, canopy position, and habitat affect the carbon isotope discrimination and water-use effciency in three c3 leguminous prosopis species from a hyper-arid climate. *Plants* **8**, 1–11 (2019).

54. Quijas, S., Schmid, B. & Balvanera, P. Plant diversity enhances provision of ecosystem services: A new synthesis. *Basic Appl. Ecol.* **11**, 582–593 (2010).

55. Mace, G. M., Norris, K. & Fitter, A. H. Biodiversity and ecosystem services: A multilayered relationship. *Trends Ecol. Evol.* **27**, 19–26 (2012).

56. Edrisi, S. A., El-Keblawy, A. & Abhilash, P. C. Sustainability analysis of Prosopis juliflora (Sw.) DC based restoration of degraded land in North India. *Land* **9**, (2020).

57. Leakey, R. R. B. *et al.* The Future of Food: Domestication and Commercialization of Indigenous Food Crops in Africa over the Third Decade (2012–2021). *Sustain.* **14**, (2022).

58. Mukherjee, A., Velankar, A. D. & Kumara, H. N. Invasive Prosopis juliflora replacing the Native Floral Community over three decades: a case study of a World Heritage Site, Keoladeo National Park, India. *Biodivers. Conserv.* **26**, 2839–2856 (2017).

59. Kumar, S. & Mathur, M. Invasion of Prosopis juliflora in native arid grazing lands: Competition and dominance. *Range Manag. Agrofor.* **33**, 162–165 (2012).

60. Holmgren, M. *The Prosopis juliflora–Prosopis pallida Complex: A Monograph*. *Forest Ecology and Management* vol. 174 (HDRA Coventry, 2003).

61. Tomar, O. S., Minhas, P. S., Sharma, V. K., Singh, Y. P. & Gupta, R. K. Performance of 31 tree species and soil conditions in a plantation established with saline irrigation. *For. Ecol. Manage.* **177**, 333–346 (2003).

62. Joshi, R. K. & Garkoti, S. C. Litter dynamics, leaf area index and forest floor respiration as indicators for understanding the role of Nepalese alder in white oak forests in central Himalaya, India. *Ecol. Indic.* **111**, 106065 (2020).

63. Singh, A. K., Sisodia, A., Sisodia, V. & Padhi, M. *Role of microbes in restoration ecology and ecosystem services*. *New and Future Developments in Microbial Biotechnology and Bioengineering: Microbial Biotechnology in Agro-environmental Sustainability* (Elsevier B.V., 2019). doi:10.1016/B978-0-444-64191-5.00004-3.

64. de Brito Damasceno, G. A. *et al.* Prosopis juliflora: Phytochemical, Toxicological, and Allelochemicals. *Ref. Ser. Phytochem.* 521–541 (2020) doi:10.1007/978-3-319-96397-6_15.

65. Wakie, T. T., Laituri, M. & Evangelista, P. H. Assessing the distribution and impacts of Prosopis juliflora through participatory approaches. *Appl. Geogr.* **66**, 132–143 (2016).

66. Tang, X. *et al.* Carbon pools in China’s terrestrial ecosystems: New estimates based on an intensive field survey. *Proc. Natl. Acad. Sci. U. S. A.* **115**, 4021–4026 (2018).

67. Nakano, H. *et al.* Growth inhibitory alkaloids from mesquite (Prosopis juliflora (Sw.) DC.) leaves. *Phytochemistry* **65**, 587–591 (2004).

68. Tanaka, Y., Sugano, S. S., Shimada, T. & Hara-Nishimura, I. Enhancement of leaf photosynthetic capacity through increased stomatal density in Arabidopsis. *New Phytol.* **198**, 757–764 (2013).

69. Boudell, J. A. Ecosystem services. *Wetl. B. I Struct. Funct. Manag. Methods* 121–123 (2018) doi:10.1007/978-90-481-9659-3_94.

70. Negi, A. J. D. S., Manhas, R. K. & Chauhan, P. S. Carbon allocation in different components of some tree species of India : A new approach for carbon estimation Published by : Current Science Association Carbon allocation in different components of some tree species of India : A new approach for carbon e. *Curr. Sci. Assoc.* **85**, 1528–1531 (2003).

71. Seyyed, M. S. & Haniyeh, K. Some morphological and biochemical responses due to industrial air pollution in Prosopis juliflora (Swartz) DC plant. *African J. Agric. Res.* **8**, 1968–1974 (2013).

72. Warren, C. R. & Adams, M. A. Evergreen trees do not maximize instantaneous photosynthesis. *Trends Plant Sci.* **9**, 270–274 (2004).

73. Porder, S. How Plants Enhance Weathering and How Weathering is Important to Plants. *Elements* **15**, 241–246 (2019).

74. Shiferaw, H. *et al.* Water abstraction of invasive Prosopis juliflora and native Senegalia senegal trees: A comparative study in the Great Rift Valley Area, Ethiopia. *Sci. Total Environ.* **862**, 160833 (2023).

75. Mbaabu, P. R. *et al.* Restoration of degraded grasslands, but not invasion by Prosopis juliflora, avoids trade-offs between climate change mitigation and other ecosystem services. *Sci. Rep.* **10**, 20391 (2020).

76. Tanaka, T. *et al.* Irrigation system and land use effect on surface water quality in river, at lake Dianchi, Yunnan, China. *J. Environ. Sci.* **25**, 1107–1116 (2013).

77. Hao, L. *et al.* Drought dampens the positive acclimation responses of leaf photosynthesis to elevated [CO2] by altering stomatal traits, leaf anatomy, and Rubisco gene expression in Pyrus. *Environ. Exp. Bot.* **211**, 105375 (2023).

78. Karavolias, N. G. *et al.* Paralog editing tunes rice stomatal density to maintain photosynthesis and improve drought tolerance. *Plant Physiol.* **192**, 1168–1182 (2023).

79. Schulze, E. D. Carbon dioxide and water vapor exchange in response to drought in the atmosphere and in the soil. *Annu. Rev. Plant Physiol.* **37**, 247–274 (1986).

80. Munne-Bosch, S. & Penuelas, J. Photo-and antioxidative protection, and a role for salicylic acid during drought and recovery in field-grown Phillyrea angustifolia plants. *Planta* **217**, 758–766 (2003).

81. Pei, Z.-M. & Kuchitsu, K. Early ABA signaling events in guard cells. *J. Plant Growth Regul.* **24**, 296–307 (2005).

82. Gudesblat, G. E., Iusem, N. D. & Morris, P. C. Guard cell‐specific inhibition of Arabidopsis MPK3 expression causes abnormal stomatal responses to abscisic acid and hydrogen peroxide. *New Phytol.* **173**, 713–721 (2007).

83. Xie, X. *et al.* Estimation of Leaf Area Index in a Typical Northern Tropical Secondary Monsoon Rainforest by Different Indirect Methods. *Remote Sens.* **15**, (2023).

84. Yu, T. *et al.* Interannual and seasonal relationships between photosynthesis and summer soil moisture in the Ili River basin, Xinjiang, 2000–2018. *Sci. Total Environ.* **856**, 159191 (2023).

85. Song, H. *et al.* Comparative physiological and transcriptomic analyses reveal the mechanisms of CO2 enrichment in promoting the growth and quality in Lactuca sativa. *PLoS One* **18**, e0278159 (2023).

86. Haworth, M. *et al.* The functional significance of the stomatal size to density relationship: Interaction with atmospheric [CO2] and role in plant physiological behaviour. *Sci. Total Environ.* **863**, 160908 (2023).

87. Xu, M. *et al.* Elevated CO2 aggravated polystyrene microplastics effects on the rice-soil system under field conditions. *Environ. Pollut.* **316**, 120603 (2023).

88. Yang, K. *et al.* The determiner of photosynthetic acclimation induced by biochemical limitation under elevated CO2 in japonica rice. *J. Plant Physiol.* **280**, 153889 (2023).

89. Subramanian, A. *et al.* Long-term impacts of climate change on coastal and transitional eco-systems in India: an overview of its current status, future projections, solutions, and policies. *RSC Adv.* **13**, 12204–12228 (2023).

90. Rajesh, P. V & Goswami, B. N. Climate change and potential demise of the Indian deserts. *Earth’s Futur.* **11**, e2022EF003459 (2023).

91. Ghanbari, M., Arabi, M., Georgescu, M. & Broadbent, A. M. The role of climate change and urban development on compound dry-hot extremes across US cities. *Nat. Commun.* **14**, 3509 (2023).

92. Yuan, X. *et al.* A global transition to flash droughts under climate change. *Science (80-. ).* **380**, 187–191 (2023).

93. Kominami, Y. *et al.* Biometric and eddy-covariance-based estimates of carbon balance for a warm-temperate mixed forest in Japan. *Agric. For. Meteorol.* **148**, 723–737 (2008).

94. De Souza, A. P. Dynamic responses of carbon assimilation and stomatal conductance in the future climate. *J. Exp. Bot.* **74**, 2790–2793 (2023).

95. Shirke, P. A. Leaf photosynthesis, dark respiration and fluorescence as influenced by leaf age in an evergreen tree, Prosopis juliflora. *Photosynthetica* vol. 39 305–311 (2001).

96. Mao, L. *et al.* Decreasing photosystem antenna size by inhibiting chlorophyll synthesis: A double-edged sword for photosynthetic efficiency. *Crop Environ.* **2**, 46–58 (2023).

97. Crafts-Brandner, S. J. & Salvucci, M. E. Rubisco activase constrains the photosynthetic potential of leaves at high temperature and CO2. *Proc. Natl. Acad. Sci.* **97**, 13430–13435 (2000).

98. Scafaro, A. P., Posch, B. C., Evans, J. R., Farquhar, G. D. & Atkin, O. K. Rubisco deactivation and chloroplast electron transport rates co-limit photosynthesis above optimal leaf temperature in terrestrial plants. *Nat. Commun.* **14**, 2820 (2023).

99. Feng, X., Liu, R., Li, C., Zhang, H. & Slot, M. Contrasting responses of two C4 desert shrubs to drought but consistent decoupling of photosynthesis and stomatal conductance at high temperature. *Environ. Exp. Bot.* **209**, 105295 (2023).

100. Kumar, A., Kumar, S. & Jain, N. Impact of Increasing Climatic Temperature on Crop Yield. *Mol. Biol. Plant Physiol.* **33**, 3 (2023).

101. Kaur, N. *et al.* Variation in thermotolerance of photosystem II energy trapping, intersystem electron transport, and photosystem I electron acceptor reduction for diverse cotton genotypes. *Plant Physiol. Biochem.* **201**, 107868 (2023).

102. Crafts-Brandner, S. J. & Salvucci, M. E. Analyzing the impact of high temperature and CO 2 on net photosynthesis: Biochemical mechanisms, models and genomics. *F. Crop. Res.* **90**, 75–85 (2004).

103. Lachapelle, P.-P. & Shipley, B. Interspecific prediction of photosynthetic light response curves using specific leaf mass and leaf nitrogen content: effects of differences in soil fertility and growth irradiance. *Ann. Bot.* **109**, 1149–1157 (2012).

104. Atkinson, R. R. L. *et al.* C4 photosynthesis boosts growth by altering physiology, allocation and size. *Nat. plants* **2**, 1–5 (2016).

105. Chtouki, M. *et al.* Interactive effect of soil moisture content and phosphorus fertilizer form on chickpea growth, photosynthesis, and nutrient uptake. *Sci. Rep.* **12**, 6671 (2022).

106. Nasar, J. *et al.* Nitrogen fertilization coupled with iron foliar application improves the photosynthetic characteristics, photosynthetic nitrogen use efficiency, and the related enzymes of maize crops under different planting patterns. *Front. Plant Sci.* **13**, 988055 (2022).

107. Lorimer, C. G., Dahir, S. E. & Nordheim, E. V. Tree mortality rates and longevity in mature and old‐growth hemlock‐hardwood forests. *J. Ecol.* **89**, 960–971 (2001).

108. Kirkwood, T. B. L. & Holliday, R. The evolution of ageing and longevity. *Proc. R. Soc. London. Ser. B. Biol. Sci.* **205**, 531–546 (1979).

109. Satake, A. *et al.* The molecular clock in long-lived tropical trees is independent of growth rate. *BioRxiv* 2001–2023 (2023).

110. Bauman, D. *et al.* Tropical tree mortality has increased with rising atmospheric water stress. *Nature* **608**, 528–533 (2022).

111. Ohse, B. *et al.* Demographic synthesis for global tree species conservation. *Trends Ecol. Evol.* (2023).

112. Shanmughavel, P. & Francis, K. Above ground biomass production and nutrient distribution in growing bamboo (Bambusa bambos (L.) Voss). *Biomass and Bioenergy* **10**, 383–391 (1996).

113. Shah, S., Sharma, D. P., Pala, N. A., Tripathi, P. & Kumar, M. Temporal variations in carbon stock of Pinus roxburghii Sargent forests of Himachal Pradesh, India. *J. Mt. Sci.* **11**, 959–966 (2014).

114. Pragasan, L. A. & Karthick, A. Carbon stock sequestered by tree plantations in university campus at Coimbatore, India. *Int. J. Environ. Sci.* **3**, 1700–1710 (2013).

115. Becknell, J. M. & Powers, J. S. Stand age and soils as drivers of plant functional traits and aboveground biomass in secondary tropical dry forest. *Can. J. For. Res.* **44**, 604–613 (2014).

116. Ghosh, P. K. & Mahanta, S. K. Carbon sequestration in grassland systems. *Range Manag. Agrofor.* **35**, 173–181 (2014).

117. Návar Cháidez, J. de J., González, N. & Graciano, J. Carbon stocks and fluxes in reforestated sites of Durango, Mexico. *Madera y bosques* **11**, 15–34 (2005).

118. Pragasan, L. A. Tree carbon stock and its relationship to key factors from a tropical hill forest of Tamil Nadu, India. *Geol. Ecol. Landscapes* **6**, 32–39 (2022).

119. Birhane, E., Treydte, A. C., Eshete, A., Solomon, N. & Hailemariam, M. Can rangelands gain from bush encroachment? Carbon stocks of communal grazing lands invaded by Prosopis juliflora. *J. Arid Environ.* **141**, 60–67 (2017).

120. Lorenz, K. & Lal, R. *Carbon Sequestration in Forest Ecosystems*. *Carbon Sequestration in Forest Ecosystems* (2010). doi:10.1007/978-90-481-3266-9.

121. Holtmann, A., Huth, A., Pohl, F., Rebmann, C. & Fischer, R. Carbon sequestration in mixed deciduous forests: The influence of tree size and species composition derived from model experiments. *Forests* **12**, (2021).

122. Vallejo, V. E. *et al.* Effect of land management and Prosopis juliflora (Sw.) DC trees on soil microbial community and enzymatic activities in intensive silvopastoral systems of Colombia. *Agric. Ecosyst. Environ.* **150**, 139–148 (2012).

123. Pandey, C. B., Singh, G. B., Singh, S. K. & Singh, R. K. Soil nitrogen and microbial biomass carbon dynamics in native forests and derived agricultural land uses in a humid tropical climate of India. *Plant Soil* **333**, 453–467 (2010).

124. Bennett, H. H. & Chapline, W. R. Soil erosion a national menace. in *Environmental Geomorphology and Landscape Conservation* 57–83 (Routledge, 2020).

125. Jiao, J., Zou, H., Jia, Y. & Wang, N. Research progress on the effects of soil erosion on vegetation. *Acta Ecol. Sin.* **29**, 85–91 (2009).

126. Okacha, A., Salhi, A., Arari, K., El Badaoui, K. & Lahrichi, K. Soil erosion assessment using the RUSLE model for better planning: a case study from Morocco. *Model. Earth Syst. Environ.* 1–9 (2023).

127. Sharma, N. *et al.* Geospatial technology for assessment of soil erosion and prioritization of watersheds using RUSLE model for lower Sutlej sub-basin of Punjab, India. *Environ. Sci. Pollut. Res.* **30**, 515–531 (2023).

128. Yan, Y. *et al.* Effects of grain-forage crop type and natural rainfall regime on sloped runoff and soil erosion in the Mollisols region of Northeast China. *Catena* **222**, 106888 (2023).

129. Sun, W. *et al.* Effects of forest age on soil erosion and nutrient loss in Dianchi watershed, China. *Environ. Monit. Assess.* **195**, (2023).

130. Chinnasamy, P. & Honap, V. U. Spatiotemporal variations in soil loss across the biodiversity hotspots of Western Ghats Region, India. *J. Earth Syst. Sci.* **132**, (2023).

131. Goswami, K. *et al.* Luminescence chronology of fluvial and marine records from subsurface core in Kaveri delta, Tamil Nadu: Implications to sea level fluctuations. *Geochronometria* **46**, 125–137 (2019).

132. Hero Saharjo, B. & Watanabe, H. Estimation of litter fall and seed production of Acacia mangium in a forest plantation in South Sumatra, Indonesia. *For. Ecol. Manage.* **130**, 265–268 (2000).

133. Black, C. A. Methods of soil analysis, part 1. ASA Inc. Publisher, Madison, Wisconsin, USA. *Agronomy* **9**, 383–390 (1965).

134. Walkley, A. & Black, I. A. An examination of the Degtjareff method for determining soil organic matter, and a proposed modification of the chromic acid titration method. *Soil Sci.* **37**, 29–38 (1934).

135. Subbiah, B. & Asija, G. L. Alkaline permanganate method of available nitrogen determination. *Curr. Sci.* **25**, 259 (1956).

136. Olsen, S. R. *Estimation of available phosphorus in soils by extraction with sodium bicarbonate*. (US Department of Agriculture, 1954).

137. Stanford, G. & English, L. Use of the flame photometer in rapid soil tests for K and Ca. *Agron. J.* **41**, 446–447 (1949).

138. Pathoumthong, P., Zhang, Z., Roy, S. J. & El Habti, A. Rapid non-destructive method to phenotype stomatal traits. *Plant Methods* **19**, 1–9 (2023).

139. Arnon, D. I. Copper enzymes in isolated chloroplasts. Polyphenoloxidase in Beta vulgaris. *Plant Physiol.* **24**, 1 (1949).

140. Farquhar, G. D. & Sharkey, T. D. Stomatal Conductance and Photosynthesis. *Annu. Rev. Plant Physiol.* **33**, 317–345 (1982).

141. Lilley, R. M. & Walker, D. A. An improved spectrophotometric assay for ribulosebisphosphate carboxylase. *Biochim. Biophys. Acta (BBA)-Enzymology* **358**, 226–229 (1974).

142. Rosseel, Y. lavaan: An R package for structural equation modeling. *J. Stat. Softw.* **48**, 1–36 (2012).

143. Chave, J. *et al.* Improved allometric models to estimate the aboveground biomass of tropical trees. *Glob. Chang. Biol.* **20**, 3177–3190 (2014).

144. Negi, J. D. S., Manhas, R. K. & Chauhan, P. S. Carbon allocation in different components of some tree species of India: a new approach for carbon estimation. *Curr. Sci.* **85**, 1528–1531 (2003).

145. Aspinwall, M. J. *et al.* Convergent acclimation of leaf photosynthesis and respiration to prevailing ambient temperatures under current and warmer climates in Eucalyptus tereticornis. *New Phytol.* **212**, 354–367 (2016).

146. Wohlfahrt, G. *et al.* Quantifying nighttime ecosystem respiration of a meadow using eddy covariance, chambers and modelling. *Agric. For. Meteorol.* **128**, 141–162 (2005).

147. Collier, S. M., Ruark, M. D., Oates, L. G., Jokela, W. E. & Dell, C. J. Measurement of greenhouse gas flux from agricultural soils using static chambers. *JoVE (Journal Vis. Exp.* e52110 (2014).

148. Lou, Y., Li, Z., Zhang, T. & Liang, Y. CO2 emissions from subtropical arable soils of China. *Soil Biol. Biochem.* **36**, 1835–1842 (2004).

149. Grissino-Mayer, H. D. A manual and tutorial for the proper use of an increment borer. *Tree-Ring Res.* **59**, 63–79 (2003).

150. Behera, D. K., Jamal, S., Ahmad, W. S., Taqi, M. & Kumar, R. Estimation of Soil Erosion Using RUSLE Model and GIS Tools: A Study of Chilika Lake, Odisha. *J. Geol. Soc. India* **99**, 406–414 (2023).

151. Xu, Z. & Zhou, G. Responses of leaf stomatal density to water status and its relationship with photosynthesis in a grass. *J. Exp. Bot.* **59**, 3317–3325 (2008).

152. Li, G. *et al.* Increasing temperature regulates the advance of peak photosynthesis timing in the boreal ecosystem. *Sci. Total Environ.* **882**, 163587 (2023).

153. Grunstra, M. B. & Van Auken, O. W. Comparative Gas Exchange of Ulmus crassifolia (Cedar Elm, Ulmaceae) and Ungnadia speciosa (Mexican Buckey, Sapindaceae) at Ambient and Elevated Levels of Light, CO2 and Temperature. *Am. J. Plant Sci.* **14**, 691–709 (2023).

154. Qi, Y. *et al.* Applicability of stomatal conductance models comparison for persistent water stress processes of spring maize in water resources limited environmental zone. *Agric. Water Manag.* **277**, 108090 (2023).

155. Jahan, E., Sharwood, R. E. & Tissue, D. T. Effects of leaf age during drought and recovery on photosynthesis, mesophyll conductance and leaf anatomy in wheat leaves. *Front. Plant Sci.* **14**, 1091418 (2023).

156. WU, G. *et al.* An optimized strategy of nitrogen-split application based on the leaf positional differences in chlorophyll meter readings. *J. Integr. Agric.* (2023).

157. Wang, X.-Q., Sun, H., Zeng, Z.-L. & Huang, W. Within-branch photosynthetic gradients are more related to the coordinated investments of nitrogen and water than leaf mass per area. *Plant Physiol. Biochem.* **198**, 107681 (2023).

158. Qu, Y., Mueller-Cajar, O. & Yamori, W. Improving plant heat tolerance through modification of Rubisco activase in C3 plants to secure crop yield and food security in a future warming world. *J. Exp. Bot.* **74**, 591–599 (2023).

159. Wang, Y. *et al.* Effects of plants and soil microorganisms on organic carbon and the relationship between carbon and nitrogen in constructed wetlands. *Environ. Sci. Pollut. Res.* **30**, 62249–62261 (2023).

160. Kim, H. S. Soil erosion modeling using RUSLE and GIS on the Imha watershed, South Korea. (2006).

161. Chen, H., Lewis, L. A. & El Garouani, A. Modeling soil erosion and deposition within a Mediterranean mountainous environment utilizing remote sensing and GIS–Wadi Tlata, Morocco. *Geogr. Helv.* **63**, 36–47 (2008).

162. Williams, J. *et al.* Using soil erosion models for global change studies. *J. Soil Water Conserv.* **51**, 381–385 (1996).

163. Kamaludin, H. *et al.* Integration of remote sensing, RUSLE and GIS to model potential soil loss and sediment yield (SY). *Hydrol. Earth Syst. Sci. Discuss.* **10**, 4567–4596 (2013).

164. Karaburun, A. Estimation of C factor for soil erosion modeling using NDVI in Buyukcekmece watershed. *Ozean J. Appl. Sci.* **3**, 77–85 (2010).

165. Mohammad, A. G. & Adam, M. A. The impact of vegetative cover type on runoff and soil erosion under different land uses. *Catena* **81**, 97–103 (2010).

166. Holdsworth, C. & Morgan, D. *Transitions in context: Leaving home, independence and adulthood: leaving home, independence and adulthood*. (McGraw-Hill Education (UK), 2005).

167. Singh, M. C. *et al.* GIS integrated RUSLE model-based soil loss estimation and watershed prioritization for land and water conservation aspects. *Front. Environ. Sci.* **11**, 1136243 (2023).

168. Sinshaw, B. G. *et al.* Watershed-based soil erosion and sediment yield modeling in the Rib watershed of the Upper Blue Nile Basin, Ethiopia. *Energy Nexus* **3**, 100023 (2021).
